# Chitinolytic Enzymes of the Hyperparasite Fungus *Aphanocladium album*: Genome-Wide Survey and Characterization of A Selected Enzyme

**DOI:** 10.3390/microorganisms11051357

**Published:** 2023-05-22

**Authors:** Claudia Leoni, Caterina Manzari, Matteo Chiara, Pasqua Veronico, Giovanni Luigi Bruno, Graziano Pesole, Luigi R. Ceci, Mariateresa Volpicella

**Affiliations:** 1 Institute of Biomembranes, Bioenergetics and Molecular Biotechnologies, CNR, Via Amendola 165/A, 70126 Bari, Italy; 2 Department of Biosciences, Biotechnology and Enviroment, University of Bari “Aldo Moro”, Via Amendola 165/A, 70126 Bari, Italy; 3 Department of Biosciences, Università degli Studi di Milano, Via Celoria 26, 20133 Milan, Italy; 4 Institute for Sustainable Plant Protection, CNR, Via G. Amendola 122/D, 70126 Bari, Italy; 5 Department of Soil, Plant and Food Sciences, University of Bari “Aldo Moro”, Via Amendola 165/A, 70126 Bari, Italy; 6 Interuniversity Consortium for Biotechnology, Località Padriciano, 99, Area di Ricerca, 34149 Trieste, Italy

**Keywords:** *Aphanocladium album*, genome sequencing, mycoparasite, plant protection, chitinase

## Abstract

The filamentous fungus *Aphanocladium album* is known as a hyperparasite of plant pathogenic fungi; hence, it has been studied as a possible agent for plant protection. Chitinases secreted by *A. album* have proven to be essential for its fungicidal activity. However, no complete analysis of the *A. album* chitinase assortment has been carried out, nor have any of its chitinases been characterized yet. In this study, we report the first draft assembly of the genome sequence of *A. album* (strain MX-95). The in silico functional annotation of the genome allowed the identification of 46 genes encoding chitinolytic enzymes of the GH18 (26 genes), GH20 (8 genes), GH75 (8 genes), and GH3 (4 genes) families. The encoded proteins were investigated by comparative and phylogenetic analysis, allowing clustering in different subgroups. *A. album* chitinases were also characterized according to the presence of different functional protein domains (carbohydrate-binding modules and catalytic domains) providing the first complete description of the chitinase repertoire of *A. album*. A single chitinase gene was then selected for complete functional characterization. The encoded protein was expressed in the yeast *Pichia pastoris*, and its activity was assayed under different conditions of temperature and pH and with different substrates. It was found that the enzyme acts mainly as a chitobiosidase, with higher activity in the 37–50 °C range.

## 1. Introduction

The fungus *Aphanocladium album* is a necrotrophic mycoparasite that efficiently invades the aecidiospores of the rust fungus *Puccinia graminis* [1]. It produces hydrolytic enzymes, especially chitinases [2], which are responsible for the total or partial disruption of the cell walls of many phytopathogenic fungi and parasites [3]. The *A. album* MX-95 strain (patent n 00041374382) was successfully used in the biological control of powdery mildew of tomato caused by *Podosphaera* (*Sphaerotheca*) *fusca* [4] and powdery mildew of cucumber and squash associated with *Golovinomyces* (*Oidium*) *lycopersici* [5]. Moreover, the chitinase Chi1 of *A. album* was identified as an extracellular, chitin-inducible enzyme of possible interest for plant defense strategies against chitin-containing pathogens [6]. More recently, the potential nematicidal activity of *A. album* MX-95 against the root-knot nematode *Meloidogyne javanica* in infected tomato plants was also reported, with appreciable effects on plant protection [7]. Screened as a potential biological limiter, *A. album* MX-95 was considered an active antagonist useful to manage some of the main soil-borne agents of the foot, root, soft rot, and wilt diseases of plants. In fact, in a dual culture plate assay, reducing the growth of *Sclerotinia sclerotiorum* (20%) and *Fusarium solani* (40%) displayed deadlock with mycelial contact against *Fusarium oxysporum* f. sp. *lycopersici* and *Fusarium oxysporum* f. sp. *radicis-lycopersici* and deadlock at a distance versus *Verticillium dahlia*, and it completely overgrew *Phytophthora nicotianae* and *Sclerotinia minor* [8].

Chitin, a linear polymer of N-acetylglucosamine units linked by β-1,4 glycosidic bonds, is largely abundant in living organisms as an integral component of the extracellular matrix of a variety of invertebrates (sponges, mollusks, nematodes, and arthropods) and in the cell walls of filamentous fungi [9]. Chitin hydrolysis has wide relevance not only from the biological point of view but also for the numerous applications of its derivatives, mainly chitosans (natural polymers derived from chitin by deacetylation) and chitosan oligosaccharides (COS) produced by the hydrolysis of chitosan. These compounds are now used in numerous biotechnological applications in medicine, pharmaceutics, chemistry, agriculture, and food [10,11].

Chitin is also the target of possible plant defense strategies. Indeed, plant fungal and insect pathogens are the causative agents of severe plant diseases leading to considerable decreases in crop yields. The degradation of chitin in their cell wall and exoskeleton is thus considered an alternative to the use of chemical fungicides and insecticides [11,12].

In this study, to further explore the potential biotechnological applications of *A. album* MX-95, we determined the first-draft assembly of the fungal genome and performed a careful manual annotation of chitinolytic enzymes coding genes. We described the complete repertoire of chitinolytic enzymes potentially encoded by the *A. album* genome, their main characteristics, and their phylogenetic relationships. The protein codified by the *Chi1* gene was also expressed in the yeast *Pichia pastoris* and purified. The activity of the recombinant protein was assayed at different conditions of temperature and pH, and with different substrates.

## 2. Materials and Methods

### 2.1. Strain and Growth Conditions

*A. album* MX-95 was stored on Potato Dextrose Agar (PDA; Oxoid Part of ThermoFisher Scientific—Microbiology, Hampshire, UK) slant tubes in the culture collection of the University of Bari Aldo Moro—Department of Soil, Plant, and Food Sciences—Plant Pathology Section. Fresh cultures were grown on PDA plates at 25 ± 1 °C in the dark for 12 days. For conidia collection, 20 mL of sterile Oxoid Potato Dextrose Broth (PDB) was poured, and the plate was swirled gently by hand to favor conidia detachment. The conidia concentration was adjusted to 1.5 × 10^7^ conidia/mL. Aliquots (2 mL) were used to inoculate 100 mL of PDB media in 500 mL Erlenmeyer flasks with a cotton cap. Cultures were incubated at 25 ± 1 °C under 120 rpm of orbital shaking for 5 days. The mycelial mat was collected by centrifugation at 8000× *g* at 4 °C for 20 min and lyophilized.

### 2.2. DNA Isolation and Sequencing

The Plant/Fungi DNA Isolation Kit (Sigma-Aldrich, Part of Merck KGaA, Darmstadt, Germany, cod. E5038) was used for the purification of DNA from *A. album* according to the manufacturer’s instructions. The final product was about 100 µL of application-ready DNA. The quality and concentration of the DNA were determined by agarose gel electrophoretic analysis and by spectrophotometric measurements at 260, 280, and 230 nm using the NanoDrop ND-1000 Spectrophotometer (ThermoFisher Scientific Inc., Milan, Italy).

DNA was subjected to whole-genome shotgun sequencing using Nextera XT technology (Illumina, San Diego, CA, USA), and 2 × 250-nucleotide paired-end reads were generated on an Illumina MiSeq instrument.

### 2.3. Genome Assembly Annotation

Reads were subjected to quality trimming by Trimmomatic [13] software (version 0.32). The following parameters were applied: ILLUMINACLIP, SLIDINGWINDOW <10,20>, HEADCROP <10>, MINLEN <100>. Pear [14] software (version 0.9), with the parameters: -v 10, -m 190, -v 20, was subsequently applied to merge overlapping pairs of reads. SPAdes [15] was used to perform genome assembly. The following kmers size was used for the construction of de Brujin graphs: 55,77,99,121. The in silico prediction of protein-coding genes was performed by applying AUGUSTUS “https://bioinf.uni-greifswald.de/augustus/ (accessed on 15 November 2021)” [16] with *Saccharomyces cerevisiae* gene models. PFAM (Protein Families) domains were annotated to predict protein-coding genes with the PfamScan program “https://www.ebi.ac.uk/Tools/pfa/pfamscan (accessed on 15 November 2021)” [17] using both the PFAM-A and PFAM-B domain models. The completeness of the assembly was checked with a set of 1315 highly conserved metazoan genes (OrthoDB version 9.1) using BUSCO v3.0.2.

### 2.4. Analysis of Protein Sequences 

Candidate genes for chitinolytic enzymes in the *A. album* genome were identified as those containing a “Glyco_Hydro_3 (or 18, 20, 75)” protein domain according to the PFAM annotation and the CAZy classification of glycoside hydrolase (GH) families [18]. Sequences belonging to the GH3, GH18, GH20, and GH75 families were then manually extracted.

Sequence alignment was carried out by Clustal Omega “https://www.ebi.ac.uk/Tools/msa/clustalo (accessed on 15 November 2021)” [19]. Phylogenetic analysis was performed by MEGA11 [20] using the Clustal multialignments as input files and applying the Maximum-Likelihood method and the Jones–Taylor–Thornton model for amino acid substitutions. Bootstrap support values were estimated using 1000 pseudo-replicates. Protein domains were annotated using the HMMER service “http://www.ebi.ac.uk/Tools/hmmer (accessed on 15 November 2021)” [21]. Three-dimensional modeling was obtained by Phyre2 “http://www.sbg.bio.ic.ac.uk/phyre2 (accessed on 15 November 2021)” analysis [22].

### 2.5. Gene Cloning, Expression, and Purification of rChi1

The Chi1 protein (P32470) corresponds to the polypeptide KAJ6787487.1 encoded in the genome of *A. album* MX-95 (gene accession number JARDXD010000528.1 in the NCBI GenBank database) (see Section 3.7). Since the Chi1 polypeptide includes a 34-amino acid presequence [2,6], only the portion of the gene coding for the mature protein was cloned. A synthetic gene (deduced from the *A. album* MX-95 gene JARDXD010000528.1) was prepared by the Gene Universal Company (Newark, DE, USA) according to the codon usage of *P. pastoris* and cloned into the *EcoRI*/*NotI* restriction sites of the pPICZα expression vector. The *SacI* linearized recombinant vector was then used to transform *P. pastoris* GS115 cells according to the Invitrogen protocol. The expression of the recombinant Chi1 (rChi1) protein was performed following established procedures [23], and its expression was verified by Western blot using the Mouse Anti-Penta Histidine Tag: HRP (Bio-Rad, Hercules, CA, USA) antibody. rChi1 was purified by affinity chromatography on a HisTrap-HP column (GE Healthcare, Chicago, IL, USA) by a stepwise procedure using an elution buffer (EB) containing 0.5 M NaCl, 11.6 mM Na_2_HPO_4_, 8.4 mM NaH_2_PO_4_ (pH 7), and increasing imidazole concentrations (10, 50, 100, and 500 mM). The rChi1 protein, eluted with EB plus 100 mM imidazole, was dialyzed in 1% DPBS buffer (137 mM NaCl, 2.7 mM KCl, 1.47 mM KH_2_PO_4_, and 6.6 mM K_2_HPO_4_ pH7), and the protein concentration was determined using the Bradford assay kit (Bio-Rad).

### 2.6. Chitinase Activity Assay

Two activity assays were carried out. The first assay was performed by incubating 2.5 μg of rChi1 with 1.5 mg of chitin-azure substrate (Sigma-Aldrich cod. C3020-1G) for 3 h at 37 °C in DPBS buffer with 10 mM MgCl_2_ [24]. A second activity assay was carried out with the Chitinase Assay kit (Sigma-Aldrich cod. CS0980) in the presence of 2 μg of enzyme and 50 μg of one of three substrates, 4-Nitrophenyl N,N′-diacetyl-β-D-chitobioside, 4-Nitrophenyl N-acetyl-β-D-glucosaminide, or 4-Nitrophenyl β-D-N,N′,N″-triacetylchitotriose, according to the kit instructions. All the assays were performed in triplicate.

## 3. Results and Discussion

### 3.1. A. album DNA Sequencing and Genome Assembly

Total *A. album* Mx-95 genomic DNA was purified from 100 mL of culture by using the Plant/Fungi DNA Isolation Kit (Sigma-Aldrich cod. E5038) at a final concentration of 100 ng/μL (with 260/280 and 260/230 absorbance ratios of 1.9 and 1.8, respectively). DNA was subjected to whole-genome shotgun sequencing. A total of 6,368,454 paired-end (PE) reads of 2 × 250 bp were produced using an Illumina MiSeq platform. The average insert size was estimated at 423 bp. After applying strict quality filtering and read trimming, 5,431,102 high-quality pairs of reads with an average read length of 146.12 bp were retained. Of these, 1,431,102 were overlapped by 10 or more bp, and they were concatenated by Pear [14] software.

The genome was assembled using SPAdes. The final assembly contained 1941 scaffolds longer than 250 bp, with an estimated size of 35,125,516 bp and a GC content of 52.95%. The N50 value of the assembly was 38,109 bp, and the longest scaffold was 226 kb. The genome sequence was deposited in NCBI GenBank “https://www.ncbi.nlm.nih.gov/genbank (accessed on 15 March 2023)” with the accession number JARDXD000000000.

Interestingly, according to the bowtie2 software [25], only 75.56% of the 5,431,102 PEs that passed the quality filters had the best unique mapping positions on our draft assembly of the *A. album* genome, while 22.36% were assigned to two or more distinct genomic positions; this observation might be consistent with a relatively high content of repeats in the genome of *A. album*. Only 2.08% of the reads did not map on the draft assembly, potentially suggesting somewhat low levels of DNA contamination.

De novo genome prediction of gene models identified a total of 11,292 protein-coding genes in the *A. album* genome. These included 1289 (98.1%) of the 1315 conserved Ascomycota Benchmarking with Universal Single-Copy Orthologs (BUSCO) groups (BUSCO, RRID:SCR_015008) [26], as defined by the orthodb V9 database [27], indicating that the obtained draft genome assembly should provide a nearly complete representation of *A. album* protein-coding genes. A total of 8080 predicted protein-coding genes were associated with one or more PFAM protein domains. Among these, 167 contained PFAM domains compatible with GH enzymes and were subjected to careful manual annotation.

### 3.2. Chitinases in A. album: Genome Analysis and Protein Structural Analysis

The list of predicted protein-coding genes in the *A. album* genome was surveyed for the presence of genes coding for chitinolytic enzymes. This class of enzymes belongs to the super-family of glycoside hydrolases (GH), which is subdivided into specific structural families in the CAZy database “https://www.cazy.org (accessed on 25 November 2021)” [18]. Although chitinolytic enzymes in fungi have been detected in a wide range of GH families [11,28,29], *A. album* genes annotated by PFAM as belonging to GH families containing chitinolytic enzymes were restricted to the GH3, GH18, GH20, and GH75 families. Accordingly, all the *A. album* genes annotated in these families were selected and investigated by multialignment and phylogenetic analysis, domain prediction, and structural modeling. Multialignment was obtained by Clustal Omega [19], domain prediction was carried out by HMMER [21], and 3D modeling was performed by Phyre2 software [22]. The GH18 family was the most represented (twenty-six genes), followed by the GH3 family (twelve genes), GH20 (nine genes), and GH75 (eight genes). Table 1 reports the encoded polypeptides classified according to the GH families and possible enzyme activity. The identification of prospective distinct protein groups with specific activities within each of the four CAZy families is consistent with the wide functional activity and physiological role of these enzymes in *A. album*.

### 3.3. GH18 Enzymes

The GH18 family contains chitinases (EC 3.2.1.14) of Classes III and V, characterized by a (β/α)_8_ barrel structure, a glycosidic bond retaining hydrolytic mechanism, and a glutamic acid catalytic proton donor [11] (characteristics also common to the chitinolytic enzymes of the GH3 and GH20 families [18]).

Class III enzymes have a shallow and open substrate binding groove that is believed to support their endochitinase activity. Class V enzymes are characterized by a deep, tunnel-shaped substrate-binding groove corresponding to exo-acting activity [28]. Enzymes of both classes share the consensus sequence DXXDXDXE, containing the catalytic glutamic acid, although low levels of similarity are shared between the two classes. On the basis of phylogenetic analysis, GH18 chitinases from *Trichoderma reesei* and other filamentous fungi can be divided into three groups: A, B, and C [30]. While A and B chitinases correspond to Class V and III, respectively, chitinases of group C have a higher molecular weight and show a domain structure similar to that of *Kluyveromyces lactis* killer toxins [31]. Group C chitinases are present only in filamentous ascomycetes and are expected to have exochitinase activity, although no enzymes from this group have yet been characterized [32].

**Figure 1:**
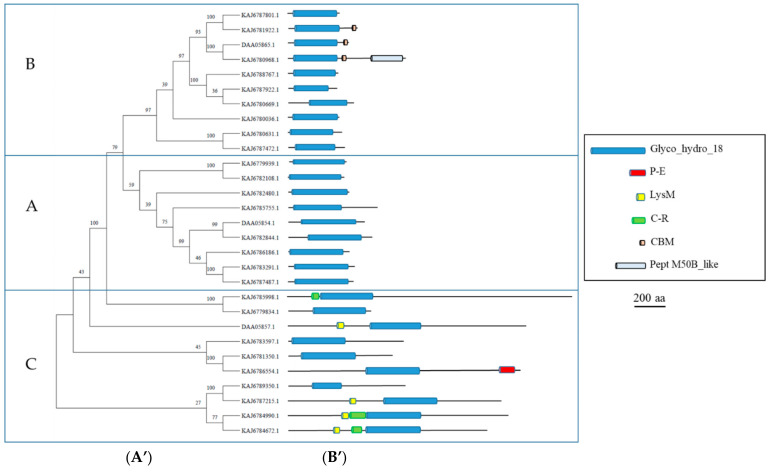
Phylogenetic and structural analyses of *A. album* GH18 chitinases. (**A’**) Phylogenetic tree of the *A. album* GH18 chitinases, indicated by their accession number, and three GH18 sequences from *H. jecorina* (DAA05865, DAA05854, DAA05857). (**A**–**C**) indicate the three subgroups of fungal GH18 chitinases [30]. Phylogenetic analysis was carried out by MEGA11. Bootstrap values are reported close to nodes. (**B’**) Graphical representation of the functional domains identified by HMMER in the chitinase sequences (description in the figure inset). Details on the precise location and length of the identified regions are reported in Table S1. Insert legend (corresponding PFAM IDs are reported in brackets): Lys = LysM domain (PF01476); C-R = Chitin recognition protein (PF00187); GH18 = Glycosyl hydrolases family 18 (PF00704); CBM = Fungal cellulose-binding domain (PF00734); Pept_M508 = Peptidase M50B-like (PF13398); P-E = Pathogen effector; putative necrosis-inducing factor (PF14856).

The classification of *A. album* GH18 chitinases was carried out by including three *H. jecorina* chitinases representative of the A, B, and C groups (DAA05854, DAA05865, and DAA05857, respectively) (Figure 1). Three major clusters of sequences could be clearly distinguished, each containing one of the *H. jecorina* representative sequences, confirming the presence of the three groups of chitinases in *A. album*. In particular, the group A and C sequences contained larger GH18 domains (see Table S1 for details). They included a typical extra α/β region of about 70 amino acids described in plant and fungal Class V chitinases [1,33] (multialignment is reported in the Supplementary Material, Figure S1). The presence of chitin recognition and binding domains (C-R) and LysM motifs, observed in some *A. album* sequences, was also typical of group C endochitinases [30]. Group B proteins were characterized by shorter GH18 domains (see Table S1 for details) and the presence of four cysteine residues involved in the formation of disulfide bridges in most of them, which was also described in plant Class III chitinases [34].

### 3.4. GH3 Enzymes

The GH3 family contained β-N-acetylhexosaminidases (EC 3.2.1.52), exo-chitinases that hydrolyze glucosidic bonds starting from the non-reducing end of chitin molecules and yielding N-acetylglucosamine monomers. These enzymes are characterized by a (β/α)_8_ barrel structure, a glycosidic bond retaining hydrolytic mechanism, and a glutamic acid catalytic proton donor [11]. Although β-N-acetylhexosaminidases were originally considered specific to bacteria [35], they have recently also been described in fungi. The first fungal β-N-acetylhexosaminidase was identified in *Rhizomucor miehei* [36] and contained the highly conserved motif KHF(I)PGH(L)GXXXXDS(T)H. The enzyme was found to be active in the hydrolysis of N-acetyl-chitooligosaccharides with degrees of polymerization of 2–5. More recently, two other putative fungal GH3 β-N-acetylhexosaminidase genes were found in the genome of the entomopathogenic fungus *Metarhizium anisopliae* through extensive sequence and structural analysis [37]. Other GH3 enzymes encoded in the *M. anisopliae* genome, which could not be classified as β-N-acetylhexosaminidases, showed higher sequence identities with fungal β-glucosidases (EC 3.2.1.21). The analysis of other fungal genomes carried out in the same study also allowed the identification of other putative GH3 β-N-acetylhexosaminidases.

**Figure 2:**
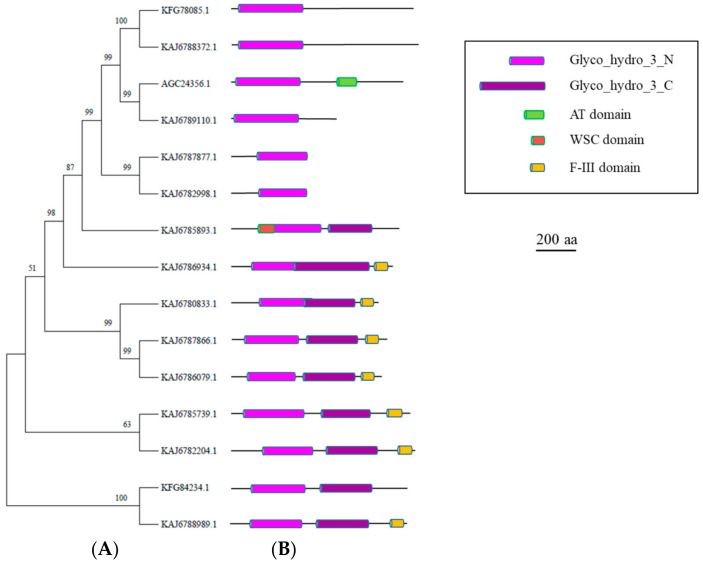
Phylogenetic and structural analyses of *A. album* GH3 sequences. (**A**) Phylogenetic tree of the *A. album* GH3 sequences, indicated by their gene number, the GH3 β-N-acetylhexosaminidases described in *R. miehei* (AGC24356) and *M. anisopliae* (KFG78085), and a GH3 β-glucosidases (KFG84234) from *M. anisopliae*. Bootstrap values are reported close to nodes. (**B**) Graphical representation of the functional domains identified in the protein sequences (description in the figure inset). Details on the precise location and length of the identified regions are reported in Table S2. Insert legend (PFAM IDs are reported in brackets): WSC = WSC domain for carbohydrate binding (PF01822); GH3-N = Glycosyl Hydrolase family 3 N-terminal domain (PF00933); GH3-C = Glycosyl Hydrolase family 3 C-terminal domain (PF01915); AT = Acetyl Transferase (GNAT) family (PF00583); F-III = Fibronectin type III-like domain (PF14310).

Twelve candidate GH3 genes were identified in the *A. album* genome (Table 1). In order to derive an accurate classification of the encoded enzymes, the *A. album* sequences and a collection of other GH3 β-N-acetylhexosaminidases from other fungi (*R. miehei* (AGC24356), *M. anisopliae* (KFG78085) and one of the five *M. anisopliae* GH3 β-glucosidases (KFG84234)) were subjected to a thorough phylogenetic analysis. Figure 2 shows the corresponding phylogenetic tree, as obtained by MEGA11 analysis, together with the functional domains identified by HMMER. Sequence multialignment is reported in the Supplementary Material (Figure S2). Multialignment and phylogenetic analysis indicated that the *A. album* sequences 2908 and 8944 could be clearly classified among the β-N-acetylhexosaminidases. Indeed, both clustered with homologous enzymes from *M. anisopliae* and *R. miehei*, respectively (Figure 2). It must be noted, however, that the consensus motif KHF(I)PGH(L)GXXXXDS(T)H was fully conserved only in AGC24356 and 8944 (Supplementary Figure S2), suggesting that the inclusion of additional sequences might be required to derive a more accurate consensus for this motif. Interestingly, the 8934 and 9753 sequences also belong to the same phylogenetic clade. Their classification as possible β-N-acetylhexosaminidases was further confirmed by the 3D modeling results obtained by Phyre2 [22]. Indeed, both the 8934 and 9753 sequences formed 100% confident models based on the 3D structure of a β-N-acetylglucosaminidase from *Paenibacillus* sp. str. FPU-7 (PDB 6K5J).

According to the phylogenetic analyses, the 2585 protein is closely related to the *M. anisopliae* β-glucosidase and should be classified as a possible β-glucosidase, which was also confirmed by the Phyre2 analysis (Table S5). Similarly, 6376, 3709, 76, 520, 7154, and 9852 also corresponded to possible β-glucosidases according to the Phyre2 analysis. Finally, 7919 had the best fit with a β-xylosidase (E.C. 3.2.1.37) from *Phanerochaete chrysosporium* [38] according to the Phyre2 results. Table S5 reports the results of the Phyre2 analysis for all *A. album* GH3 sequences.

### 3.5. GH20 Enzymes

**Figure 3:**
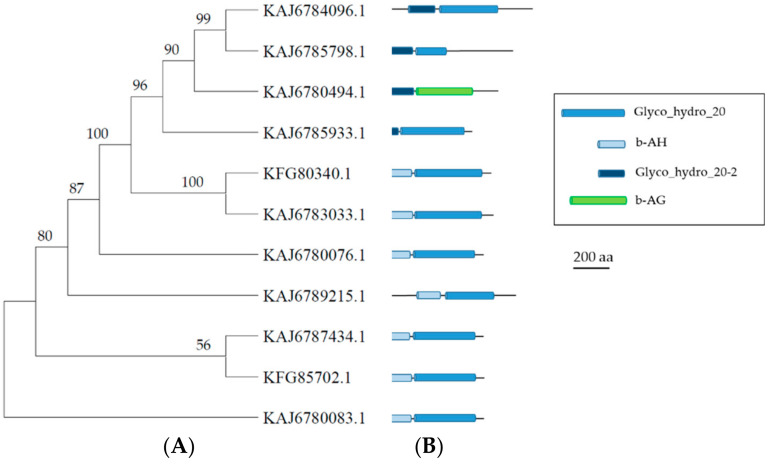
Phylogenetic and structural analyses of *A. album* GH20 sequences. (**A**) Phylogenetic tree of the *A. album* GH20 sequences, indicated by their gene number, and two *M. anisopliae* sequences, KFG80340 and KFG85702. Bootstrap values are reported close to nodes. (**B**) Graphical representation of the functional domains identified in the protein sequences (description in the figure inset). Details on the precise location and length of the identified regions are reported in Table S3. Insert legend (PFAM IDs are reported in brackets): GH20-2 = Glycosyl Hydrolase family 20 domain 2 (PF02838); β-AH = β-acetylhexoaminidase-like (PF14845); GH20 = Glycosyl Hydrolase family 20 catalytic domain (PF00728); β-AG = β-N-acetylglucosaminidase (PF07555).

Similar to GH3, the GH20 family also contained β-N-acetylhexosaminidases (EC 3.2.1.52), characterized by a (β/α)_8_ barrel structure and a glycosidic bond retaining hydrolytic mechanism [11], and particularly abundant in fungi [37]. Large-scale analyses of GH20 β-N-acetylhexosaminidases in prokaryotes and eukaryotes allowed the identification of a conserved motif H(N)XGA(C/G/M)DEA(I/L/V) containing the catalytic residue DE [35]. Recently, analysis of GH20 β-N-acetylhexosaminidases potentially encoded by the genome of the Ascomycota *M. anisopliae* resulted in the identification of two genes (KFG80340 and KFG85702) corresponding to two distinct phylogenetic groups that are also common to other ascomycetes [37]. It must be noted that the sequences belonging to one of the two clades contained a slight variation of the conserved motif around the catalytic residues, corresponding to H(N)XGA(C/G/M)DEA(I/L/V/Y). The multialignment of the nine *A. album* sequences and the two *M. anisopliae* sequences associated with the GH20 family is reported in the Supplementary Material (Figure S3). Figure 3 shows the corresponding phylogenetic tree obtained by the MEGA11 analysis. together with the functional domains identified by HMMER. *A. album* sequence 1855 shared the motif HVGGDEL with *M. anisopliae* KFG80340, while sequences 10436, 3274, and 1012 shared the motif HTGGDEY with *M. anisopliae* KFG85702. Sequence 1005 had a slightly different HTGGDEV motif. On the contrary, *A. album* sequences 6860, 6931, 1307, and 2970 did not share a fully conserved motif around the catalytic residues. These sequences formed a separate clade in the phylogenetic tree. Phyre2 analysis showed the highest rankings with bacterial homologs for sequences 6860, 6931, and 1307, while for sequence 2970, the match with the highest score was obtained with a GH84 hydrolase from *Clostridium perfringens* (see Supplementary Table S6).

### 3.6. GH75 Enzymes

**Figure 4:**
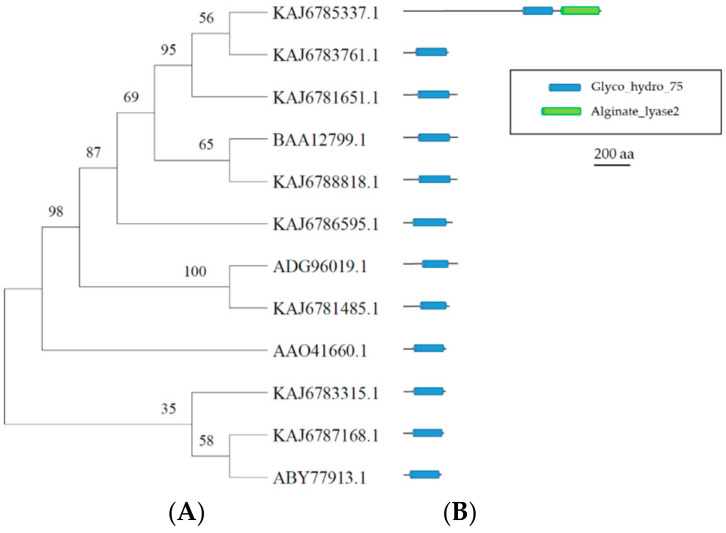
Phylogenetic and structural analyses of *A. album* GH75 sequences. (**A**) Phylogenetic tree of the *A. album* GH75 sequences, indicated by their gene number, and the chitosanases from *A. fumigatus* (AAO41660), *F. solani* (BAA12799), *G.* sp. JG-2005 (ABY77913), and *P. chrysogenum* (ADG96019.1). Bootstrap values are reported close to nodes. (**B**) Graphical representation of the functional domains identified in the protein sequences (description in the figure inset). Details on the precise location and length of the identified regions are reported in Table S4. Insert legend (PFAM IDs are reported in brackets): GH75 = Fungal chitosanase of glycosyl hydrolase group 75 (PF07335); A–L = Alginate Lyase (PF08787).

The GH75 family contains chitosanases (E.C. 3.2.1.132) characterized by a glycosidic bond inverting hydrolytic mechanism [11]. They catalyze the endohydrolysis of the β-1,4-linkages of chitosans, which naturally occur in the cell wall of Zygomycetes fungi and algae and insect cuticles [29]. The CAZy GH75 family contains a relatively small number (142) of fungal sequences, of which only twelve have been characterized. No 3D structures have been determined so far. The fungal GH75 chitosanases carry two highly conserved motifs (25)NMDIDCD(31) and (113)YGIWGD(118) (numbering according to the chitosanase from *Penicillium* sp. D-1 [39]). The multialignment of the eight *A. album* GH75 sequences and the twelve chitosanases characterized in *Aspergillus clavatus* (AJG44374), *A. fumigatus* (EAL84291, CAE54966.1, and AAO41660), *A. oryzae* (BAD08218 and BAA92250), *Aspergillus* sp. CJ22-326 (ABZ88800), *Fusarium solani* (ABX57824 and BAA12799), *Gongronella* sp. JG-2005 (ABY77913), *Penicillium chrysogenum* (ADG96019.1), and *P.* sp. D-1 (AFG33049) is reported in Supplementary Material Figure S4. All the sequences share conserved motifs highly similar to those proposed for the fungal chitosanases, specifically, N(D)M(L)DI(V)D(N)CDG and YGI(V)W(F/Y)GD together with a fully conserved Glu(140) residue. Figure 4 shows the phylogenetic tree obtained by MEGA 11 software and the functional domains identified by the HMMER analysis, limited to the *A. album* sequences and four representative fungal GH75 sequences.

### 3.7. Expression and Activity Assays of the rChi1 Protein

Chi1 was described and characterized as the major exochitinase produced by an *A. album* chitinase-overproducing mutant derived from the ETHM 483 strain [40]. Its sequence was not determined, but the purified protein allowed the production of an antiserum, which was subsequently used for the screening of a cDNA expression library from the same mutant [2]. Among the numerous positive cDNA fragments, a specific 228 bp fragment, possibly corresponding to the 3′ terminus of the Chi1 cDNA, was identified and used for the screening of an *A. album* genomic DNA library, leading to the identification of a positive 8-Kbp genomic fragment. The genomic fragment was then cloned into the filamentous fungus *F. oxysporum*, whose culture supernatant showed increased chitinolytic activity, but the recombinant Chi1 protein was not further characterized. The complete sequences of the Chi1 cDNA and gene were finally described in an additional paper [6].

In the present study, we identified the gene encoding the Chi1 protein in the *A. album* MX-95 genome. The encoded polypeptide (KAJ6787487.1) had the same length as the Chi1 enzyme first identified in the ETHM 483 strain of *A. album* [2]. The two polypeptides differed only at positions 375 (T in P32470, S in KAJ6787487.1) and 383 (S in P32470, D in KAJ6787487.1). These differences can be explained by taking into account the different strains of *A. album* in which they were identified. The mature form of the polypeptide KAJ6787487.1 (deduced by comparison with the native *A. album* ETHM 483 Chi1 protein) was also expressed as a recombinant protein and characterized. This is the first complete study on an *A. album* chitinase, from its gene sequencing to the characterization of the recombinant protein. It clarifies the fragmentary results of previous work and provides evidence that the reported multiple positive signals obtained in immunoassays can be explained by the presence of different chitinases encoded in the same genome.

The recombinant Chi1 mature polypeptide corresponding to the protein KAJ6787487.1 was expressed by the *P. pastoris* GS115 system for the secretion of recombinant proteins. The expressed protein also contained 29 additional amino acids (including a six-His tail) encoded by the pPICZα vector, resulting in a final molecular weight of 45.92 kDa. The expression of the recombinant enzyme was confirmed by Western blot using an anti-His antibody (Figure S5).

The recombinant protein was purified by Ni-affinity chromatography using a discontinuous imidazole gradient. The protein was mostly present in the fractions obtained using 100 mM imidazole (Figure S6), which were collected and subjected to dialysis against DPBS buffer. The protein was visualized by SDS-PAGE (Figure S7), and its concentration was evaluated by the Bradford procedure. Typically, the protein yield was 1.2 μg/L of *P. pastoris* culture.

The activity of purified rChi1 was evaluated by the hydrolysis of the chitin-azure substrate, which was followed spectrophotometrically at 560 nm. The assay was carried out at different temperatures (37 °C, 50 °C, and 70 °C at pH 7) and pH values (6, 7, and 8 at 37 °C) with 3 h of incubation. The higher rChi1 activity (0.63 ± 0.08 U/μg) was obtained at 37 °C and pH 8 (Table 2).

**Figure 5:**
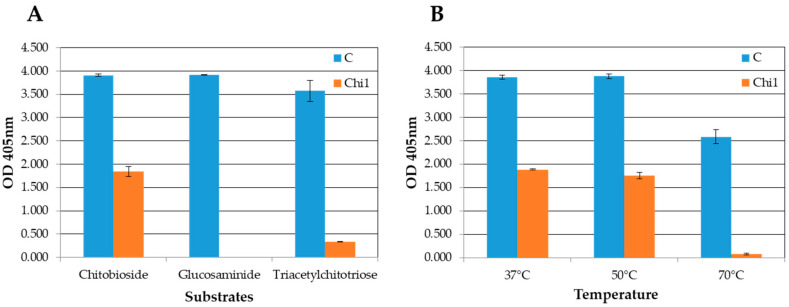
Chitinase activity assays of rChi1. Histograms show optical density (OD) at 405 nm of the rChi1 assays on the three substrates (**A**) and at different temperatures using the 4-Nitrophenyl N,N′-diacetyl-β-D-chitobioside as the substrate (**B**). Abbreviations for substrates are as follows: Chitobioside for 4-Nitrophenyl N,N′-diacetyl-β-D-chitobioside; Glucosaminide for 4-Nitrophenyl N-acetyl-β-D-glucosaminide; Triacetylchitotriose for 4-Nitrophenyl β-D-N,N′,N′-triacetylchitotriose.

The chitinolytic activity of rChi1 was also assayed using three alternative substrates: 4-Nitrophenyl β-D-N,N′,N″-triacetylchitotriose (specific for endochitinase activity), 4-Nitrophenyl N,N′-diacetyl-β-D-chitobioside (suitable for exochitinase/chitobiosidase activity), and 4-Nitrophenyl N-acetyl-β-D-glucosaminide (for N-acetylglucosaminidase activity). The hydrolysis of the 4-nitrophenyl group can be followed by absorbance at 405 nm, allowing the determination of the enzyme activity. The *Trichoderma viride* chitinase was used as a control at 0.2 mg/mL. The assays showed that rChi1 is an exochitinase with chitobiosidase activity. Indeed, it was more active against the substrate 4-Nitrophenyl N,N′-diacetyl-β-D-chitobioside, less active against 4-Nitrophenyl β-D-N,N′,N″-triacetylchitotriose, and did not show any activity against 4-Nitrophenyl N-acetyl-β-D-glucosaminide (Figure 5A). The specific activity of rChi1 using 4-Nitrophenyl N,N′-diacetyl-β-D-chitobioside as substrate was 0.10 ± 0.01 U/μg. The specific activity was calculated according to the manual of the Chitinase Assay kit by Sigma: one unit of enzyme is the amount of enzyme that hydrolyzes 1.0 μmole di p-NO_2_ per minute at pH 4.8 and 37 °C.

A second assay was carried out at different temperatures using 4-Nitrophenyl N-N′-diacetyl-β-D-chitobioside as the substrate. rChi1 showed about the same activity at 37 °C and 50 °C, while at 70 °C, the activity decreased (Figure 5B).

The determined chitobiosidase activity of rChi1 was in agreement with its classification as a group A exochitinase of the GH18 family (Figure 1).

## 4. Conclusions

The sequencing of the whole genome of the mycoparasite fungus *A. album* allowed the complete survey of the genes encoding chitinolytic enzymes. The fungus contained a potentially large array of chitinolytic enzymes (46) belonging to the four GH families in which fungal chitinases can be classified. The enzymes had either endochitinase activity (group B enzymes of the GH18 family) or exochitinase activity (enzymes of groups A and C of the GH18 family and β-N-acetylhexosaminidases of the GH3 and GH20 families). Additionally, the enzymes identified in the GH75 family had endohydrolytic activity on chitosans. This large array of enzymes allows the fungus to perform several physiological functions of a typical filamentous species requiring chitinolytic activities, including interactions with other fungi, the degradation of exogenous chitin for nutrient acquisition, cell wall remodeling, and hyphal growth [32]. Further studies are required to identify the specific functions of single enzymes, which will also contribute to better understanding the potential use of *A. album* in plant defense. In this study, we further characterized the Chi1 chitinase, which had already been identified as a secreted enzyme from *A. album* cultures grown on chitin as a carbon source. Structural and phylogenetic analyses allowed the classification of the enzyme in group A of the GH18 exochitinases. The specific activity was subsequently confirmed using 4-Nitrophenyl N,N′-diacetyl-β-D-chitobioside as a substrate.

## Figures and Tables

**Table 1:** Encoded chitinolytic enzymes of *A. album* MX-95. ID indicates the NCBI GenBank accession number of the polypeptides.

CAZy Family	ID	Amino Acids	Activity
GH18	KAJ6784672.1	1279	Exochitinase
	KAJ6786186.1	394	“
	KAJ6780036.1	330	Endochitinase
	KAJ6780669.1	423	“
	KAJ6787801.1	336	“
	KAJ6780631.1	348	“
	KAJ6783291.1	428	Exochitinase
	KAJ6787472.1	365	Endochitinase
	KAJ6787487.1	423	Exochitinase
	KAJ6781350.1	671	“
	KAJ6782844.1	540	“
	KAJ6788767.1	324	Endochitinase
	KAJ6784990.1	1418	Exochitinase
	KAJ6785755.1	574	“
	KAJ6779939.1	369	“
	KAJ6780968.1	757	Endochitinase
	KAJ6787215.1	1373	Exochitinase
	KAJ6786554.1	1495	“
	KAJ6781922.1	445	Endochitinase
	KAJ6785998.1	1832	Exochitinase
	KAJ6782480.1	392	“
	KAJ6782108.1	363	“
	KAJ6789350.1	754	“
	KAJ6779834.1	532	“
	KAJ6783597.1	742	“
	KAJ6787922.1	315	Endochitinase
GH3	KAJ6786934.1	830	β-glucosidase
	KAJ6780833.1	760	“
	KAJ6788989.1	883	“
	KAJ6788372.1	932	β-N-acetylhexosaminidase
	KAJ6782204.1	948	β-glucosidase
	KAJ6785739.1	893	“
	KAJ6787866.1	802	“
	KAJ6785893.1	870	β-xylosidase
	KAJ6787877.1	370	β-N-acetylhexosaminidase
	KAJ6789110.1	539	“
	KAJ6782998.1	369	“
	KAJ6786079.1	777	β-glucosidase
GH20	KAJ6780076.1	577	β-N-acetylhexosaminidase
	KAJ6780083.1	579	“
	KAJ6785933.1	514	“
	KAJ6783033.1	633	“
	KAJ6780494.1	661	GH 84 hydrolase
	KAJ6787434.1	574	β-N-acetylhexosaminidase
	KAJ6784096.1	848	“
	KAJ6785798.1	741	“
	KAJ6789215.1	751	“
GH75	KAJ6781651.1	299	Chitosanase
	KAJ6783315.1	232	“
	KAJ6785337.1	1093	“
	KAJ6781485.1	256	“
	KAJ6788818.1	300	“
	KAJ6783761.1	252	“
	KAJ6786595.1	273	“
	KAJ6787168.1	234	“

**Table 2:** Specific activities of rChi1 on chitin-azure substrate.

pH	T (°C)	rChi1 Specific Activity (U/μg) *
7	37	0.51 ± 0.11
7	50	0.39 ± 0.03
7	70	0.25 ± 0.13
6	37	0.18 ± 0.12
7	37	0.54 ± 0.02
8	37	0.63 ± 0.08

* Specific activity was calculated according to Ramirez et al. [24]: one unit of chitinase is the amount of enzyme that produces an increase of 0.01 in the absorbance at 560 nm after 3 h at pH 7 and 50 °C.

## Data Availability

Sequencing data are available at NCBI GenBank with the accession number JARDXD000000000.

## Supplementary Materials

The following supporting information can be downloaded at: https://www.mdpi.com/article/10.3390/microorganisms11051357/s1, Table S1. HMMER prediction of the main domains in GH18 sequences. Table S2. HMMER prediction of the main domains in GH3 sequences. Table S3. HMMER prediction of the main domains in GH20 sequences. Table S4. HMMER prediction of the main domains in GH75 sequences. Table S5. Summary of the Phyre2 analysis for the GH3 sequences of the *A. album*. Table S6. Summary of the Phyre2 analysis for the GH20 sequences of the *A. album*. Figure S1. Clustal alignment of *A. album* GH18 chitinases. Figure S2. Clustal alignment of *A. album* GH3 sequences. Figure S3. Clustal alignment of *A. album* GH20 sequences. Figure S4. Clustal alignment of *A. album* GH75 sequences. Figure S5. Expression of recombinant Chi1 mature protein. Figure S6. SDS-PAGE (15% polyacrylamide gel) analysis of chromatographic fractions. Figure S7. SDS-PAGE (15% polyacrylamide gel) of the purified Chi1 recombinant protein.
