# Supplementary material for: Chitinolytic Enzymes of the Hyperparasite Fungus Aphanocladium album: Genome-Wide Survey and Characterization of A Selected Enzyme
Source: Microorganisms. 2023 May 22;11(5):1357. doi: 10.3390/microorganisms11051357 (PMC10223446; doi:10.3390/microorganisms11051357)
Supplement: Supplementary file 1 [file microorganisms-11-01357-s001.zip › microorganisms-2378450-supplementary.pdf]

SUPPLEMENTARY MATERIAL

Chitinolytic enzymes of the hyperparasite fungus *Aphanocladium album*: genome-wide survey and characterization of a selected enzyme.

Claudia Leoni [1], Caterina Manzari [2], Matteo Chiara [3], Pasqua Veronico [4], Giovanni Luigi Bruno [5], Graziano Pesole [1,2,6], Luigi R. Ceci [1,*] and Mariateresa Volpicella [2,*]

[1] Institute of Biomembranes, Bioenergetics and Molecular Biotechnologies, CNR, Via Amendola 165/A, 70126 Bari, Italy
[2] Department of Biosciences, Biotechnologies and Biopharmaceutics, University of Bari "Aldo Moro", Via Amendola 165/A, 70126 Bari, Italy
[3] Department of Biosciences, Università degli Studi di Milano, Via Celoria 26, 20133 Milan, Italy
[4] Institute for Sustainable Plant Protection, CNR, Via G. Amendola 122/D, 70126 Bari, Italy
[5] Department of Soil, Plant and Food Sciences, University of Bari "Aldo Moro", Via Amendola 165/A, 70126 Bari, Italy
[6] Interuniversity Consortium for Biotechnology, Località Padriciano, 99, Area di Ricerca, 34149 Trieste, Italy
* Correspondence: l.ceci@ibiom.cnr.it (L.R.C.); mariateresa.volpicella@uniba.it (M.V.)

CONTENT

Table S1. HMMER prediction of the main domains in GH18 sequences.

Table S2. HMMER prediction of the main domains in GH3 sequences.

Table S3. HMMER prediction of the main domains in GH20 sequences.

Table S4. HMMER prediction of the main domains in GH75 sequences.

Table S5. Summary of the Phyre2 analysis for the GH3 sequences of the *A. album*

Table S6. Summary of the Phyre2 analysis for the GH20 sequences of the *A. album*

Figure S1. Clustal alignment of *A. album* GH18 chitinases.

Figure S2. Clustal alignment of *A. album* GH3 sequences.

Figure S3. Clustal alignment of *A. album* GH20 sequences.

Figure S4. Clustal alignment of *A. album* GH75 sequences.

Figure S5. Expression of recombinant Chi1 mature protein.

Figure S6. SDS-PAGE (15% polyacrylamide gel) analysis of chromatographic fractions.

Figure S7. SDS-PAGE (15% polyacrylamide gel) of the purified Chi1 recombinant protein.

Table S1. HMMER prediction of the main domains in GH18 sequences.

	Id	Length	HMMER					
			LysM	C-R	GH18	CBM	Pept_M508	P-E
1	KAJ6787801.1	336			33-313			
2	KAJ6781922.1	445			30-317	415-441		
3	DAA05865	392			31-307	360-388		
4	KAJ6780968.1	757			31-312	378-405	529-732	
5	KAJ6788767.1	324			38-310			
6	KAJ6787922.1	315			30-253			
7	KAJ6780669.1	423			137-380			
8	KAJ6780036.1	330			24-312			

9	KAJ6780631.1	348			13-289			
10	KAJ6787472.1	365			42-298			
11	KAJ6779939.1	369			21-352			
12	KAJ6782108.1	363			1-335			
13	KAJ6782480.1	392			22-380			
14	KAJ6785755.1	574			31-338			
15	DAA05854	492			100-451			
16	KAJ6782844.1	540			157-501			
17	KAJ6786186.1	394			4-349			
18	KAJ6783291.1	428			42-388			
19	KAJ6787487.1	423			39-383			
20	KAJ6785998.1	1832		141-187	202-541			
21	KAJ6779834.1	532			153-493			
22	DAA05857	1533	308-355		520-848			
23	KAJ6783597.1	742			15-358			
24	KAJ6781350.1	671			86-435			
25	KAJ6786554.1	1495			496-844			1380-1481
26	KAJ6789350.1	754			152-343			
27	KAJ6787215.1	1373	403-445		606-949			
28	KAJ6784990.1	1418	349-396	411-476	490-841			
29	KAJ6784672.1	1279	295-337	420-486	501-852			

Legend (Pfam IDs are reported in brackets): LysM=LysM domain (PF01476); C-R=Chitin recognition protein (PF00187); GH18=Glycosyl hydrolases family 18 (PF00704); CBM= Fungal cellulose binding domain (PF00734); Pept_M508= Peptidase M50B-like (PF13398); P-E=Pathogen effector; putative necrosis-inducing factor (PF14856).

Table S2. HMMER prediction of the main domains in GH3 sequences

	**Id**	**Length**	**HMMER**				
			WSC	GH3-N	GH3-C	AT	F-III
1	KFG78085	909		28-357			
2	KAJ6788372.1	932		21-350			
3	AGC24356	858		8-336		552-655	
4	KAJ6789110.1	539		6-330			
5	KAJ6787877.1	370		32-364			
6	KAJ6782998.1	369		47-366			
7	KAJ6785893.1	870	32-113	169-461	499-724		
8	KAJ6786934.1	830		5-277	312-703		736-808
9	KAJ6780833.1	760		46-324	369-634		681-747
10	KAJ6787866.1	802		55-341	388-659		717-791
11	KAJ6786079.1	777		85-334	382-642		700-766
12	KAJ6785739.1	893		54-358	419-672		796-870
13	KAJ6782204.1	948		166-420	485-751		868-939
14	KFG84234	879		85-354	414-673		
15	KAJ6788989.1	883		97-356	416-677		804-873

Legend (Pfam IDs are reported in brackets): WSC=WSC domain for carbohydrate binding (PF01822); GH3-N=Glycosyl Hydrolase family 3 N-terminal domain (PF00933); GH3-C=Glycosyl Hydrolase family 3 C-terminal domain (PF01915); AT=Acetyl Transferase (GNAT) family (PF00583); F-III=Fibronectin type III-like domain (PF14310).

Table S3. HMMER prediction of the main domains in GH20 sequences

	Id	Length	HMMER			
			GH20-2	β-AH	GH20	β–AG
1	KAJ6784096.1	848	157-304		307-638	
2	KAJ6785798.1	741	32-167		170-339	
3	KAJ6780494.1	661	48-191			197-508
4	KAJ6785933.1	514	23-92		97-448	
5	KFG80340	620		27-171	193-564	
6	KAJ6783033.1	633		28-184	207-577	
7	KAJ6780076.1	577		16-163	188-528	
8	KAJ6789215.1	751		204-335	360-628	
9	KAJ6787434.1	574		20-161	186-526	
10	KFG85702	579		21-166	191-530	
11	KAJ6780083.1	579		19-165	190-529	

Legend (Pfam IDs are reported in brackets): GH20-2=Glycosyl Hydrolase family 20 domain 2 (PF02838); β-AH=beta acetyl hexoaminidase like (PF14845); GH20=Glycosyl Hydrolase family 20 catalytic domain (PF00728); β-AG=beta-N-acetylglucosaminidase (PF07555).

Table S4. HMMER prediction of the main domains in GH75 sequences

	Id	Length	HMMER	
			GH75	A-L
1	KAJ6785337.1	1093	667-836	877-1092
2	KAJ6783761.1	252	80-249	
3	KAJ6781651.1	299	82-251	
4	BAA12799	304	82-254	
5	KAJ6788818.1	300	81-253	
6	KAJ6786595.1	273	52-234	
7	ADG96019	304	100-244	
8	KAJ6781485.1	256	105-249	
9	AA041660	238	72-235	
10	KAJ6783315.1	232	70-231	
11	KAJ6787168.1	234	70-231	
12	ABY77913	217	53-214	

Legend (Pfam IDs are reported in brackets): GH75=Fungal chitosanase of glycosyl hydrolase group 75 (PF07335); A–L=Alginate Lyase (PF08787).

Table S5. Summary of the Phyre2 analysis for the GH3 sequences of *A. album*

GH3 sequence	Model	Enzyme	Cnf	Cvr	Id	Organism
KAJ6786934.1	3AC0	β-glucosidase	100	97	49	*Kluyveromyces marxianus*
KAJ6780833.1	4IID	" "	100	94	47	*Aspergillus aculeatus*
KAJ6788989.1	5NBS	" "	100	95	62	*Neurospora crassa*
KAJ6788372.1	4ZM6	β-N-acetylglucosaminidase	100	89	30	*Rhizomucor miehei* CAU432
KAJ6782204.1	4IID	β-glucosidase	100	85	46	*Aspergillus aculeatus*
KAJ6785739.1	4D0J	" "	100	91	42	*Rasamsonia emersonii*
KAJ6787866.1	" "	" "	100	91	45	" "
KAJ6785893.1	7VC7	β-xylosidase	100	84	51	*Phanerochaete chrysosporium*
KAJ6787877.1	6K5J	β-N-acetylglucosaminidase	100	88	31	*Paenibacillus* sp. str. fpu-7
KAJ6789110.1	4ZM6	" "	100	94	39	*Rhizomucor miehei* CAU432
KAJ6782998.1	6K5J	" "	100	88	31	*Paenibacillus* sp. str. fpu-7
KAJ6786079.1	4D0J	β-glucosidase	100	93	43	*Rasamsonia emersonii*

Model = PDB model; Cnf=confidence (%); Cvr=coverage (%)

Table S6. Summary of the Phyre2 analysis for the GH20 sequences of *A. album*

GH20 sequence	Model	Enzyme	Cnf	Cvr	Id	Organism
KAJ6780076.1	3NSN	β-N-acetylglucosaminidase	100	90	31	*Ostrinia furnacalis*
KAJ6780083.1	" "	" "	100	90	31	" "
KAJ6785933.1	3RCN	" "	100	93	48	*Paenarthrobacter aurescens*
KAJ6783033.1	5OAR	" "	100	79	67	*Aspergillus oryzae*
KAJ6780494.1	6PV5	GH84 Hydrolase	100	83	32	*Clostridium perfringens*
KAJ6787434.1	3NSN	β-N-acetylglucosaminidase	100	91	33	*Ostrinia furnacalis*
KAJ6784096.1	6JQF	" "	100	72	24	*Akkermansia muciniphila*
KAJ6785798.1	" "	" "	100	82	25	" "
KAJ6789215.1	3LMY	" "	100	62	30	*Homo sapiens*

Model = PDB model; Cnf = confidence (%); Cvr = coverage (%); Id = identity (%)

Figure S1

B

```
KAJ6780631.1      ------------------------------------------------------------   0
KAJ6787472.1      ------------------------------------------------------------   0
KAJ6780036.1      ------------------------------------------------------------   0
KAJ6787922.1      ------------------------------------------------------------   0
KAJ6780669.1      MNWLKESIQLPFGGRKNSEASQTGTTASSTTDITSQAGWVDPEKAEAAANMQAASAKPET   60
KAJ6788767.1      ------------------------------------------------------------   0
KAJ6787801.1      ------------------------------------------------------------   0
KAJ6781922.1      ------------------------------------------------------------   0
DAA05865.1        ------------------------------------------------------------   0
KAJ6780968.1      ------------------------------------------------------------   0

KAJ6780631.1      ------------------------------------------------------------   0
KAJ6787472.1      -----------MRWSLFSVVS-------LLAPLL------------QASPVGDVQAS---   27
KAJ6780036.1      -------------------------------------------------MVGASPLT---   8
KAJ6787922.1      -------------------MHATSLLFAANI----------------------------I   13
KAJ6780669.1      VAADDEKTTTKRQWPLWMKLAIAGCIFAVVVGLSVGLGVGLTRHKHNGSPGGDSSSSSGD   120
KAJ6788767.1      --------------MLFSTSAIA--AFAALT-----------------SLGAAAPANEGA   27
KAJ6787801.1      -----------------MR---SSATFTALVAIT-------------------------A   15
KAJ6781922.1      -----------------MR---SLS---LLAGLG-------------------------L   12
DAA05865.1        -----------------MP---SISSFTSALGLL-------------------------S   15
KAJ6780968.1      -----------------MV---SSSSVASVANLL-------------------------A   15

KAJ6780631.1      -----MPPLPIGPTPLPRLITYYQTLHDGDGK---PISILPL----ITQPSIAVTHVILA   48
KAJ6787472.1      ---AQASASTQAISELPRLIIYFQTTHDQSGR---PISMLPL----INEKGIALTHLIVC   77
KAJ6780036.1      --RRADTCATKGRPAGKVLQGYWENWDGSSNGVHPGFGWTPIQNPDIRKHGYNVINAAFP   66
KAJ6787922.1      ASFTLASR-ARPRDASKKLAVYWGANDYQ----------LTLDAVCSD-DSYNIVNLAFL   61
KAJ6780669.1      SSSGSGSS-GGSPQRPSKLAVYWGSKFNT----------IPLDDVCED-PSYDIVNLAFL   168
KAJ6788767.1      S------T-LRTRGAGGKLVVYWGAEDDN----------LSLMDVCND-PTYDIVNLAFL   69
KAJ6787801.1      SVSAAPHTITSRADSGAQNVVYWGQNG----GG--VVENNDLATYCTKSAGIDIVVLSFL   69
KAJ6781922.1      TSLAAASPLAARQAPGARNVVYWGQNG----GG--TIENNDLSAYCQSNSGIDVIVLAFL   66
DAA05865.1        LLPA--ARAGWNQNSNDNIVVYWGQNS-----G--SVGQNRLSYYCQNAPDVDVINISFM   66
KAJ6780968.1      MLPV--AMAAFNPGSNKNIAVYWGQNSYNQGSG--PLAQQRLGTYCQNT-DLSIIPLAFM   70
                                       *:                  :           :    .

KAJ6780631.1      AIHINEDPDG-----LTLND-----HSPDHERHQTAWAELR-----VLQAA-GIRVMVLL   92
KAJ6787472.1      SLHINKDS-E-----IHLND-----YPPGDPHFYTLWNETA-----HMKKA-GVKVMGMI   120
KAJ6780036.1      VI-YPDGTA---LWEDGMD---------------TGVKVASPSDMCAAKAA-GATILMSI   106
KAJ6787922.1      SH-FFSAGGYPTLFLSPLNTASEAQAARGATDLRDGTSLIPALQKC--RDS-GKKILLAM   117
KAJ6780669.1      SY-FFGNGQYPRLAIPSLNGSSEAQRLAGAVDLQDGTALVPAIKKC--QAN-GKLVLLSM   224
KAJ6788767.1      AY-FNKAGGYPGLEMSGLDGPSAAQRKAGATGLKDGSPLVPAIKKC--QAN-GKLVILSM   125
KAJ6787801.1      YK-YGA-GQKIASGVIGQSCS-----IGDSGEGQNCDDLTKAIDTC--KSN-GVKVIMSL   119
KAJ6781922.1      YQ-FGRDGGTIPSGTIGQSCF-----ISNSGEGQNCDALNAAIAKC--QGA-GVKMILSL   117
DAA05865.1        VG-ITNL--NLNLANVGNNCT----AFPQAPNLLNCPQVAADIIEC--QQTYGKTIMMSL   117
KAJ6780968.1      NG-ITPA--ITNFANAGDNCT----AFPDNGNVLSCPQIEEDIKSC--QSKYGKTISLSL   121
                                    .                             :   *  :   :

KAJ6780631.1      GGAAKGTYQRLDGSPDDESPAELARFERYYA------PLRDLLRARGLDGIDLDVEEEMS   146
KAJ6787472.1      GGAAPGSFDSSSLD------GDQETFEKYYG------QVKGVITKFGLEGMDLDVEQPMS   168
KAJ6780036.1      GGATAAI--DLSSSAVADKFV-S--------------TIVPILKKYNFDGIDIDIESGLT   149
KAJ6787922.1      GGAPGYSDVRLENDDQGRQIA-NTLWNLFGG--GTEDAELRPFGAFKLDGFDIDNESGDA   174
KAJ6780669.1      GGAAGYSDVSVGSDGTGQLVA-DLIWNLFLG--GGKKPEIRPFGNVVLDGVDFDNESGQS   281
KAJ6788767.1      GGATDYSNVRLSNDAAGEKVA-DQVWNLFLG--GTDHKELRPFGDVKLDGVDLDNETNDA   182
KAJ6787801.1      GGAS-GA-YSLSSKEEAEAIG-QNLWEAYGNLNGGNGSVPRPFGKTFVNGWDFDIESNSG   176
KAJ6781922.1      GGAS-SS-YALSSQSQAEQIG-QYLWDSYGN--SGNTTVKRPLGNNVVDGFDFDIELNGG   172
DAA05865.1        FGST-YSESGFSSSSAAVAAA-QEIWAMYGPVQSGN-STPRPFGDAVVDGFDFDLEDPIE   174
KAJ6780968.1      GGAT-YSQGGWSSSSAAESAA-QMVWDMFGPVQSGK-NVDRPFGSAVVDGFDFDFESS-A   177
                   *:          .                             :    .:*  *:*  *

KAJ6780631.1      -LAGIVRLI-------DRLRADF----GPQFIITLAPVAASLLDPTANLS---GFDYEAL   191
KAJ6787472.1      -QSGITRLI-------ERLRQDF----GSGFIITLAPVASAL-RNSANLS---GFNYKTL   212
KAJ6780036.1      GSGDIKTLSTSQSNLIRVIDGILA-QMPSNFGLTMAPETAYVTGGSVTYGSIWGSYLPII   208
KAJ6787922.1      --TGYMAMI-------DQFRSNFATDTSREYTLSAAPGCGFPGD--------------SI   211
```

```
KAJ6780669.1      --TGYEAMA-------QHFRSHFASDPSKQYYMTAAPQCPFTAD--------------DE      318
KAJ6788767.1      --TGYLAMT-------KRFRANMAKDASKKYYLTAAPQCPFPDQ--------------SE      219
KAJ6787801.1      -NQFYPDMI-------AKLRGNFASDSSNQYFITGAPQCPIPEP--------------NM      214
KAJ6781922.1      SSQYYQYMI-------AKLRQNFAKDSSKKYYIAGAPQCPIPEP--------------NM      211
DAA05865.1        --NNMEPFA-------AELRSLIDASTSKKFYLSAAPQCPYPDV--------------SD      211
KAJ6780968.1      --NNLPAFG-------AKLRSLMDSAGGKKYYLSAAPQCVFPDA--------------AV      214
                         :          :   :       : :: **

KAJ6780631.1      EVMR--GREVAWYNTQFYCGWGDFSST-------VMYDMMLRKGWAP-------------      229
KAJ6787472.1      NTAV--GSKISFYNGQFYSGFGTMART-------DDYVKIVDNGFAA-------------      250
KAJ6780036.1      KKYVD-NGRLWWLNMQYYNGDMYGCSG-------DSYKAGTTQGFIAQTDCLNNGLTIGG      260
KAJ6787922.1      TPEI--AGAVDHVWIQFYDTKYCLIGD-------DTFYG-KVRTWSQ-------------      248
KAJ6780669.1      ELSL--FQHLDYVSVQFYNNNVCNVGQ-------SGFET-SVRRWSE-------------      355
KAJ6788767.1      PLSV--CKLLDYVWVQFYNNGDCNIAQ-------SGFNA-AVKNWSK-------------      256
KAJ6787801.1      NEIIT-KAHFDYLFVQFYNNPGCSVD--------GTINFEDWKKNVANT-----------      254
KAJ6781922.1      GEIIS-TSQFDWLFVQFYNNNNYTVPCALPINGNAPFNYNNWTSFISTT-----------      259
DAA05865.1        ESFLDGQVPFDWVNVQFYNNGCGVSHYP------ADFNWATWDNWAKTV-----------      254
KAJ6780968.1      GAALD-AVAFDFVMIQFYNNWCGVSNFQEGASSQNAFNFDVWDKWAKGS-----------      262
                           .     *:*

KAJ6780631.1      -------EKVVVGLVTNPAN-------GPGFIAWDSLQQTLAIL-RQRPHNFGGIMGWEY      274
KAJ6787472.1      -------SKVVAGQLTDPSN-------GGGYIPYSQLNATIIAL-RKKYGQIGGVMGWEY      295
KAJ6780036.1      VTIKVPYDKQVPGLPAQPGA-------GGGYMSTSDVSSVLNHY----NGNLKGLMTWSL      309
KAJ6787922.1      ---AVKG-DIYIGATARNTP------DQVGYLEPHALLTALHKVEAMGLPNYSGAMLWEA      298
KAJ6780669.1      ---AIGNTTLLVGALASNAD------KDEGYVDAKTFNSVLGKVKAMNLPNYGGVMLWEA      406
KAJ6788767.1      ---GIGNAKLFIGGLASGSD------GDQGYVDSNTFIKAIKGVEAMNLPNYGGAMLWEA      307
KAJ6787801.1      ---PSSSAKIFIGVPASPLGATGTESGAKYYLEPTKLASLVGEH--KSDAAFGGVMMWAA      309
KAJ6781922.1      ---PSKDAKIFIGVPASTLASNGSPNGATYYATPEQLATIVNDY--KSDAHFGGIMMWSA      314
DAA05865.1        --SANKNAKVLIGTPANVGGANAG-----SFPTDSQLSGAISLA--KGSSSFGGVMLWDM      305
KAJ6780968.1      ---KNPNVKLLLGIPAAPGA--GG-----GYTSGSKLKAAINYS--QKYSSFGGVMLWDM      310
                              *  :              :     .   :            * * *

KAJ6780631.1      YNSLPGDRERPWEWARTMTAMI---RSH-CLAPAPPVATP-PRPEEKP-----AAVVDID      324
KAJ6787472.1      FNSQPGGTKEPWKWAQVMTQIL---RPN-AVPKLSITTAD-AD---H-------LTNTYE      340
KAJ6780036.1      NWDGSKNWSFGDN-------VKGTLGTA--------------------------------      330
KAJ6787922.1      ELAMKN-KNYQKT-------LRAGL-----------------------------------      315
KAJ6780669.1      ELAAKN-GNYQKK-------IKAEL-----------------------------------      423
KAJ6788767.1      QLSRNN-GNFAKK-------IRSSL-----------------------------------      324
KAJ6787801.1      GFSDAN-VNNGCTYAQEAKHILTTGSAC--------------------------------      336
KAJ6781922.1      GFSDSN-VNNGCTYAQEAKHILVNGGPCGSNGPPPPTSTPPPTSTAPPTGTPPSTSTTST      373
DAA05865.1        AQLFT---NQGYL--A---KIVADLGSASSPPPPASTTLQ----------------TIT      340
KAJ6780968.1      SQLYS---NNGFL--G---EVLSDISGGPATTQPPSTTTQPPGTTTQPPGTT----TTCT      358
                          .           :

KAJ6780631.1      ADA---PGGEELEIP--APFEYYSDGAEE-------------------------------      348
KAJ6787472.1      HTV---SGGSPNARPASPGINYYDMVNA--------------------------------      365
KAJ6780036.1      ------------------------------------------------------------      330
KAJ6787922.1      ------------------------------------------------------------      315
KAJ6780669.1      ------------------------------------------------------------      423
KAJ6788767.1      ------------------------------------------------------------      324
KAJ6787801.1      ------------------------------------------------------------      336
KAJ6781922.1      HPCASHTSSSPPVSSPRGTVPQWGQVRRNEERRDGEFDANIKKCGGKGYNGPTECVPPFT      433
DAA05865.1        RSS--TASTGPTNPPSGGSVPQWG------------------QCGGNGYTGPTQCQAPYK      380
KAJ6780968.1      STT--TPASTSTPPPTSGGLDQYA------------------QCGGQGYSGPTVCKAPYT      398


KAJ6780631.1      ------------------------------------------------------------      348
KAJ6787472.1      ------------------------------------------------------------      365
KAJ6780036.1      ------------------------------------------------------------      330
KAJ6787922.1      ------------------------------------------------------------      315
KAJ6780669.1      ------------------------------------------------------------      423
KAJ6788767.1      ------------------------------------------------------------      324
KAJ6787801.1      ------------------------------------------------------------      336
KAJ6781922.1      CVKLGEWWSSCR------------------------------------------------      445
DAA05865.1        CVATSEWWSSCQ------------------------------------------------      392
KAJ6780968.1      CVKQNTLPSRLTSATSSHLHLAQATTLLQLLPFIALRSVAFTFCDPDPRVHSSPGSLTRA      458


KAJ6780631.1      ------------------------------------------------------------      348
```

```
KAJ6787472.1        ------------------------------------------------------------    365
KAJ6780036.1        ------------------------------------------------------------    330
KAJ6787922.1        ------------------------------------------------------------    315
KAJ6780669.1        ------------------------------------------------------------    423
KAJ6788767.1        ------------------------------------------------------------    324
KAJ6787801.1        ------------------------------------------------------------    336
KAJ6781922.1        ------------------------------------------------------------    445
DAA05865.1          ------------------------------------------------------------    392
KAJ6780968.1        AYYQLAKTALLRRFEASYIMAPSFSTSSGILETHNPVRIFGQKPEPSPGLSLASRSLQHP    518

KAJ6780631.1        ------------------------------------------------------------    348
KAJ6787472.1        ------------------------------------------------------------    365
KAJ6780036.1        ------------------------------------------------------------    330
KAJ6787922.1        ------------------------------------------------------------    315
KAJ6780669.1        ------------------------------------------------------------    423
KAJ6788767.1        ------------------------------------------------------------    324
KAJ6787801.1        ------------------------------------------------------------    336
KAJ6781922.1        ------------------------------------------------------------    445
DAA05865.1          ------------------------------------------------------------    392
KAJ6780968.1        NHTQQVTLGVIAAYVVVIAILWNVPYIKNILWPFKMLVIAFHEFGHAITAVFTGGRVKSI    578

KAJ6780631.1        ------------------------------------------------------------    348
KAJ6787472.1        ------------------------------------------------------------    365
KAJ6780036.1        ------------------------------------------------------------    330
KAJ6787922.1        ------------------------------------------------------------    315
KAJ6780669.1        ------------------------------------------------------------    423
KAJ6788767.1        ------------------------------------------------------------    324
KAJ6787801.1        ------------------------------------------------------------    336
KAJ6781922.1        ------------------------------------------------------------    445
DAA05865.1          ------------------------------------------------------------    392
KAJ6780968.1        SLDPNEGGVTKMVGGISAITLPAGYLGSSIIGALLTFCGFNIVASKIASIVLGVCFLLTL    638

KAJ6780631.1        ------------------------------------------------------------    348
KAJ6787472.1        ------------------------------------------------------------    365
KAJ6780036.1        ------------------------------------------------------------    330
KAJ6787922.1        ------------------------------------------------------------    315
KAJ6780669.1        ------------------------------------------------------------    423
KAJ6788767.1        ------------------------------------------------------------    324
KAJ6787801.1        ------------------------------------------------------------    336
KAJ6781922.1        ------------------------------------------------------------    445
DAA05865.1          ------------------------------------------------------------    392
KAJ6780968.1        WWGKRDWLTVLTVLLAVALLVACWFIKHAEALRFVVLFIGVMSSLYSVWDICDDLILRKV    698

KAJ6780631.1        -----------------------------------------------------------     348
KAJ6787472.1        -----------------------------------------------------------     365
KAJ6780036.1        -----------------------------------------------------------     330
KAJ6787922.1        -----------------------------------------------------------     315
KAJ6780669.1        -----------------------------------------------------------     423
KAJ6788767.1        -----------------------------------------------------------     324
KAJ6787801.1        -----------------------------------------------------------     336
KAJ6781922.1        -----------------------------------------------------------     445
DAA05865.1          -----------------------------------------------------------     392
KAJ6780968.1        NSSDASVFAKRYGGSSQCWGIIWSIISVLIMAVGIVAGLAAFSQSFAQQQEDSKHFIPT     757
```

A

```
KAJ6779939.1        ------------------------------------------------------------     0
KAJ6782108.1        ------------------------------------------------------------     0
KAJ6782480.1        ------------------------------------------------------------     0
KAJ6785755.1        ------------------------------------------------------------     0
KAJ6786186.1        ------------------------------------------------------------     0
KAJ6783291.1        ------------------------------------------------------------     0
KAJ6787487.1        ------------------------------------------------------------     0
DAA05854.1          ------MFRLLCIGLPLL---AR-------VAVAVPATLSERCDAPNP---PASSLT---     38
KAJ6782844.1        MLQSAIILPLLALGVRGAPAGSGDGVSAACRVVWVPVSVDTTVQAPSATNTATSPLTDGR     60

KAJ6779939.1        ------------------------------------------------------------     0
KAJ6782108.1        ------------------------------------------------------------     0
KAJ6782480.1        ------------------------------------------------------------     0
KAJ6785755.1        ------------------------------------MD----------------------     2
KAJ6786186.1        ------------------------------------------------------------     0
KAJ6783291.1        ------------------------------------MLKLLKNSVAVTAA----------     14
KAJ6787487.1        ------------------------------------MLSFVKKSIALVAA----------     14
DAA05854.1          ------------------------ESVAAISSGPLPFDTEMGDVETVHRE--PFDAVPVG     72
KAJ6782844.1        AGAAYSSGRENKFVLPQGASSTKPDTTAAIQSTSTTMDRPSKVTSAVTRTASSNKLSPTG     120

KAJ6779939.1        ---------------------MKTSPLTLLAAASTAAAAAPRNVMYFDQWHTAT---LPP     36
KAJ6782108.1        --------------------------------------------MYLTGQHNVI---PED     13
KAJ6782480.1        ----------------MHLGSYIS----AVLAFTTTGVLGKNIVGYYPSWKRQY----AD     36
KAJ6785755.1        -------------SSSVASASLPSTPTLQTRPFPELGQVMYMNAVYFHNSHI-ARDETPA     48
KAJ6786186.1        --------------------------------------MSYRSVAYYVNWAIYARKHLPQ     22
KAJ6783291.1        --------LYAFTA----LATLIT-----SDSTIGKRESGYVNAVYYTNWAIYLRNFQPS     57
KAJ6787487.1        --------LQAVTA----LATPIS-----SEAGVEKRGSGFANAVYFTNWGIYGRNFQPA     57
DAA05854.1          PEPA---------SSGVSIEHGHG-----SEVVGFGPTSRKKNVVYYADWSIYDAAFLPQ     118
KAJ6782844.1        TSTSTITVTKTITRSGQKPAYTPG-----TDQDDGVEIKGRKNVVYFTNWSGYGANYTAA     175
                                                                 *

KAJ6779939.1        KDVTAGVTHVITAFAP----DGTFNWGS---SYTPFMSL---------------------     68
KAJ6782108.1        VQLRKEITHVVLAFVR----SEMFNVESPHPDYPLFTSV---------------------     48
KAJ6782480.1        NMDLSLYTHINFAFAIPAQ-DGSFSFDGDWFL----------------------PG----     69
KAJ6785755.1        KLDYGFINLVYYAYASVAP-DGIVNLGDEWADIRAPVDG--------------VEGGLGS     93
KAJ6786186.1        DLPAENITHVLYSFANVRSENGEVFMTDGWADTDIHWEGDS-----WNDSGTNIYGCLKQ     77
KAJ6783291.1        DLIVSDITHILYSFMNLRT-DGVVYSGDTYADYEKHYPGDS-----WTEGVKNAYGCVKQ     111
KAJ6787487.1        DLPASEITHVLYSFMNVRA-DGTIFSGDTYADYEKHYAGDS-----WNDVGTNAYGCVKQ     111
DAA05854.1          QLPAEDITHLLYAFAGIED-DGSVVSMDPWADEEKMLSVPGGPGRNDTQGLNDVHGAVEQ     177
KAJ6782844.1        EFPANDVTHLLYAFGDISS-TGEVIPSDSWIDIQKQFAGPQ---KRDV----QAQGNIGQ     227
                           . :  ::         .   .

KAJ6779939.1        SDIRALFDQGTKVCMAIGGWGDTT--GFGTAAASDESRKRYAKNVAEALNTLGYDCVDVD     126
KAJ6782108.1        KEVREKFPPTTKILVAIGGWGNSL--GFEEAALDDASRKRWTRNVKHMVETTGADGVDID     106
KAJ6782480.1        -VVSKLHDKGVKALMSVGGWTGSN--YFSTILKDQTAGANLVANIVSYVKANSLDGIDID     126
KAJ6785755.1        LMQLKQRYRHLKVLLSIGGPAAS--GVFVSIASNPATRNNFAYTAAGLVVASGLDGVDIS     151
KAJ6786186.1        LNILKKRNRNFKVMLSIGGWTYSS--NFPAPASTPEGRDTFAKSCVELVKNLGFDGIDID     135
KAJ6783291.1        LYLLKKQNRNVKVILSIGGWNWRA--NFPAASSSAETRKTFAQSAVELMKDWGFDGIDID     169
KAJ6787487.1        LYLLKKQNRNMKVMLSIGGWTWST--NFPAAASSAATRKTFAQSAVGFMKDWGFDGIDID     169
DAA05854.1          VFLLKKRHRHMKTLLSIGGWNMSQSGKFAPVLNTNEGRIRFAKTAVNLLANWGLDGIDID     237
KAJ6782844.1        IFGLKKQNRNLKVLISVGGFTYSEAGKFVPATNTTEGRQRFARSAVKLIANWGFDGIDID     287
                               *  :::**        *             . .    :   . * :*:.

KAJ6779939.1        WEYPGGNGEDYKQNPNSGKTAEIETYPLLLKEIKAVIGD---------K-ELSIAVPGKV     176
KAJ6782108.1        WEYPGGNRDDYKVITNDQREWEIDAYHLLLKELRETIGR---------NKIISIAVPGLE     157
KAJ6782480.1        WEYPGRLGNNCNAYD---PQSDAQNFLAFLQTLRQKLDAEFGSRK----KLLTLAMRVQP     179
KAJ6785755.1        WHVPAD-------------PVQGANFLALAQAVRSKLQG---------GRMLTAALPAN-     188
KAJ6786186.1        WEYPKS-------------AEEAGHFVELLAEVRKQLDEYSARAADNYHFELSVACPAG-     181
KAJ6783291.1        WEYPSN-------------DTEAQNMVLLLQAVREELDSYAAHYADGHHFLLSIAAPAG-     215
KAJ6787487.1        WEYPAD-------------ATQAQNMVLLLQAVRSELDSYAAQYAKGHHFLLSIAAPAG-     215
DAA05854.1          YEYPIN-------------EREAVGFVDLLRECRKALDEYASLHNQRYHYLLTAAVSAA-     283
KAJ6782844.1        WEFPKD-------------KSEAANFVSLLQEIRYELDKYASDNNQKYHYLLTVATSAG-     333
                    :. *                 :      :    :  :              :: *
```

```
KAJ6779939.1        GDMIAFTAEQVPKISETVDFVNVMTYDLMNRRDTSTDHHSSVS-----------GSLATV     225
KAJ6782108.1        RDLMAFTANTVPKIVKQVDFINIMTYDLINRRDTVVQHHSGVA-----------DSRASI     206
KAJ6782480.1        FDGPNGPLSDVSAFAKVVDYGNLMQYDINGGWNSVTGPLAPFNFESGRGA--QVSFVSAI     237
KAJ6785755.1        --PAVLQHIDLAVAAESLDFINLQTYNLHHAQPGRSLCPSQLYSMAKDEP----STSTVA     242
KAJ6786186.1        --PDNFQKLNIPGMDAYLDFWNLMAYDYAGSWDQVSGHQANLYPCNDNPACTPFSTTAAV     239
KAJ6783291.1        --PEKYKKLRLADLGKVLDYINLMAYDFAGSWSNYTTHNANLYANPQNPNATPFNTEDAV     273
KAJ6787487.1        --PDNYNKLKFAELGKVLDYINLMAYDYAGSWSNYTGHDANIYANPQNPNATPYNTDDAV     273
DAA05854.1          --QQHYKWLDMPAIDRYLDAWHLMAYDYAGSWDDLSGDQSNVFHDATNPHRTRANSDQAV     341
KAJ6782844.1        --PSKYKLLNLGAMDKFVDSWHLMAYDYAGSWDSTTGHQANVYPDPSNTASTKFNTEQAV     391
                              .      :*  ::  *:            : .            .

KAJ6779939.1        ETYLSRGMPAAKMNLGFAFYAKWFTTKDGATCDQATGCPTAVLEDANGGD-TGK------     278
KAJ6782108.1        QRYLDRGVPRSMANLGLGYYVKWVMTDP-CERDDILGCPTQLLEDPDSGNDLGR------     259
KAJ6782480.1        DAWTAAGWPAAQLNAGYAFYGLSTTALEDMTKDPT-----NQYQPQSSTVPLGDKEDASW     292
KAJ6785755.1        GYILSRNFPAQKLLLGIPTFGTSFPGCDGPGQ---------AYDPAE-------------     280
KAJ6786186.1        DYYTQNGVAPDKLVLGMPLYGRAFENCDGIGK---------PFQGVG-------------     277
KAJ6783291.1        KAYVSEGIPANKIVLGMPIYGRSFQQTEGIGK---------PYNGIGE---------A--     313
KAJ6787487.1        QAYINGGVPANKIVLGMPIYGRSFQQTEGIGK---------PYNGIG-------------     311
DAA05854.1          NDYVAAGVNPEKIILGIPLYGRSFMNTDGPGK---------PYEGVG-------------     379
KAJ6782844.1        NDYIAAGIAPEKIILGLPLYGRSFMQTSGMGQ---------SFNGTG-------------     429
                          .        *   :                       :

KAJ6779939.1        ----------SGAVTFEKE----------NYSGAFLNAMNNGKADNEKGGQWYWDASAKV     318
KAJ6782108.1        ----------TAAFSWHDN-------VPSELSASFSRALNRGR-YFEDGSYGYWDEQEHR     301
KAJ6782480.1        YDACAGATSNSGTWQWKHLLDQGVLSAPTTASAPWV-----RQWDNTTQTPWLFNPQTSI     347
KAJ6785755.1        ----------SQSMDYLGLPVAGRQ----------------EGIDRRRISAYCAGG-PEG     313
KAJ6786186.1        -----QGTWEQGVYDYKKLPLEGAE----------------EHQDDASGATYCYNPQSRT     316
KAJ6783291.1        --SFGQGSWENGVWDYKALPRAGAT----------------VTCDDTAKGCYSYNANTQE     355
KAJ6787487.1        -----SGSWENGIWDYKALPKAGAT----------------VKCDDTAKGCYSYDPSTKE     350
DAA05854.1          -----KGSIEKGVWLYRDLPRPGSV----------------VNLNRDIIAAYSYDNATRE     418
KAJ6782844.1        -----EGGVEAGVWLYKDLPRPGAT----------------VTVDSDVLAAYSYDPKERM     468
                               :                                      .

KAJ6779939.1        YWTWDTPEFIAKKMTDIVKAKGLGGVMA----------------WSLAQDSHDWSH-IKA     361
KAJ6782108.1        WWSFDTPDVIARKMKELVGSMGLGGVFA----------------WGLGEDATDFAR-LAA     344
KAJ6782480.1        FISYEDPVSLKVKV-DYAASKGLAGAMV----------------WSVNMDDSSN-TLLKV     389
KAJ6785755.1        FIAYDNPDTVKEKA-DFCKQKGFGWTTPKGASDDATPEHWPRCVWGASARSSR-CSLACV     371
KAJ6786186.1        LVTYDTPQLAKTKA-QFIKERGLGGGMW----------------WESSADKAGGDSLIQT     359
KAJ6783291.1        LISFDTPSMISTKV-EWLKGNGLGGSMF----------------WEASADKKGSDSLIGT     398
KAJ6787487.1        LISFDTPAMISTKV-SWLKGKGLGGSMF----------------WEASADKKGSDSLIST     393
DAA05854.1          LVTYDNMETTTLKA-EYLGKKGLGGAVF----------------WEASGDRSGDESLIKA     461
KAJ6782844.1        LITYDNKESANLKA-QYLMKKGLGGAVF----------------WDASGDKPGNGSLIST     511
                      :::       *  .     *:.                    *              .

KAJ6779939.1        LQS---GIKS--L-----------------------------------------------     369
KAJ6782108.1        TVD---GIRA--LHNSRDAGKDE----------L--------------------------     363
KAJ6782480.1        LRG---------------------------------------------------------     392
KAJ6785755.1        VTARADKIAAPDLASQQAMATSSADADAASRTSLSPPRNPTAGATETTDTQKRRGVVQQL     431
KAJ6786186.1        VVGEFGGPGA--LKQQENC--------------ISYPETKYDNL--------RNGLPDN-     394
KAJ6783291.1        SRQ---GLGS--LDGTQNY--------------LHYPNSTYDNI--------KKGMN---     428
KAJ6787487.1        SHQ---GLGS--QDSTQNY--------------LDYPNSKYDNI--------KKGMN---     423
DAA05854.1          VAR---KMAL--LDISMNM--------------LSYPQSQFSNI--------RNATRW--     492
KAJ6782844.1        LAR---SIWP--LESSQNM--------------LEYPKSEYANI--------RANS----     540

KAJ6779939.1        ------------------------------------------------------------     369
KAJ6782108.1        ------------------------------------------------------------     363
KAJ6782480.1        ------------------------------------------------------------     392
KAJ6785755.1        SAADCSVGDDSGAVTSSIAFGGSVPVAAAASAEHISSSETEQQPPPPPPFEPLFALLTNT     491
KAJ6786186.1        ------------------------------------------------------------     394
KAJ6783291.1        ------------------------------------------------------------     428
KAJ6787487.1        ------------------------------------------------------------     423
DAA05854.1          ------------------------------------------------------------     492
KAJ6782844.1        ------------------------------------------------------------     540

KAJ6779939.1        ------------------------------------------------------------     369
KAJ6782108.1        ------------------------------------------------------------     363
```

```
KAJ6782480.1        ------------------------------------------------------------    392
KAJ6785755.1        TTGATVHPRIHYIFSDDDTSIPTDTAAHQHGDADEHRSLVVALQPPDDHNDGAESNEIHD    551
KAJ6786186.1        ------------------------------------------------------------    394
KAJ6783291.1        ------------------------------------------------------------    428
KAJ6787487.1        ------------------------------------------------------------    423
DAA05854.1          ------------------------------------------------------------    492
KAJ6782844.1        ------------------------------------------------------------    540

KAJ6779939.1        -----------------------        369
KAJ6782108.1        -----------------------        363
KAJ6782480.1        -----------------------        392
KAJ6785755.1        PGALRSGSASQSQDTTIPTTSAA        574
KAJ6786186.1        -----------------------        394
KAJ6783291.1        -----------------------        428
KAJ6787487.1        -----------------------        423
DAA05854.1          -----------------------        492
KAJ6782844.1        -----------------------        540
```

C

```
KAJ6785998.1        ------------------------------------------------------------    0
KAJ6779834.1        ------------------------------------------------------------    0
KAJ6783597.1        ------------------------------------------------------------    0
KAJ6789350.1        ------------------------------------------------------------    0
DAA05857.1          ------------------MPWISPGGIACWLASATAIVSF----H--G--VSATANEVDA    34
KAJ6784990.1        MQ--------------------------------YHTSAALTALAGLGLFPLASGITT-S    27
KAJ6787215.1        MRLTIFTGATLLASTAHVSATVQSGLRPVSLAARMSASDFA-NVDTGGDDELAAWLKASA    59
KAJ6784672.1        MRR-----------------------TPSQVAA-----------AVSLLASVATAVTESY    26
KAJ6781350.1        ------------------------------------------------------------    0
KAJ6786554.1        --M-----------------------APSWFNAALGATSVLLSTNALVAASSPSRIGASI    35

KAJ6785998.1        ------------------------------------------------------------    0
KAJ6779834.1        ------------------------------------------------------------    0
KAJ6783597.1        ------------------------------------------------------------    0
KAJ6789350.1        ------------------------------------------------------------    0
DAA05857.1          FQSQLNLCPLACSDKS-----PEQWSVYSSFERLSQCDQPVLFDLAIHTPINDKTNSVLI    89
KAJ6784990.1        FGDYADLCPVACTSN------PSQWTKYHSLDRLAGCEADLLFDFNTLLKQVDSN--TVF    79
KAJ6787215.1        QSAIVKACPEACSELKDGEDGTTGLSLIPDENMLSRCNETMLVDLVVKSK-----DSKAI    114
KAJ6784672.1        QGQYPDDCPQLCEVAGPARGN---WTHVHHLKDLSSCKNDMIFDFNVHNLVDDPNTILTI    83
KAJ6781350.1        ------------------------------------------------------------    0
KAJ6786554.1        SYRGSERCPRSCADAGADSFA---WDSFRSMDEVDQCGNPMLFNFALADRVDEHGRNHRI    92

KAJ6785998.1        ------------------------------------------------------------    0
KAJ6779834.1        ------------------------------------------------------------    0
KAJ6783597.1        ------------------------------------------------------------    0
KAJ6789350.1        ------------------------------------------------------------    0
DAA05857.1          RACTTSPAAGGSQPMASTVK---------------REAEACGGSFVESKVPLLISQNPGQ    134
KAJ6784990.1        RTCSLASDAKPPTELAELTSNNK-----------LSVDSDCGATVKQQNVTIATSSAGAV    128
KAJ6787215.1        RACMGDYGENVKAAFKADSSKAALCDTPNYNLTTSTMSTFSG-------F-PQSIQGTSE    166
KAJ6784672.1        RACTTISNETYD-----TRSQFIRRDEGSVQSSLV-ISDSCGAKTVKSPLTPRVGNGL--    135
KAJ6781350.1        ------------------------------------------------------------    0
KAJ6786554.1        YACTTSSESFKK-----ISQAQVLAAEP--------------ADEVDAE--FEVGRGD--    129

KAJ6785998.1        ------------------------------------------------------------    0
KAJ6779834.1        ------------------------------------------------------------    0
KAJ6783597.1        ------------------------------------------------------------    0
KAJ6789350.1        ------------------------------------------------------------    0
DAA05857.1          ATAPFGNGLVTILEGLKEYRQKAVATSGCTGDNNIMLGYFDGAVVGTYAGKALSSATIPS    194
KAJ6784990.1        QDQSASTIVSSDIETAAKNLATYLKYH-ASCGSSTLFSKVNNAVVGFYSGADVQQASAAA    187
KAJ6787215.1        GTSEQSFDAKDAISALQQVSRHLQTEEPSCTDNVIKFGYSKSAAVGIFVGSEHHQHGLTK    226
KAJ6784672.1        MNRSNSTLSTTDVASAAARLQSYINKG-SQCGTTILFAKSGSAVVGLHAGSHITKSSVSE    194
KAJ6781350.1        ------------------------------------------------------------    0
```

```
KAJ6786554.1        --EDGGIAPEVAVSSLTGFLGQYVRQGLAQNGTTMIYGSFGGAAAGLYVGHGLNGGAVSA    187


KAJ6785998.1        ------------------------------------------------------------    0
KAJ6779834.1        ------------------------------------------------------------    0
KAJ6783597.1        ------------------------------------------------------------    0
KAJ6789350.1        ------------------------------------------------------------    0
DAA05857.1          I-VDYLLEEDKEQGSAPFGMLAQICDHDS-DMDHTAGVAVALKADIG-SVQSALLSWNQG    251
KAJ6784990.1        M-----LE-RFGTSLSSGPTILQTCLNSDIGADTTVGVVSGNLQELGGSLHSSVRAWVNG    241
KAJ6787215.1        TVIDEVIKSLEDKG-TSETTGFELCHPDRLGADYSAGIILTSAGNLY-KAKDVLATWASG    284
KAJ6784672.1        -----LLDT-FDAQLGQNRLSLEVCGANV-T-SSTFGIAAAEFGSLG-AVQAAVKGWADG    245
KAJ6781350.1        ------------------------------------------------------------    0
KAJ6786554.1        TALQHLEDSVKHANSSQGILGVQMCGSDY-DSDHTFGFIATTNGTFG-AIQDAMLSWYGG    245


KAJ6785998.1        ------MGSWSRIFLTTLITPSLGAV--ITSW-------PRSVDAPELPGAGTCRAIVSP    45
KAJ6779834.1        ----------MRLFRTALLAL-------LAT---------------E--------AAASS    20
KAJ6783597.1        ------------------------------------------------------------    0
KAJ6789350.1        ------------------------------------------------------------    0
DAA05857.1          ECAKAQSGTTLRELVISQVPLPS-INAKASNISLAEAPGRKAQALDSKVDGKYCKMRTI-    309
KAJ6784990.1        TCVEGSGTASMPHMLSVLVSSTTT--------------SSNSTTPVAANRRGECRDTQV-    286
KAJ6787215.1        ACVSSQN-SWMEVTV--RLPPPAKLETNTTSNSTASSSNLQSRQLKTLLPRGDCKTTTV-    340
KAJ6784672.1        ECLGDGT-ALPRMDLSVLVSTIDK---NATFY---LNGTLA-SRSLKHLPRGDCRTEQV-    296
KAJ6781350.1        ------------------------------------------------------------    0
KAJ6786554.1        DCVKFGT-SDKVKSKAHFASPLM-------SS---IQGSNSTNSTAHFSKRGECRTVQV-    293


KAJ6785998.1        FDYSAKNRRDDELPVCSADNLTLDNGISSIGQSELSLFGR--------------------    85
KAJ6779834.1        FIEQAIA-SGQLKPSNSHPNS----------KSDASL--A--------------------    47
KAJ6783597.1        ------------------------------------------------------------    0
KAJ6789350.1        ------------------------------------------------------------    0
DAA05857.1          ------------AAGDSCASIAKACKVSV---ADFFKYNGVKG-----------------    337
KAJ6784990.1        ------------HTGDSCGSLATRCGISG---PDFEKYNSGV------------------    313
KAJ6787215.1        ------------DKGMGCYDLAKKCGISS---NDLTKYNPQKNFCSTLVVDQKVCCSSGT    385
KAJ6784672.1        ------------QEHDSCAAVASRCGISV---DDLQKYNGGA------------------    323
KAJ6781350.1        ------------------------------------------------------------    0
KAJ6786554.1        ------------KQGTGCPELAKMCGISG---PDFTKYNPGK------------------    320


KAJ6785998.1        ---------------------------------------QVVGNTDYTCGPDRPCKNKAC    106
KAJ6779834.1        ---------------------------------------ATGDSPDYHCRKDKPCALGCC    68
KAJ6783597.1        ------------------------------------------------------------    0
KAJ6789350.1        ------------------------------------------------------------    0
DAA05857.1          -------------------------------------------NGNDWCRKLQAGRNICC    354
KAJ6784990.1        ---------------------------------------------KNLCSTLKDKQYVCC    328
KAJ6787215.1        LPATIPPAGGNGLCKTKEVVAHDTCGSLAAKCGLTPQDFSKINTKTGLCSSLAPGQNVCC    445
KAJ6784672.1        ---------------------------------------------SDWCNKLMPKQHVCC    338
KAJ6781350.1        ------------------------------------------------------------    0
KAJ6786554.1        ----------------------------------------------TFCNDLMPGQHVCC    334


KAJ6785998.1        CPKK---TGQCNYGEEACGTSGISPNDVCWSN------------------------CDA-    138
KAJ6779834.1        GPLDDEGNGICGMGPKFCG-------KGCTSS------------------------CDQ-    96
KAJ6783597.1        ------------------------------------------------------------    0
KAJ6789350.1        ----MPELRPKPNEDGSCATYPVDHNDGCSMIAVARQLTVDDLEEFN-KNTWGWQGCEA-    54
DAA05857.1          SSGS---SKPLPEANGDCFSYTIKAGDDCSSIGMPWNLTPKDIEGFNQKVTWGWRGCPN-    410
KAJ6784990.1        SSGTLPDHRPKPGSDGSCNVHKIEKNDGCWAIADKFGITQDDIEKFN-KKTWGWAGCDH-    386
KAJ6787215.1        SYGKLPDRRPKPNADGTCATYTTQPNDDCSVIAAKRDLVAKDIEEFN-KNTWGWKGCEI-    503
KAJ6784672.1        GEGTLPDYRPQPNSDGTCANYQIHDNDTCYVIADGNYLKSDDIEEMN-KSTWGWSGCEH-    396
KAJ6781350.1        ------------------------------------------------------------    0
KAJ6786554.1        SAGKLPDYKPKPSKDGSCATVKVEKGLDCSSIGASYGLTKKEIEGFNAKKTWGWNGCDGG    394


KAJ6785998.1        ----------------------KSECGKN-----------AKV---PGQKCPLNVCCGKW    162
KAJ6779834.1        ----------------------KSECDAG-----------WGMQWSKAGPCPLNVCCSEY    123
KAJ6783597.1        ------------------------------------------------------------    0
KAJ6789350.1        ---------------PMPNPVENAECGPTVPGTKPPPA---GISLADLNQCPLKVCCNIW    96
DAA05857.1          -LTVGLKICLSKGSPPMPAPVSNAVCGPQKPGTVRPESVKDAFELAKLNPCPLNSCCNVW    469
KAJ6784990.1        -LQPDQLICLSTGRPPFPLPVEGAKCGPMKPGSKPIAG---DDDYSKVSPCPLNACCDVW    442
```

```
KAJ6787215.1          -VFKDFKMCVSKGDPPMPAPVSNAVCGPTVPGTPQPPK---GTELKTLNECPLKACCNVW    559
KAJ6784672.1          -LQVGQIICLSKGDPPMPAQVQGTTCGPQVPGTKRPTD---GTALADLNPCPLNACCDVW    452
KAJ6781350.1          ----------------MPDPIADAVCGPQVAGTKKPID---MSKISELNPCPLNACCDVW    41
KAJ6786554.1          RLWAGTVICVSKGTPPMPSVDPKAVCGPQKSGSKKPKD---MSKISELNPCPLNACCDVW    451


KAJ6785998.1          GFCGMTEDYCDAE--DHGA-------TGGCQSNCDQPGPKNK-ASDQLNRVIGYYEAWRH    212
KAJ6779834.1          GFCGTTQDFCNGK--KVTS-------P----------SCSAS-AGSANKRIIGYYEGWNH    163
KAJ6783597.1          ------------------------------------MDIISSA-PPK-EFISIGYFEGYGL  23
KAJ6789350.1          GHCGFSDDFCTVSKSKSGAPGTAALGINGCVSNCGRYIVGSPSAPE-KPLRIGYFEAWNK    155
DAA05857.1            GQCGIDALFCTKADGPTGNPGTAPAGSNGCISNCGTGIVNNAKPPSGGFRRIGYYEAFNW    529
KAJ6784990.1          GFCGITSDFCTKTPADTGAPGTAKEGTNGCISNCGTDIKKSD-PPS-SLMKVAYYEAWNF    500
KAJ6787215.1          GQCGLTDDFCTYAPSKSGAPGTSA-LQNGCVSSCGRELKKGP-APD-KKMKVAYFEAWNG    616
KAJ6784672.1          GFCGTTDEFCTKTPSDTGAPGTAKPGTNGCISNCGRDLVNNDKAPD-EFRRVGYYEAFGM    511
KAJ6781350.1          GQCGITDEFCTDT--GTGAPGTAAPNTNGCISNCGTKIVKGS--VS-EFRSIAYYEGFSF    96
KAJ6786554.1          GQCGTTDEFCTDT--GNGAPGTAKKDTNGCISNCGTKIITGN--QK-EIRSIAYFEGYAF    506
                                                                   .     :.*:*.:

KAJ6785998.1          DSKCQDMGLDDIPVNSLTHLYFSFAFITPGEYNIVGMNGLPNRLFTDFTSLKKNNPGLKT    272
KAJ6779834.1          QRSCDTMKPSEIPLGYYTHINFAFALIHPTQFTIDAMDDKTGSLYREVTGLKSKDPGLKV    223
KAJ6783597.1          TQPCDRVDVRTIDVKKYTHIQFAFSTVTANTFEVDMGPTL--NQFYYFKQLN----GVKR    77
KAJ6789350.1          NRNCLTMDNH--------------------------------------------------    165
DAA05857.1            ERPCLHMESKLSNTDKYTHMHWAFGDIKSD-FSVYINDTH--HQWDGFMGLK----NVKR    582
KAJ6784990.1          DRSCLHMNVDAIKG-DYTHIHFAFATVDKN-WGVSLDKN-TQKQFNRFKSVK----GPKK    553
KAJ6787215.1          NRPCLHMDVTDIDTSLYTHIHFAFAEVTRD-YNVDISKVQ--KQFDKFKSMT----GIKK    669
KAJ6784672.1          NRPCMNMGVKEIDVNHYSHIHFAFATVTAS-FQVDISDSK--EQFGDFVQMK----NIKK    564
KAJ6781350.1          SRKCLYQDALQIDGSQYTHLHFGFGDISPD-FQISIGDEMATYEFNNFRFIR----GPKK    151
KAJ6786554.1          NRKCQYQDAFQIDPKKYTHLHFGFGTISKD-LKPSIDDYATNYEFNNFRMIS----GPKR    561
                         *

KAJ6785998.1          IIAVGGWTHNDPGPLQKVFSTMVSTKETRAKFIKNLLSFLRLNAFDGVDFDWEYPGADDR    332
KAJ6779834.1          WIAIGGWAFNDPGPTQSTFSLVSKAPAAQELFFNSLITFLINNNFDGVDIDWEYPAADDR    283
KAJ6783597.1          ILSFGGWTFSTDPATYGIFRTG-VTPEHRQTMANNIAKFIIDQGLDGVDIDWEYPAAPDI    136
KAJ6789350.1          -------------------------------------------ELDGIDIDWEYPGSY--    180
DAA05857.1            IVSFGGWGFSTGVASYDVLRKA-MTPENRGHFVSNVVAFAKKMGIDGIDLDWEYPGAPDI    641
KAJ6784990.1          ILSFGGWAFSTEPDTYQRFRDA-TKAENREKFATNVVKFLNDNSLDGLDFDWEYPGAPDI    612
KAJ6787215.1          IISFGGWDFSALPGTFDILREA-VKPANRNVFKQNVVKFVKDNGLDGVDLDWEYPGAPDI    728
KAJ6784672.1          IISFGGWDFSTKPETFQRFRDA-TKPENRDAFIASLVDFASKNAIDGLDFDWEYPGAPDI    623
KAJ6781350.1          IISFGGWEFSNAPSTYDIFRRG-VQDGNREILAKNIAKFVNDNGLDGVDIDWEYPGATDI    210
KAJ6786554.1          ILSFGGWDFSTGPDTYTIFREG-TKKENRETLATNLAKFVEKYQLDGIDIDWEYPSAPDI    620
                                                                  :**:*:*****.:

KAJ6785998.1          GGVP-----EDGVNFTQFLKELDDENNKQPKKYIVSFTAPTSYWYLRHFDLKAIE-HVDY    386
KAJ6779834.1          GGKP-----EDFKNFVSFIAKLRARLNASGKDFGLSLTLPSSYWYMQHFDIIALEPYVDW    338
KAJ6783597.1          PGIPAAD-PAEGANYLKFMIALRGV---LPKDKSLSFAAPASSWYLKGFPIAIITKIVDY    192
KAJ6789350.1          ----------------------------LSASKSVSFAIPASYWYLKAFPIAMMSKRFDY    212
DAA05857.1            PGIPKGL-PSDGPNYLETLRALRKA---LPSKYSLSIAVPASYWYLRPFPIREMSETVDY    697
KAJ6784990.1          PNVPPGS-TDEGSNYLAFLKVLKGK---LDKSKSISIALPSSFWYLQAYPVKELNSVVDY    668
KAJ6787215.1          PDIPSDD-PHNGVNYYMFLKQLKSA---LG-DKSVSFAAPSSYWYMRSYPVDLMARDIDY    783
KAJ6784672.1          PDIPPGD-KGEGDNYLEFLKDLKAK---LPSDKSVSIALPASYWYLKQYPVKKMADIVDY    679
KAJ6781350.1          PGTPAGDPKTEGINYLKFLVVLKEM---LP-NKSVSIAAPASYWYLKNFPIKRISQIVDY    266
KAJ6786554.1          PGVPPGD-KSEGKNYLEFLKLLKSK---LK-GKSVSIAAPASYWYLKGFPIKDIGKVVDY    675
                                                    :*:: *:* **:: : :  :   .*:

KAJ6785998.1          VNVMSYDLHGVWDSSNP-------IGSHIYGHTNLTEMKLAFDLFWRNDVPANKLNMGLG    439
KAJ6779834.1          FNMMTYDIHGTWDGTIPS------LGPRVQAHTNLTEIELAMELLWRNNINPERVTMGLG    392
KAJ6783597.1          VVYMTYDLHGQWDYGNKWSSPGCPGSNCLRSHVNMTETINALSMITKAGAPSNKIAVGLT    252
KAJ6789350.1          IVFMT-----------------------------------DALAMITKAGVPANKIALEIA    238
DAA05857.1            IVYMAYDLHG--------------NGNCLRSHVNQTEVVLALSMITKAGVSASKVVVGES    743
KAJ6784990.1          FIYMTYDLHGQWDYDNKFTTPGCANGNCIRSHVNITETKTSLAMVTKAGLSSNKVVVGVS    728
KAJ6787215.1          IIFMTYDLHGQWDYGNKWTSPGCDTGNCLRSHVNDTETKDSLIMITKAGVPTNKVVVGVA    843
KAJ6784672.1          FIYMTYDLHGQWDVGNKYATPDCATGNCLRSHINKTETMDSLILITKAGVESRKIVVGIS    739
KAJ6781350.1          IIYMTYDLHGQWDAGNPNAQPGCSDGMCLRSQVNLTETMSSLSMITKAGVNSGKVVVGVT    326
KAJ6786554.1          IIFMTYDLHGQWDADNKNSQIGCPGGNCLRSHVNLTETRNSLSMITKAGVDSGKVVVGVT    735
                      .  *:                                   :: :. : .    :: :

KAJ6785998.1          FYGRAFQLADSSCDKPGCLFKGG-----ATKGACSGESGILSYREIREILDKNK---MTP    491
KAJ6779834.1          FYGRSFTLKDPSCTAPGCPFSGP-----GKPGECTGTGGVLSAGEINRAIKKGG---K-M    443
```

```
KAJ6783597.1       SYGRSFKMLTSGCTGPMCTFAGP--QSQAKPGRCTGTAGYISQNEIDYVLRNNP--SAQK    308
KAJ6789350.1       TYGRSFKMAAKGCWGPMCLFTGTNRISNAAEGRCTKTAGYISNAEIQEIIQYGN--VNKQ    296
DAA05857.1         SYGRSFKMAKAGCTGPLCKFTGANGKSEAAAGRCTNARGYLANAEINEIISKSK-GHPKT    802
KAJ6784990.1       SYGRSFRLKLPGCFTPDCLFTGSPTQSDAFQGRCTNTSGYISNAELKEIADKKKYSYVKS    788
KAJ6787215.1       SYGRSFKMEKAGCDGPMCKFTGSARVSNAAHGRCTDTGGYISNAEIAEIIDSGK--VTRQ    901
KAJ6784672.1       SYGRSFRMSDPSCSGPMCTYTGTRDHSEAYQGRCTGTSGYISNAEMQEIIQDHNYGTINN    799
KAJ6781350.1       SYGRSFAMAEAGCHGAQCKYTGSRLQSNAAKGECTDTSGYISNAEIGKILADPS-RVNEH    385
KAJ6786554.1       AYGRSFAMSDGSCHGPDCTFTGTRLHSNANKGECTDTAGYISNAEIKKIAKDKK-RVVKS    794
                    ***:* :   .*     * : *      .  * *:    * ::   *:

KAJ6785998.1       VHDKEAGVKYLTYNN---DQWVSFDDKETFKQKKDLASDLGLGGFLIWAIDQDDAEMSAL    548
KAJ6779834.1       VLDKPSAAQIVTFGG---DQWVSFDDSTSLGLKQKWANEHCIGGLMVWAIDLDDGSLLD-    499
KAJ6783597.1       THDAESNSDILVDDT---TEWVSYMDRDTKEKRKELYQALHFLGTSDWAVSLDGADLVK-    364
KAJ6789350.1       WEA--VGSHVLVYND---TEWVAYMDDDDKKKRADYFSQSNFGGTSEWAIDLRRFSSTG-    350
DAA05857.1         WYDKDTASDYLVYND---VEWVAYMSDKTKQSRREKWKGLNFLGTVDWAVDLQEFNVTD-    858
KAJ6784990.1       WYDKDSDSNIMVYGDGPKHDWVAYMDDDVKSKRLDLYKGYNMAGTTDWAVDLAEFLKDD-    847
KAJ6787215.1       WKA--AGSDILVYSG---TEWVAYMSDDTKAERAKFYDSYNFLGTTDWAVDLQDFDNGD-    955
KAJ6784672.1       YYDSSSDSLILEYGDGNAVDWVAYMDDELKESRASWVKGLNFAGTSDWAVDLNDINGGG-    858
KAJ6781350.1       YIDAGSNSNVLVYDN---TEWVAYMTPELRKTRTSIYKGLQMGGTTNWATDLEDFNDVP-    441
KAJ6786554.1       YMDSTSNSNVLVYDN---NQWVAYMDSSVRSIREKLYKGWGMGGSSNWATDLEDFEDPP-    850
                             :  .     :**::          :  .  .   : *   ** .

KAJ6785998.1       QAVLDPKPLGDFKSIDKGDDNWTGTNEKCYVTAC-GEGCKAGEIKITDEYCAKKKVSTLC    607
KAJ6779834.1       -ALFGNTGHKKRKTRDPKD---NDYNLKCFAGGI-NGG----------------------    532
KAJ6783597.1       ----------------PDPSTIDANGPSLLADGLTDLKLHSSCLPYREIWTA------WD    402
KAJ6789350.1       ----------------TGMDQQLDESFI-------EIRCDGQYTTFDAVE----------    377
DAA05857.1         ----------------HTEPPQGGPGAN-------ETGCINVFDNMIWDWVNPSIEAAIG    895
KAJ6784990.1       ----------------GGSPDDPTNDPS-------KLTCKDAKMSYKKQDI-SPIYFPTY    883
KAJ6787215.1       ----------------DGDDDDDDG-MHGSAD--YFGECTGSYDSLESL-EKAKDSIPDY    995
KAJ6784672.1       ----------------GGDDS-DMPDIDMSDG--DNINCPADMAPGSLEDLSNANNLPMR    899
KAJ6781350.1       ----------------DGVKDWANFRLQIKAG--NN--------PLTHAGDRTGNWTKLT    475
KAJ6786554.1       ----------------KGFPDWGAFTVSLKSR--KD--------PYEDVGKRSGNWTRLD    884

KAJ6785998.1       CPLSGAPDPKDCTWRGGTTRWCNGRCHDDEVMTHMSKYGGGHDCWDGQIAHCCKSALGEY    667
KAJ6779834.1       ------------------------------------------------------------    532
KAJ6783597.1       ----------------------------DAG-----TIAKAPYNWSRFNK-YQKVL----    424
KAJ6789350.1       -------------------RA-------LQA-----M----HDA---LDR-YKKLVDGGY    398
DAA05857.1         CTNILQPSPLSTTVTL-TAYT-------TLT-----LQSGTKLSTSVVSA-PFSISEVSY    941
KAJ6784990.1       GDAL---------------TT-------YVV-----IVNLTPHKFKI-------TSTSHY    909
KAJ6787215.1       CVDQ---------------YA-------TQV-----QQAMLRDAIKRYD----DLVNGDY    1024
KAJ6784672.1       CQAL---------------YA-------LDI-----LYNTLEDSLSLFD-----TNSQDY    927
KAJ6781350.1       CDDK---------------YW-------TEK-----LDYTPAERWAGLD-----AK----    499
KAJ6786554.1       CTAD---------------GA-------TDT-----LHYTSDERWENLD-----AA----    908

KAJ6785998.1       NVC-KWRGVGEKCHAGELPMTFSGTVLNILEDIAKVILRVVGRAYPLTALTGELLLKVLD    726
KAJ6779834.1       ------------------------------------------------------------    532
KAJ6783597.1       -------NN--------YLGKKSGQVPILITD---AIWKNFERHSEIHHGPREGLRIYL-    465
KAJ6789350.1       DDKF---NY--------YSNYIKMLVPMQIDE-----FMASGKADD-----------YF-    430
DAA05857.1         QPFI---ID--------YPDLLSKS-DGQM--------ITYNPTPQIT-PD----PITI-    975
KAJ6784990.1       QYDIDFKDV--------YPGSMMQGA-----------FSRGNGGSPVDDHA----EIYY-    945
KAJ6787215.1       NHYF---DV--------YAGYVKSQVPSQIED-----FMGSKDAQK-----------YF-    1056
KAJ6784672.1       DDKF---GY--------YEQWVKDGIDSRIKD-----FLNFRDDVK-DAPG----IDFF-    965
KAJ6781350.1       -------------------DAW-----TDIIND-----WKQYRKERP----N----SA---    520
KAJ6786554.1       -------------------NAW-----KDMVAD-----WKRYRAKTS----G----VN---    929

KAJ6785998.1       ELHLDTKKLYCCPEEELKKWKNCAWYGKPGNC---------FDGHCPDMKTVQITDSYFG    777
KAJ6779834.1       ------------------------------------------------------------    532
KAJ6783597.1       -NIY------------------CDQY------ALPRKFT-----EKPKEERCNKLNRD--    493
KAJ6789350.1       -TCQGTMYGYHC--------SDCQWG---GCLDSCRRAEDCKSGMYTEKVKCPDTLKY--    476
DAA05857.1         -RIPDG------------------WTVTGGHLASPGSGPGSSSGPSSKPAKATSTTSD--    1014
KAJ6784990.1       -DVEGTDKK------------------------FVIRL-----------------TNND--    962
KAJ6787215.1       -KCTETGKPNCC--------GSCRYFCEEDDVKNCDRTPGCDS------NKSGTRDID--    1099
KAJ6784672.1       -DCKYT---------------------------------WGTK-------------EGT--    977
KAJ6781350.1       ------------------------------------------------------------    520
KAJ6786554.1       ------------------------------------------------------------    929
```

```
KAJ6785998.1        GGETCGGQLSRVRTFCCESDGE-------PLFLPVPLTNLFEHPPDGGVDTDFTLKTDKS    830
KAJ6779834.1        ------------------------------------------------------------    532
KAJ6783597.1        -DVVAA--TYRFPGTWF-------WSEYYILLCPAFLGKI---PKYQ-----FTDLKDL-    534
KAJ6789350.1        ----RE--SGVLPEHYRL------MSADYHL-----------------------------    495
DAA05857.1          ----DG--AGYLLPITFYP------TLSYRI--PSILTPK---PPAP-----T-KLPD--    1049
KAJ6784990.1        ----KG--TGTFERVICDLRGIGIGSRELNV--PGQ---------VP-----L-VITG--    997
KAJ6787215.1        ----CP--TDVVQHEFLDSSRL--HNATFNL-----------------------------    1122
KAJ6784672.1        ----TS--CAGMPHFWDYNDRS--WTVEFIL-----------------------------    1000
KAJ6781350.1        ----KE--TGAFSQFISYLIG---------------------------------------    535
KAJ6786554.1        ----TG--TALFSEFFANNFN---------------------------------------    944

KAJ6785998.1        SDHGDSDPNDAAFQFVVLVSPESLQVSLDKRDGSHWDVFECNDSVSEGEHTVRMVCNDHT    890
KAJ6779834.1        ------------------------------------------------------------    532
KAJ6783597.1        KKEADSYP----LLQTE-INR-W--SGTKRGVTMYYEVA---------------------    565
KAJ6789350.1        ---DDIEG----FW-----KE-W--AKNTASMSHGWPLM---------------------    519
DAA05857.1          ---NDEHP----IN-----TP-S--SSTSSGRPGDGQQS---------------------    1073
KAJ6784990.1        ---SKRAG----FR-----SS----L-LVDGKVAGWMQS---------------------    1019
KAJ6787215.1        ---KDHDG----FF-----KA----IGDKYGIDEEWIQF---------------------    1145
KAJ6784672.1        ---KDEKG----FY-----DA----LAAQTGIDKDWITF---------------------    1023
KAJ6781350.1        ---------------------------NPPDPHCEDILT---------------------    547
KAJ6786554.1        ---------------------------HTQPIDCGDITV---------------------    956

KAJ6785998.1        ADSNCHKIGLGHGVPGTILQMPKGCGPGKYAVAKS-----MAPAPGK-DHVKLL-PRHLS    943
KAJ6779834.1        ------------------------------------------------------------    532
KAJ6783597.1        ---------HLQDVS-----------NNPTCDNNE-----------------LYDPRRI-    587
KAJ6789350.1        ----------------------------IDCARKSDFYFHHYP--KVKDDMKVPNPKDV-    548
DAA05857.1          -SGTPRPPSNGQNPSST-P-KPPGDGPDPINTQK---PPGDGPDPINT----QKPPGDG-    1122
KAJ6784990.1        -IKDTIKD----------------------------RRIKDVVLPGTHDSAMSKISHKL-    1049
KAJ6787215.1        -SRKTMHVSNGCQYAGT---------HVKECQDKQDSHYYNFPL-ANNDKIHVFNPKDM-    1193
KAJ6784672.1        --GTKEEPYVCADPSGDAP-LRPGGGAATPCHRLT-HTLKNYPTKIDDKDIHVGNPKKF-    1078
KAJ6781350.1        -GNNCRNTFDCKSFKGDK----------------------------------SGPAAAL-    571
KAJ6786554.1        -SSNCRSAVTCDDFTGER----------------------------------TGPAAAL-    980

KAJ6785998.1        HLAGLEPVVYELTFDYDFHRVPRDL----GDTQMRIDFSNQEDYWKNIVAGSV---NRKR    996
KAJ6779834.1        ------------------------------------------------------------    532
KAJ6783597.1        ----VLKAQGGYDRDYEFHLANAHSWTLSALGMWM-------MKTFNLSDPPMPRGTQIQ    636
KAJ6789350.1        ----IGKSYNEALDLLKYFKI--------ARSAII-------YDSLAAEDLLDAISVSAF    589
DAA05857.1          ----PDPSNAHNT----------------PASTKRPG-----DNQH-----PTPTPAPIR    1152
KAJ6784990.1        ----ITAADSPNTQTQ--GMS---IYN-----QLRLG-----ARWF---------DLRVI    1081
KAJ6787215.1        ----IDKGYKNMTTTRDDLDL---LLAA---SQYQIG-----MAW---DDAADGTDIPAM    1235
KAJ6784672.1        ----IEASMTNITALQDSLLS---SYAAVGLGVYSEG-----KTDVSEIDPVTAYAMPVL    1126
KAJ6781350.1        ----IWNSLVKVSSTY----A---NHLL-PLL----------PLL-----PLLP------    598
KAJ6786554.1        ----IWNSFVQINEMY----A---SYAD-ALG----------NAAAEKIDPSLD------    1012

KAJ6785998.1        DVNGRKRLAKRTLEDVGGNPVRWLEEEFRDDFHFDKIASRELQERWFGK------SILEW    1050
KAJ6779834.1        ------------------------------------------------------------    532
KAJ6783597.1        QRDGDDGSGDETGNDIV--------DQN-DDFDPSQID--PTEFFWMSRLGYVPNRLHLY    685
KAJ6789350.1        TYQSAVEK----MDAIV--------KEV-KTIKKN--D--LKE------------FILDF    620
DAA05857.1          D----IDC----KDDSC--------TRG-RDCESD-------D------------CL---    1173
KAJ6784990.1        SVHGTAGM----EGDYE-------------------------------------------    1094
KAJ6787215.1        SVVSAVDN----MKEIR--------DKG-KEIEKK--E--REQ------------FILNM    1266
KAJ6784672.1        QLQQSIDS----MRDIK--------DIG-EKAKEE--K--KKQ------------LIMTI    1157
KAJ6781350.1        ---------------------------L-LPLLPL--L--PLH------------LLTL-    613
KAJ6786554.1        --------------DFQ--------NTF-APTPPE--K--DNE------------WLTIL    1033

KAJ6785998.1        --LAALVKPEIRREFT----YKYDDSVTAKLFDESWDCPGSVE---GVNYDGHLLAQAVL    1101
KAJ6779834.1        ------------------------------------------------------------    532
KAJ6783597.1        VTLVVLVIPPFPVKYICAHDYGVNTAVVIAK----------RA---NMNIKANAKHIVVW    732
KAJ6789350.1        ITGVIMLVP-GIGEAL-GPEMA--ATRS------FLRLLGDVGDA-GTLIYGVVED----    665
DAA05857.1          --------------RG-GDCEG--ENCVEGGKCRGKRCISGGNCK-G-------------    1202
KAJ6784990.1        --------------------FYG--AHVNDEK---MSACIGNTG----QSLDDIISDINQF    1126
KAJ6787215.1        VLGILFFIP-FVGEAA-GAAGL--TAV--RT---LCRLIGDLGDI-GMTIYGIVEN----    1312
KAJ6784672.1        LTVVFMVLP-FVGEAL-GPIVG--AASSIAR---IALIISEAGNA-AISIKDIVDD----    1205
```

```
KAJ6781350.1          -HLLTLH--------L-LPLLS--ASCLALL---LRFFVHSSLTEPSS-------H----    647
KAJ6786554.1          LDLVSMG----TSMVA-GPFVG---KVI-GK---LPYFVSKIEDAGANALKDKIAS----    1077

KAJ6785998.1          DI-----------------EVETSFGFTLIVESLTLPLNLDKSYLTF-----------YN    1133
KAJ6779834.1          ------------------------------------------------------------    532
KAJ6783597.1          VL-VPQQVVLF-------------------------------------------------    742
KAJ6789350.1          ----PSSGLMRG------------FGAFAAVGLSRSGI-LK-----A-----SESRRGMS    698
DAA05857.1            ----PK---------------CKTGGPCEGENCEKGGG-CAKTLFGDCGSGGCQGARCFS    1242
KAJ6784990.1          TSENPGEVIFFRLRYMVGIRKVPSLGPIHWGGDDWSNL--YDD---FLGRMQKVHNRCLN    1181
KAJ6787215.1          ----PSDAF------------GAIFGALLGRALD------------HV---------QLG    1335
KAJ6784672.1          ----PTSA------------PFAMLGFIVGAAGAGAGG--S----------------RLT    1231
KAJ6781350.1          ----RPPLI------------------VLNCADDYATA--EDG---KVLYT---------    671
KAJ6786554.1          ----TSSTILTT-------AITIGSGFIDGSTDDWDPE--AED---HFKNTMSLAMTGWN    1121

KAJ6785998.1          KGRVTGVVTLEALARVT-YEKKKVILNLP-----FPGASFKIPGIATIGPQLTVEGSIDA    1187
KAJ6779834.1          ------------------------------------------------------------    532
KAJ6783597.1          ------------------------------------------------------------    742
KAJ6789350.1          DTEIESGE--------VSGDGSRAVLIDPTSCIPASGI----------------------    728
DAA05857.1            KRDCFGAQ-------------CERLTIKPLPKPDPKSTPATPPKPT--------------    1275
KAJ6784990.1          IDGDFGAKKMGTFMDMNEGKGCVIFLLSPTD---FSGNPYR--PQY--------------    1222
KAJ6787215.1          KAAKSGRE--------LKRKGID-------------D----------------------    1351
KAJ6784672.1          KVETLGDA--------AAVRGA--------------------------------------    1245
KAJ6781350.1          ------------------------------------------------------------    671
KAJ6786554.1          KIATFGLR--------DLFNGSD--------------SSLN--TLS--------------    1143

KAJ6785998.1          SLGMAGLVETKLEIAKWEVRQVMPSTDDYKPELIDNSSPS--LDRT-----GDFSGIQKP    1240
KAJ6779834.1          ------------------------------------------------------------    532
KAJ6783597.1          ------------------------------------------------------------    742
KAJ6789350.1          -------S-----------DPRIAIHI---------NKYIRILSITPDNIF--------P    753
DAA05857.1            -----CLVD----------CPKLPECPDWDPLCHDPCPFSAC-----------------P    1303
KAJ6784990.1          -----GLYD----------AGKLVITDDWADTES-----------TKDMVNRETKDWQDP    1256
KAJ6787215.1          ------------------------------------------LKGIKENVMKLNN-----    1364
KAJ6784672.1          ----------------------LK-AEDLA-------KFPARFRELDSQVQKIVKKF---    1272
KAJ6781350.1          ------------------------------------------------------------    671
KAJ6786554.1          -----DLLS----------GGKFAAGKDYGKGSYDQASSKEANKTLEDNLSKTFYAYAIP    1188

KAJ6785998.1          EFYAGVTASGDVTLRLGAAAEF-----------GVRFDDKWKVDPAAAAVVGEVSLTTKF    1289
KAJ6779834.1          ------------------------------------------------------------    532
KAJ6783597.1          ------------------------------------------------------------    742
KAJ6789350.1          V-----------------------------------------------------------    754
DAA05857.1            VYRRPTG-KACTTLQTARDCTEFVSSTRVKTKPTTSWSTTTRTLCET-------------    1349
KAJ6784990.1          RKFINIH-IAQWLL------------TS-QGIDAVSWDNYYEAMQVN-------------    1289
KAJ6787215.1          -------------IRGARMCKL--------------------------------------    1373
KAJ6784672.1          ---------------EGKACHL--------------------------------------    1279
KAJ6781350.1          ------------------------------------------------------------    671
KAJ6786554.1          AIWHASG-HRPFIIDTGLSCDKGKA-ED-VKLKGACWKDTWYRLASP-------------    1232

KAJ6785998.1          AAGSSTTGTCPFTYGLDVGARLFARAIAPQVFGWGGGEVDLTAKWEKSIIEGGTCPDLGP    1349
KAJ6779834.1          ------------------------------------------------------------    532
KAJ6783597.1          ------------------------------------------------------------    742
KAJ6789350.1          ------------------------------------------------------------    754
DAA05857.1            ----------------------MVDCEA----------ADITAT-----TTITTTHTPDP    1372
KAJ6784990.1          -------------------PLIYS---------------------------HGVN-----    1298
KAJ6787215.1          ------------------------------------------------------------    1373
KAJ6784672.1          ------------------------------------------------------------    1279
KAJ6781350.1          ------------------------------------------------------------    671
KAJ6786554.1          -------------------DGGYQECAK-----------------------NGCDHVPNP    1250

KAJ6785998.1          IPSKRSLQSLDPPSIDGLSEDRHVTRNKSRLGGADLG-------SYSASDVQSMHS----    1398
KAJ6779834.1          ------------------------------------------------------------    532
KAJ6783597.1          ------------------------------------------------------------    742
KAJ6789350.1          ------------------------------------------------------------    754
DAA05857.1            IESV-------GPAVPYYGVSWSFGEKEKS-------SMLADEEAYFSALETPMTTTTTT    1418
```

```
KAJ6784990.1        --EMSPTAWPGVLMVDYLGETWYAGHDFAD-TGAELATLAVGLNQY-------MLSENCD    1348
KAJ6787215.1        ------------------------------------------------------------    1373
KAJ6784672.1        ------------------------------------------------------------    1279
KAJ6781350.1        ------------------------------------------------------------    671
KAJ6786554.1        CVECDDDQFKTPSGVDKLGSDYFSQVSVHD-LIV--GYVRTGTPNFFSTTTSRKLTAKCR    1307

KAJ6785998.1        --------------SEEHTSSPAKRGSLVKRGGVYGPAFSLPVGEFFCPSK-DGEKGMTC    1443
KAJ6779834.1        ------------------------------------------------------------    532
KAJ6783597.1        ------------------------------------------------------------    742
KAJ6789350.1        ------------------------------------------------------------    754
DAA05857.1          SAEPSTTDDTPTETTADGPTSTVGPNDLVCGFALYAAFYRFDIVKM-VGDWVWDDEGHK-    1476
KAJ6784990.1        LSSI------------------------------YPPLLDYPEGYKDKSANKAN---NK-    1374
KAJ6787215.1        ------------------------------------------------------------    1373
KAJ6784672.1        ------------------------------------------------------------    1279
KAJ6781350.1        ------------------------------------------------------------    671
KAJ6786554.1        SVRTWLDNDKENGGGSANPNSPDTLKSLLQDDVTTPGFIRMPVCTADRARIAWGASSDS-    1366

KAJ6785998.1        VQAYDALAESNEGGVWQNMRKRDTLVPTSTIDEN---AVAAHFHDH--------------    1486
KAJ6779834.1        ------------------------------------------------------------    532
KAJ6783597.1        ------------------------------------------------------------    742
KAJ6789350.1        ------------------------------------------------------------    754
DAA05857.1          LKKELKGCGALTGWKWYRR------------------------DDG----------SREA    1502
KAJ6784990.1        IA--GADEEQWNGFIFANGTVIDHP----SPDV------------------GVYNGPHKD    1410
KAJ6787215.1        ------------------------------------------------------------    1373
KAJ6784672.1        ------------------------------------------------------------    1279
KAJ6781350.1        ------------------------------------------------------------    671
KAJ6786554.1        AK--NYPCNANAGYDYCGDSTIENQTSDASPDVEDCKEMIRRLEDGRLPKRWPHVGPLEK    1424

KAJ6785998.1        -QRSHGYR---RHVRGEELHELE-KRAKEKLIKACERTCDIS----------ADFPPGGQ    1531
KAJ6779834.1        ------------------------------------------------------------    532
KAJ6783597.1        ------------------------------------------------------------    742
KAJ6789350.1        ------------------------------------------------------------    754
DAA05857.1          -----------------------RFNLPVFLTAGCVESAIKSAGGPGLS-------CIFA    1532
KAJ6784990.1        -DAIITYYS---------------------------------------------------    1418
KAJ6787215.1        ------------------------------------------------------------    1373
KAJ6784672.1        ------------------------------------------------------------    1279
KAJ6781350.1        ------------------------------------------------------------    671
KAJ6786554.1        QQAIATYESCKFGVTGKGIHGNVDFKVGAQDVIDLVRDSIDKFGGGGKVGSKGTMSCKGN    1484

KAJ6785998.1        LKGKEWGWATKDCGDFNFGSPLLAREPNTDYHTEHVLEAQMIDLFFQYLNKKKKPQPDPK    1591
KAJ6779834.1        ------------------------------------------------------------    532
KAJ6783597.1        ------------------------------------------------------------    742
KAJ6789350.1        ------------------------------------------------------------    754
DAA05857.1          T-----------------------------------------------------------    1533
KAJ6784990.1        ------------------------------------------------------------    1418
KAJ6787215.1        ------------------------------------------------------------    1373
KAJ6784672.1        ------------------------------------------------------------    1279
KAJ6781350.1        ------------------------------------------------------------    671
KAJ6786554.1        IKGQKVNWGLY-------------------------------------------------    1495

KAJ6785998.1        PGAAKGDTVSFCDYVNVLWDVPAFVWPGQDMDGGIGKKWDPIMHIAAQFPTKTFNKQEFV    1651
KAJ6779834.1        ------------------------------------------------------------    532
KAJ6783597.1        ------------------------------------------------------------    742
KAJ6789350.1        ------------------------------------------------------------    754
DAA05857.1          ------------------------------------------------------------    1533
KAJ6784990.1        ------------------------------------------------------------    1418
KAJ6787215.1        ------------------------------------------------------------    1373
KAJ6784672.1        ------------------------------------------------------------    1279
KAJ6781350.1        ------------------------------------------------------------    671
KAJ6786554.1        ------------------------------------------------------------    1495

KAJ6785998.1        ALESPINTPSKTRAWASGNPWDSKGWTNDIADYAKSRIILQKLRSTMGSRIYQSDSTISQ    1711
KAJ6779834.1        ------------------------------------------------------------    532
```

```
KAJ6783597.1          ------------------------------------------------------------    742
KAJ6789350.1          ------------------------------------------------------------    754
DAA05857.1            ------------------------------------------------------------    1533
KAJ6784990.1          ------------------------------------------------------------    1418
KAJ6787215.1          ------------------------------------------------------------    1373
KAJ6784672.1          ------------------------------------------------------------    1279
KAJ6781350.1          ------------------------------------------------------------    671
KAJ6786554.1          ------------------------------------------------------------    1495

KAJ6785998.1          TMKTQTDRIGVVLDALDTTLLKANTPAGYTAWEKQDLKDEWHKYMKGQYTTMQSKTRGLV    1771
KAJ6779834.1          ------------------------------------------------------------    532
KAJ6783597.1          ------------------------------------------------------------    742
KAJ6789350.1          ------------------------------------------------------------    754
DAA05857.1            ------------------------------------------------------------    1533
KAJ6784990.1          ------------------------------------------------------------    1418
KAJ6787215.1          ------------------------------------------------------------    1373
KAJ6784672.1          ------------------------------------------------------------    1279
KAJ6781350.1          ------------------------------------------------------------    671
KAJ6786554.1          ------------------------------------------------------------    1495

KAJ6785998.1          NDWLPKLKKAWCSQAEKDKWEAKPTDSGTLKTEKEEHRAFIKAIEDFDTKWNGLAVWNNP    1831
KAJ6779834.1          ------------------------------------------------------------    532
KAJ6783597.1          ------------------------------------------------------------    742
KAJ6789350.1          ------------------------------------------------------------    754
DAA05857.1            ------------------------------------------------------------    1533
KAJ6784990.1          ------------------------------------------------------------    1418
KAJ6787215.1          ------------------------------------------------------------    1373
KAJ6784672.1          ------------------------------------------------------------    1279
KAJ6781350.1          ------------------------------------------------------------    671
KAJ6786554.1          ------------------------------------------------------------    1495

KAJ6785998.1          L    1832
KAJ6779834.1          -    532
KAJ6783597.1          -    742
KAJ6789350.1          -    754
DAA05857.1            -    1533
KAJ6784990.1          -    1418
KAJ6787215.1          -    1373
KAJ6784672.1          -    1279
KAJ6781350.1          -    671
KAJ6786554.1          -    1495
```

Fig. S1. Clustal alignment of *A. album* GH18 chitinases. The alignment of the 26 *A. album* sequences and the three *H. jecorina* sequences (DAA05865, DAA05854, DAA05857) was subdivided for the subgroups A, B and C (see Figure 1) in order to better indicate the specificities of the three subgroups. Boxed sequences correspond to the conserved motif DXXDXDXE, typical of GH18 sequences. Yellow highlighted C's (in the B sequences) correspond to C residues 46, 93, 185 and 214 in hevamine (P23472), a lysozyme/chitinase identified in rubber tree (*Hevea brasiliensis*). Green highlighted sequences in A and C groups indicate the typical extra α/β region of about 70 amino acids described in plant and fungal Class V chitinases (Taira, 2009, Kamerewerd 2011).

Figure S2

```
KAJ6787877.1      ------------------------------------------------------------ 0
KAJ6782998.1      ------------------------------------------------------------ 0
KFG78085.1        ------------------------------------------------------------ 0
KAJ6788372.1      ------------------------------------------------------------ 0
AGC24356.1        ------------------------------------------------------------ 0
KAJ6789110.1      ------------------------------------------------------------ 0
KAJ6785893.1      -----------MMPSKYNALAVLLASSAYARTCLPAYEAQTSYVG------CYHDPNSPR 43
KAJ6786934.1      ------------------------------------------------------------ 0
KAJ6780833.1      ------------------------------------------------------------ 0
KAJ6787866.1      ------------------------------------------------------------ 0
KAJ6786079.1      ------------------------------------------------------------ 0
KAJ6785739.1      ------------------------------------------------------------ 0
KAJ6782204.1      MTSVAKPGDDGLLPSK------------------PKYGSEEDSGDSDIELEELRDEVDAD 42
KFG84234.1        ------------------------------------------------------------ 0
KAJ6788989.1      ------------------------------------------------------------ 0


KAJ6787877.1      -----------------------------------MRI-VEIASSLGLLLA--------- 15
KAJ6782998.1      -----------------------------------MHL-PLTHSLLALTLV--------- 15
KFG78085.1        ----------------------------------------MAAANLNPKKPNSS------ 14
KAJ6788372.1      -----------------------------------------------MTSNNAN------ 7
AGC24356.1        ------------------------------------------------------------ 0
KAJ6789110.1      ------------------------------------------------------------ 0
KAJ6785893.1      DLSGPLLTVGKL------NSPQYCANICGAAGYSYSGVEYTVQCFCGYKIESTSIKADES 97
KAJ6786934.1      ------------------------------------------------------------ 0
KAJ6780833.1      -----------------------------------MKSPLNSYCLAGL------------ 13
KAJ6787866.1      ---------------------------------MVAATKLLSVATAGWAVASAAAA---- 23
KAJ6786079.1      ---------------------------------------MLLPLLAGLALVHPA-V---- 16
KAJ6785739.1      ---------------------------------MVQKTT-----ITSVAG-ALALFS--- 18
KAJ6782204.1      LLPGTRPGVSKKRHRRTEASDHFLARIL---NWRLRKRTVFIVSIVGVLVVLGGLFG--- 96
KFG84234.1        ----------------------------------MKSLSIFAAALLT-AVVDAGLTS--- 22
KAJ6788989.1      ----------------------------------MKTASFVAAALLA-VGTDAAHFG--- 22


KAJ6787877.1      -----------------------------THAKPCAGA--ASDLDILAGQHVIYSFPTS- 43
KAJ6782998.1      -----------------------------SAASGSVKP--ACDLDILAGQHVIYSWDYTL 44
KFG78085.1        ---------------------------SSSDGSDLDPL--WQNLDWAVGQMLIMGWDGTE 45
KAJ6788372.1      ---------------------------NGAESADIDPI--YQNLDWAIGQTLIMGWDGTE 38
AGC24356.1        ---------------------------------MTVGN--DDNLDKEIGQLLMCGFDGLE 25
KAJ6789110.1      ------------------------------------MV--TPEQRKKVGQLFAVGFHDTT 22
KAJ6785893.1      QCNADCPADPSKKCGGGNMINIYKINNP-SANPPLSPSFPDCTRDPLCSNDVCDTSKSIA 156
KAJ6786934.1      ---------------------------------------------------------MAD 3
KAJ6780833.1      --------------------LLPAAVAGRATSPDVYPT---PQGK-GE-----GPWAAAY 44
KAJ6787866.1      ----------------ASDGVITNDTYFYGQSPPVYPS---PEMS-SS-----GAWAEAQ 58
KAJ6786079.1      ----------------SNQIYFRAEPDAYQNSPEVLPS---PQIS-GQ-----NEWADAF 51
KAJ6785739.1      -----------------AQGHASAATASGYDSPAYYPA---PYGG-WV-----SEWRDSY 52
KAJ6782204.1      -------------------WGRYTQTPPDGHSPPWYPC---PRGG-TV-----SQWAESY 128
KFG84234.1        ----------------PGHFHKLDARAELATSPPHYPS---PWMNPQA-----PGWEDAY 58
KAJ6788989.1      -----------NN---TNSNSTTEQTGPLAHSPPFYPS---PWMDPKA-----PGWEDAY 60


KAJ6787877.1      STPPAQLVQLTKAGLVGGVILFGENVDAKTAAAMD--TLRQAYAASPAPA-LLKKT-TGV 99
KAJ6782998.1      PGPPDELYDLTRAGLVGGVLLYDVHVNKDTPAALA--KLQAAYKASPAHK-LLRKQ-SGR 100
KFG78085.1        VTP--QIKSLIEDHHLGSIILTAKNLKS--AQETA--KLVQEL------Q-TIAKN-AGH 91
KAJ6788372.1      VTP--QIRTLIEDHHLGSIILTAKNLKS--AQQTA--RLVQEL------Q-TIAQN-AGH 84
AGC24356.1        PTP--GIIDLIENHNLGSIILFSRNIAT--PKQVQ--KLTHSL------Q-QIARN-AGH 71
KAJ6789110.1      VNN--DIKSLIQDYGVGAIVLFKRNVGS--AAELR--TLCRDL------Q-QLAKD-AGH 68
KAJ6785893.1      DRAAAIVKVLTLDE--------KANNLASSAS--------------------------GS 182
KAJ6786934.1      LNVEEVLKKLTITE--------KVELLSGMSIA------------------------PAM 31
KAJ6780833.1      QSARALVGQMTLDE--------KVNITRGFK-ADN----------------ICAGTTGSV 79
KAJ6787866.1      AKAQKLVSQMTLEE--------KVSLTGGADSIDN----------------GCSGNVANI 94
KAJ6786079.1      AKAKAMVDQMSLEE--------KVSITGGVD-LKS----------------GCSGTIAGI 86
KAJ6785739.1      SKAKKLVDSMTLAE--------KTNITAGTGIFMGKSRLSNQAKASDAAILRCNGNTGSA 104
KAJ6782204.1      EKAARMVRNMTLAE--------KVNITTGTGWAMG----------------LAVGNTGPA 164
KFG84234.1        KQARDFVSRMTLAE--------KVNLTTGTGWMSD----------------NCVGNSGAV 94
KAJ6788989.1      AKAKAFVSQLTLAE--------KVNITTGVGWMGE----------------KCVGNVGSV 96
                        :  :

KAJ6787877.1      ADA--KFFISTDQ---EGGQVRRMKGEE--------PKL--SPKQMGASADPAAAGKAGG 144
KAJ6782998.1      DTA--LLIMTNEE---GGTVFNGVHDGG--------PAL--SAKQVGASPDPAAAGWQAG 145
KFG78085.1        PQ---PLLIAVDQ---ENGGVNSLFDED-------YVCQFPSAMGIAATGRVELAYEI-Y 137
KAJ6788372.1      PQ---PLLIAVDQ---ENGGVNSLFDED-------YVCQFPSAMGIAAAGSPELAYEV-T 130
AGC24356.1        KR---PLFIAVDQ---ENGVVRRLGD-S--------GTYLPGNMALGALGSSTAARNV-A 115
KAJ6789110.1      EQ---PLFIGIDQ---ENGLVTRISP-P-------VASQQPGPMALGAAGSLDIASQV-A 113
KAJ6785893.1      SKIGLPAYQWQNEALHGVAGSTGVQFQSPLGSNFSAATSFPMPILLSAAFDDKMVKDV-A 241
```

```
KAJ6786934.1      PKHGIPSIRMSD-------GPNGVRGTKFF--NGVPAACFPCGTALGSTFNQELLEKA-G 81
KAJ6780833.1      PRLNWPGMCLHD-------AGNGVRAAELV--N-----AWPSALHSGASWDRNLTHQR-G 124
KAJ6787866.1      SRLGFPGMCLSD-------AGNGLRATDFV--S-----SFPSGIHVGASWNKDLAARR-G 139
KAJ6786079.1      PRLKFPGMCLND-------AGNGVRATDYV--S-----GFASGIGVGASWNKVLAKQR-G 131
KAJ6785739.1      PRVGFPMLCLED-------GASGIRQADHV--T-----AFPDGITAGATFDKELMYQR-G 149
KAJ6782204.1      IYAGFPSLALQD-------GPLGIRFADNA--T-----AFPAGITVGASWNKELMFQR-G 209
KFG84234.1        PRLGLRALCLQD-------GPLGIRLSDYN--S-----AFPAGITAGATWSEHLWRDR-G 139
KAJ6788989.1      PRMGLKSLCMQD-------GPLGLRFSDYN--S-----AFPVGVTAGASWARHLWRDR-G 141
                             :           :                       .:

KAJ6787877.1      AGAAATLKKFNNNAN-----LAPVLGV-YREAGDFLDYYGRSFGNSSAAVAAAAVPFIKA 198
KAJ6782998.1      VQAAAALKRYNFNVD-----LAPVLGV-YRDEGNFLDTGNRSYGKTADKVIAATLPFIRA 199
KFG78085.1        KATASEISACGVNLM-----LGPVLDV-LNNAR-YQPLGVRSTGDDPQEASQYGLAALNG 190
KAJ6788372.1      KATATEISACGVNLM-----LGPVLDV-LNNAR-YQPLGVRATSDDPQEVSQYGLAAMAG 183
AGC24356.1        MAISKELLTLGMNWN-----LAPVLDV-NNNPL-NPVIGVRSYGQDPELVARMGLAQVEG 168
KAJ6789110.1      GATAEMLGHFGINMN-----YAPVGDI-NSEPL-NPVIGVRSPGDDPEKVAQFASACAKG 166
KAJ6785893.1      TIISTEARAFANHGFAGLDFWTPNINP-FRDPR--WGRGMETPGEDSFRISNYVVSLVDG 298
KAJ6786934.1      KTMGDEAIAKSAHLI-----LGPTIN-MQRSPL--GGRGFESIGEDPFLSGLGAAALVRG 133
KAJ6780833.1      LYMGGEFKGKGINIA-----LGPNAGPLGRTPL--GGRNWEGFSVDPYLSGQLNAETITG 177
KAJ6787866.1      AAMAGEFKKKGVNVL-----LGPVVGPAWRVTL--SGRNWEGFSADPYLSGSLAAQSVAG 192
KAJ6786079.1      VALGGENRRKGVNIM-----LGPVVGPAWTVVK--GGRNWEGSSADPYLSGALAAETVVG 184
KAJ6785739.1      VALGKEARGKGVNIL-----LGPTVGPIGRKPK--GGRNWEGFGVDPTLQATGARQTIKG 202
KAJ6782204.1      QAHGQEAREKGINVL-----LGPSVGPLGRMPA--GGRNWEGYGPDPYLQGIAAAETIRG 262
KFG84234.1        KALGAESRDKGIDIA-----LGPASGPLGRAPT--GGRNAEGFGSDPYLQGKGLANTVIG 192
KAJ6788989.1      NAMGSEARDKGVDVI-----LGPASGPLGRLSK--GGRIGEAFGADPYLQGQALANTVTG 194
                     .        .         *  .              .  .               .

KAJ6787877.1      QQAAG------VAATA--KHFPGLGAASHDANT--------DTGPVTLEQSLDEIRSIDE 242
KAJ6782998.1      QRERG------ILVSI--KHYPGLGAARKDQNT--------DAAPVTLDVSRADLESIDL 243
KFG78085.1        IRDAG------IAAAG--KHFPSYGNLDFQGSN--------S-AVPVITQTLEELSLSAL 233
KAJ6788372.1      IRDAG------IATCG--KHFPSYGNLDFLGSS--------L-DVPIITQTLEELSLSAL 226
AGC24356.1        YQRGK------VATSI--KHFPGHGDTA-TDSH--------L-DVPVINKTLEELDKTEL 210
KAJ6789110.1      MRENK------VVPCI--KHFPGHGDTA-VDSH--------Y-GLPSIDKSRAQLDAVEL 208
KAJ6785893.1      LQGGINPEFHRTISTC--KHFAAYDVESGRTQN-------------DIHPTRQDMADYYL 343
KAJ6786934.1      IQSTG------VVATI--KHFLCNDQEDKRMGV-------------QSIVTERALREIYA 172
KAJ6780833.1      IQKAG------VIANI--KHFIANEQETYRRPY-------FGVEAASSNVDDKTLHEYYL 222
KAJ6787866.1      IQGQG------VITST--KHFIANEQETNRNPD-------QDVQSLSSNIDDKTMHELYL 237
KAJ6786079.1      VQSQN------VMTSVKKQHYIGNEQELYRSLE-------GDIQAISSNIDDKTMHELYL 231
KAJ6785739.1      IQEQG------VIATI--KHLVGNEQEMYRMYN-------PFQQAYSSNIDDRTLHEVYL 247
KAJ6782204.1      IQGEG------VMATI--KHFVANEQEHFRQP-----WAWGLPHAVSSNVDDRTMHELYA 309
KFG84234.1        IQESG------VIACA--KHFIANEQEHFRQAGESVPRGFNISESLSSNVDDKTLHEDYA 244
KAJ6788989.1      IQGAG------VVACA--KHYIANEQEHFRQVGESVDRGYDISETLSSNVNDKLMHEYYS 246
                   :                :*                                   :

KAJ6787877.1      APYVGAIA-AGVELVM----MSWA--VYPALDG-RPAGLSNKWVQDELRGRLGFTG---V 291
KAJ6782998.1      RPFQAAIR-AGVDMVM----ASWA--VYPAVDGAWPAGLSSRWMKDILRGRLGFRG---V 293
KFG78085.1        VPFRNAIA-TGKLDAM---FVGGCGISNPSMNVG-HACLSDQVVDDLLRNELGFKG---V 285
KAJ6788372.1      VPFRNAVA-SGKLDAM---FVGGCGLSNPSMNVS-HACLSDQVVDDMLRNEMGFRG---V 278
AGC24356.1        VPFKKALE-AGGIACPTSVMVGHM--LLPHFNKDVVSSIAPEIVRDLLRRRFGYKG---V 264
KAJ6789110.1      IPFKRAAQ-EGIE----MIMTAHI--AMPQLTGSDMPSTLSPQVLSILRDEFKFDG---V 258
KAJ6785893.1      PMFERCVRDAKVGSVM-------C--AYNSVDGI-PACASSYLLQTVLRDGWNFKEPYNY 393
KAJ6786934.1      MPFQLAVRDANPGAFM-------T--AYNGINGT-FCSENPKYLDGMLRKEWGWDG---L 219
KAJ6780833.1      WPFMDGVH-AGVGSVM-------C--SYNRINNT-YGCENSKLMNGLLKGELAFDG---F 268
KAJ6787866.1      WPFQDAVH-AGSGNIM-------C--SYQRINNS-YGCANSKTLNGLLKSELGFQG---F 283
KAJ6786079.1      WPFVDLVK-AGTANVM-------C--SYNRLNQT-YACGNSKTLNGLLKTELGFQG---F 277
KAJ6785739.1      WPFAEGIH-AGVGAVM-------T--AYNAVNGS-ACSQNPYLINGILKDELGFQG---F 293
KAJ6782204.1      WPFANAVK-AGVASVM-------C--SYNMVNNS-YACGNSKLLNGILKDELGFQG---F 355
KFG84234.1        WPFADAIR-AGVGSIM-------C--SYNQINNS-YACQNSKLLNGILKDEMGFQG---F 290
KAJ6788989.1      WPFQDAVK-AGVGSIM-------C--SYNQVNNS-YACQNSKLLNGILKHEYGFQG---F 292
                    :                           .            :   *:    :

KAJ6787877.1      TITDAMEAGSLKG----FGGAEETAKLAAAAGMDLLLASGRNVTQG--DTIRKAVVSGIQ 345
KAJ6782998.1      TITEAMEAGSILP----FGDVEERTKIAVRAGNDLILASGLNVTEG--EVIRKALVEGLK 347
KFG78085.1        AISECLEMEALSH----DLGVQNGVVMAVEAGCDLVLLCRAYDVQL--EAI-KGLKLGYE 338
KAJ6788372.1      AISECLEMEALSH----DLGVQNGVVMAVEAGCDLVLLCRAYDIQL--EAI-KGLQLGLE 331
AGC24356.1        IITDCLEMDAVKE----TVGTPKGALMALQAGNDMAMISHTLAFQK--DAF-KVLYSALQ 317
KAJ6789110.1      IMTDCLEMDGIRA----TYGTVEGALLAFQAGVDNVMICHTYDVQT--AAV-DRICAALE 311
KAJ6785893.1      VVSDCDAVDNIFDPHHYAKDRVEASALGINAGTDLDCGD---------TY--TNLNQGVR 442
KAJ6786934.1      IMSDWYGTYSTND--------------AVKAGLDLEMPGPS-------RFRGEALQFNAS 258
KAJ6780833.1      VMLDWNAQHNL-N--------------SANAGLDMLMPMG--------GNWGQNLTNAVR 305
KAJ6787866.1      VVSDWGAQHTGHA--------------GALAGMDMAMPNP-------GSFWGDELVKSVK 322
KAJ6786079.1      VVSDWGAQHAL-T--------------DAPNGLDMGMPNS-------DPYWGAALVNAVK 315
KAJ6785739.1      VMSDWLGHMSGVE--------------SALAGLDMDMPGDTQVPLLGYSYWMFELTRSAL 339
KAJ6782204.1      VMSDWLAQQSGVA--------------SALAGLDMSMPGDGLGWTDGESLWGPQLSRAVL 401
KFG84234.1        VMSDWQAQHGGAA--------------TAVAGLDMSMPGDTE-FNTGYSFWGGNLTLAVI 335
KAJ6788989.1      VMSDWQAQHSGVS--------------NAVAGMDMAMPGDTI-FNTGRSYWGGNLTLAVL 337
                   : :                           * *                    :
```

```
KAJ6787877.1      SGRLSRAQFDEATKRIAAMRSKL-----------------WA------------------ 370
KAJ6782998.1      RGELGRDEFRQATERIIALRKV-------------------------------------- 369
KFG78085.1        NGIITKDRIFTSIRRVQHLKSTCT---------------SWAKALN-------------P 370
KAJ6788372.1      NGIITKERIFTSLRRVLHLKASCT---------------SWSKALN-------------P 363
AGC24356.1        EGQLDKDEIRQSLQRVAQLKDQFL---------------NWDDVLQ-------------Q 349
KAJ6789110.1      KDEISQTRIDESLARLSKLKAKFT---------------NWDRALQ-------------T 343
KAJ6785893.1      SKLTTEATLDKSLGRLYSALIKVGYFNPP----AQYNR---------------------- 476
KAJ6786934.1      TGKPFIHHIDE---RVRAVLKLVKKVSSL--PIE----------EN-------------- 289
KAJ6780833.1      NGTVAEARVSDMATRIVASWYLVGQD--N-FPVPGIGMK------------------NLT 344
KAJ6787866.1      NGSVPESRINDMATRILTTWYQFKQD-EH-FPNPGVGMP-----------------KDVT 363
KAJ6786079.1      SGSVPESRVTDMAMRIIASWYQLKQDDPS-FPAPGIGMP-----------------GNLT 357
KAJ6785739.1      NGSVPMTRLNDMATRIVATWYHMGQDQGF----PATNFHYNTRDEKGPLYPAAWPESPTT 395
KAJ6782204.1      NGSLPVSRLDDMTARIVASWYQLGQDDKSRFDRKGPNFSSWTNDKMGYINDGTSSRQEKV 461
KFG84234.1        NGTVPAYRIDDMAMRIMASFFKVGMKPGK---QVPTSFSSWTRDTFGYRQAAAKENWEQV 392
KAJ6788989.1      NGTVPAYRVDDMAMRIMASLFKVGWNVET---AKDTNFFSWSRNTYDYRHAAAKESMEQV 394
                          .     *:

KAJ6787877.1      ------------------------------------------------------------ 370
KAJ6782998.1      ------------------------------------------------------------ 369
KFG78085.1        PGISLLSQLHPSHVSLSRRAYDESITVIRDKEKMLPLASSMHPGE--------------- 415
KAJ6788372.1      PGISLLSQLHPSHLALSRRAFDESITVIRDKEQLLPLARSMHPGE--------------- 408
AGC24356.1        ADLKTMGSE--AHATLSKELYDRVPTVVTNRKNTLPIRPAQTDK---------------- 391
KAJ6789110.1      STDAALDDINARGAKLAHKAYADAATVVRIDDKILPLSTSASTV---------------- 387
KAJ6785893.1      --LGWADVNTTEAQTLAYNAAVKGMTLLKN-DGTLPLSK--------------------- 512
KAJ6786934.1      --GPENTRDTPETAALLRKIGNESIVLLKNDDNILPFTKEKKTLVLGPNAKEATYHGGGS 347
KAJ6780833.1      EPHELVDARNPESKPILMEGAITGHVLLKNTNNTLPFKGKQK------------------ 386
KAJ6787866.1      KPHKIVDGRDPAAKPVLLDGAVEGHVLVKNTKSTLPLRQ-PK------------------ 404
KAJ6786079.1      VPHRIIEGRDPADLPVILQGAIESHVLVKNEKSTLPLQS-PK------------------ 398
KAJ6785739.1      VINENIDVRADH-DIVARQVAQDAITLLKNDAGLLPLSE-ER------------------ 435
KAJ6782204.1      EVNQYVNAQHNH-SILAREVAVEGTVLLKN-SGILPLTR-SGPRTR-------TKHKEGK 511
KFG84234.1        --NFQVDVRGDH-AAHIRESAAKGTVILKN-TGSLPLNK-PK------------------ 429
KAJ6788989.1      --NFQVDVRANH-ANHIRESGAKGTVILKN-NGALPLNK-PK------------------ 431


KAJ6787877.1      ------------------------------------------------------------ 370
KAJ6782998.1      ------------------------------------------------------------ 369
KFG78085.1        ELLLLTPLVKPLPAS---------------AMTKS------------------------- 435
KAJ6788372.1      ELLLLTPLVKPLPAS---------------SLTKS------------------------- 428
AGC24356.1        -ILFLAA------HV---------------PQTL-------------------------- 403
KAJ6789110.1      -FVSPGP------DF---------------PVGGG------------------------- 400
KAJ6785893.1      -FLSNVAIIGPWANVTDKMQGNYAGTAPLL-----------------VNPLDVFQKRWQN 554
KAJ6786934.1      AALRAYYAVTPYEGI-----GEKLGTQPTFTVGAYTHRFLPILGNQLTDPNGKQGMRWRV 402
KAJ6780833.1      -ML---------SVF-----GYDAALPETKNTD----------------------ILFQL 409
KAJ6787866.1      -MV---------SLF-----GYSAKSPDFFAPGGNLQL-----------------LQWQL 432
KAJ6786079.1      -KV---------SIF-----GYSASTPPKWTAQDDT-D-----------------GTWRF 425
KAJ6785739.1      -SI---------KVF-----GTDAAKNP-------------------------------- 448
KAJ6782204.1      VNI---------GIF-----GDDAGP-G-------------------------------- 524
KFG84234.1        -FL---------AVI-----GEDAGQNS-------------------------------- 442
KAJ6788989.1      -FL---------AVI-----GEDAGPNP-------------------------------- 444


KAJ6787877.1      ------------------------------------------------------------ 370
KAJ6782998.1      ------------------------------------------------------------ 369
KFG78085.1        -----------------------------LLESKKTWSQAPTQH---------------D 451
KAJ6788372.1      -----------------------------LLDAKNDQSAATGPH---------------E 444
AGC24356.1        -----------------------------AVDSE-----KEP------------------ 411
KAJ6789110.1      -----------------------------AVNSG---DAAHPTR---------------V 413
KAJ6785893.1      VKYVQG------------------------------------AD--IDSGNTG------- 569
KAJ6786934.1      FNEVPGTADRKLIDELQFTKTEMHLVDYSNPNVNDVWYADMEGSLVADEDCTYEIGVVVS 462
KAJ6780833.1      GYYS----------------------------SQEMAQAVLGTEAHFDQAARG--GTIIS 439
KAJ6787866.1      GAES--------------------------IDLNEVLAGALGNAANASSIGIN--GTLFG 464
KAJ6786079.1      GLAP--------------------------V---------FEEDPATSPTGGR--GTMFG 448
KAJ6785739.1      ----------------------------------------DGPNACADRNCNK--GTLGQ 466
KAJ6782204.1      ----------------------------------------LGPNHCADRGCNE--GTLGS 542
KFG84234.1        ----------------------------------------RGPNGCDDRGCDD--GTLAM 460
KAJ6788989.1      ----------------------------------------KGPNGCADRGCDD--GTLAM 462


KAJ6787877.1      ------------------------------------------------------------ 370
KAJ6782998.1      ------------------------------------------------------------ 369
KFG78085.1        SWAHKDRE-------RSAIMSGEGVF---------------------------------- 470
KAJ6788372.1      RWAHRRLD-------RTAHMSGEGVFREFGKTLARYRNEKLLHTSY-------------- 483
AGC24356.1        -------------------------FNSFHASLLK-RHTNLEYIIY-------------- 431
KAJ6789110.1      SWV----------------------SSSFGDEIRK-HCAGAEEIRY-------------- 436
KAJ6785893.1      GFGAA------------------------------------------------------- 574
KAJ6786934.1      GTAKAFVNDQLIVDNATNQVAGDAFFGSATREERG-RVEMKKGETYNFRIEFGSAPTYTL 521
KAJ6780833.1      GGRAAATAPSYIIDPLNAI---------------Q-QRAMEDDTWV-------------- 469
```

```
KAJ6787866.1    GCGSGATTPANAVSPLEAI---------------K-ARTYRDGTAL-------------- 494
KAJ6786079.1    AGGSGAITPQDSVSPQQAF---------------V-ARAAKDGFTL-------------- 478
KAJ6785739.1    GWGSGTVDYTYLDDPIGAL---------------Q-ARVKNVTL-Y-------------- 495
KAJ6782204.1    GWGSGAVDFPYLVTPESSL---------------S-ATFDLNAVNISSSL---------- 576
KFG84234.1      LWGSGTSQFPYLITPDSAL---------------Q-RQAIQDGSRYESVL---------- 494
KAJ6788989.1    LWGSGTSNFPYLITPDSAL---------------Q-QQAIKDGSRYESVL---------- 496


KAJ6787877.1    ------------------------------------------------------------ 370
KAJ6782998.1    ------------------------------------------------------------ 369
KFG78085.1      -------------------------------LHENLIARASCIIIVTADANRNMYQAGF- 498
KAJ6788372.1    ---------------------TANGV---RPVHENLINRASCIVIFTADANRNLYQAGF- 518
AGC24356.1      ---------------------NEET-----PDLSQKIQEADWVIIGTANANLYPFQVRM- 464
KAJ6789110.1    ---------------------TEDPL---TDEQWSKIEGADVLILATRNAREAPYQQKL- 471
KAJ6785893.1    -----------------------------LDAARSSDHIIYLGGIDIDVENEGH------ 599
KAJ6786934.1    KGDAYVPGHGSLRVGGCKVIDDQEEIKKSVQLAKEHDQVIICAGLNADWETEGA------ 575
KAJ6780833.1    ---------------------NWDTKSFDPPVNPA---SNACLVFINAISTEGWDRDGL- 504
KAJ6787866.1    ---------------------FHDLASPSPVVDPA---SDVCLVLGNAFACEAYDRPAL- 529
KAJ6786079.1    ---------------------VQDLTSPQPQVDPA---SDVCVVFGNAWAREGNDRPQL- 513
KAJ6785739.1    ---------------------NTDSVPPSLPKPAD---DDIAIVFISSDAGENTYTVEGN 531
KAJ6782204.1    --------------------SNKK---PSKDLLQA---MDVCIVFANADAGEGFIRWESV 610
KFG84234.1      --------------------SNYQWAATQRVVAQP---NVTAVVFVNADSGEGYIEVDGN 531
KAJ6788989.1    --------------------SNYAWPATHDLVSQP---NVTAIVFANSNAGEGYINVGGN 533


KAJ6787877.1    ------------------------------------------------------------ 370
KAJ6782998.1    ------------------------------------------------------------ 369
KFG78085.1      --------------TKHVDMMCSMHRSRGNKKQLIVVAVSSPYD---FAMDKSIGTYICT 541
KAJ6788372.1    --------------TKHVEMMCSMLRSRGQKKHLIVVAVSSPYD---FAMDKSIGTYICT 561
AGC24356.1      --------------VQ---------QAQKLAKRLVVAAVMNPYD---QMCFPQVDTYLVT 498
KAJ6789110.1    --------------GLD-------IAQRRSGKTVISIATCAPYD---FLEEPAIKNVIVV 507
KAJ6785893.1    --DRT--SISWPGNQLDLISQLSNLGK----PLVIVKFGGGQVDDSSLLSNSKVNSIVWA 651
KAJ6786934.1    --DRA--SMKLPGVLDQLIAEVAAANP----NTVVVMQTGTPEEMP-WLS--QTKAVLQA 624
KAJ6780833.1    ----------HDDFSDGLVRNVASKCA----NTIVVVHASGIRLVDQWIEHPNVTAAIIA 550
KAJ6787866.1    ----------QDAYTDDLVKTVAASCA----RTVVVLHNAGPRVVDAWIEHDNVTAVIFA 575
KAJ6786079.1    ----------EDAYTDTMINAVADRCA----KTVVVFHNAGPRLVDGFVNHPNVTAVIFG 559
KAJ6785739.1    HGDRDASKLYAWHNGDQLVQDVAKKYR----NVIVVVHTVGPLVLEKWIELPTVKSVLIA 587
KAJ6782204.1    AGDRN--DLFLQRGGDYLVLAVAEECGGGKGKTIVVVHAVGPVVMERWIDHPGIGAVLAA 668
KFG84234.1      AGDRK--NLTLWKNGDDLIKNVSSICH----NTIVVIHSVGPVLVTDWYQNPNISAIVWA 585
KAJ6788989.1    EGDRN--NLTLWKNGDDLIRNVSALCR----NTIVVIHSGGPVLLDEISKNENVTAIVWA 587


KAJ6787877.1    ------------------------------------------------------------ 370
KAJ6782998.1    ------------------------------------------------------------ 369
KFG78085.1      FDFTEDAMSALVRVLVGEITPIGSMPGTLRKSKKVLK----------------SRQHWLV 585
KAJ6788372.1    FDFTENAMASLVRVLIGEVKPVGSLPGTLRKSKKVLK----------------SRQLWLV 605
AGC24356.1      YEYTPPAHEAAVRLIFGEIETRSRLPISIPNVDDAIA----------------P-ATFIV 541
KAJ6789110.1    YEPTLEAFRSAVDIIYGVDTPRGKLPVAEKKV---------------------------- 539
KAJ6785893.1    GYPGQEGGNAVFDVLTGAAPPAGRLPITQYPKSYVENNNILDMNLRPSN-------GIPG 704
KAJ6786934.1    WYGGNETGNCIADTIFGDSNPSGKLSLSFPKR-LQDTPAFLNFR------TEAGRTLYGE 677
KAJ6780833.1    HVPGQDSGRAIVKLLYGEADFSGKLPYTIAKN-ESDYAVYK-PCGLAHKGDTDPQCDYTE 608
KAJ6787866.1    HLPGQDSGAALVSLLYGDAVPSGKLPYTLAKR-DTDYPVLK-PSRPEGRFVNFPQSNFTE 633
KAJ6786079.1    HLPGQAAGDAIVQLLFGDANPSGKLPYTVAKQ-AADYGPLLDPQASPG--SQSPQAYFPQ 616
KAJ6785739.1    HLPGQEAGRSLVEVLYGDVSPNGHLPYSITHA-EEDMPKSVTDLINFELAGKQADDDYTE 646
KAJ6782204.1    NLPGQESGNAISTIIFGDASPSGKLPYTIGKS-LEDYGPGAKVMYTPN--GLVPQQDFNE 725
KFG84234.1      GLPGQESGNSITDILYGKTSP-GRSPFTWGPT-RESYGTD--VLYKPNNGNGAPQQDFTE 641
KAJ6788989.1    GQPGQESGNSLTDILYGKTSP-GRTPFTWGAS-EAAYGND--VLYKPNNGKGAPQDDFTD 643


KAJ6787877.1    ------------------------------------------------------------ 370
KAJ6782998.1    ------------------------------------------------------------ 369
KFG78085.1      EEYDRARDYAGLEDLLKAVHRASAPDLHFLKSTRAASMELHLPNIKETHFV--------- 636
KAJ6788372.1    EEYDRERDATGLDDLLKAIHRSGGSDLDFLANTSASAFELFNTNIKEAHYV--------- 656
AGC24356.1      DDYRNDDDLDHVTAMWDDIFG---KDWPLR----KDKINLGLQRAKLQKHK--------- 585
KAJ6789110.1    ------------------------------------------------------------ 539
KAJ6785893.1    RT---YMWY---T---------GKPILPFGYGLHYTNFSTSWKTKPSASYSIQALA---- 745
KAJ6786934.1    DVYIGYRYYEFAD---------REVNFPFGHGLSYTTFGFSDLSVNVADGKI-------- 720
KAJ6780833.1    GQYLDYRAFDAKN---------ITPRFEFGYGLSYTTFNYSYLSISAHG----------- 648
KAJ6787866.1    GVFTDYRHFDQKN---------ITPRFEFGFGLSYTTFGLANLQARR-FDNASDALA--- 680
KAJ6786079.1    GVYTDYKYFEQAH---------IEPRYEFGFGLSYTRFDYSDIVLGARPNAPTTPPP--- 664
KAJ6785739.1    GLYIDYRWLNKQK---------IKPRYAFGHGLSYTKFNLSDASIKKVTSLTKT-PPARA 696
KAJ6782204.1    GVYVDYRHFDKFN---------IQPRFEFGFGLSYTTFNYSNLQLTEVKAKSRLAGERPA 776
KFG84234.1      GSFIDYRHFDKVAPG-KNS--TGAPIYEFGHGLSWSTFEYSNLKVEKRDVRP--MSPPNG 696
KAJ6788989.1    GAFFDYRHFDKESDG-KPGSDPKAPVYEFGFGLSWSTFEYSNLQVVKKNDRP--YKPTTG 700


KAJ6787877.1    ------------------------------------------------------------ 370
KAJ6782998.1    ------------------------------------------------------------ 369
```

```
KFG78085.1      ------------------VRNSSTGALYGFVATYFI----AGVG---------------- 658
KAJ6788372.1    ------------------VRNSSTQALYGFVATYAT----GGVG---------------- 678
AGC24356.1      ------------------VARDSQGKIVGFVATQIVVVDNKKHG---------------- 611
KAJ6789110.1    ------------------------------------------------------------ 539
KAJ6785893.1    ------------------------------------------------------------ 745
KAJ6786934.1    ------------------------------------------------------------ 720
KAJ6780833.1    ------------------------------------------------------------ 648
KAJ6787866.1    ------------------------------------------------------------ 680
KAJ6786079.1    ------------------------------------------------------------ 664
KAJ6785739.1    AKSGILDYSQKIPDASEGVAPDGFKKLFRYIYSWVQPNEAADAVK-----DAATKKYPYP 751
KAJ6782204.1    DQANPPDFEDKIPDISEAIFPSEIRKLKKFIYPYITSASDIKPG--VYPY---------P 825
KFG84234.1      KTIAAPTFGNFSTNLKDYAFPPSIRYIYQFTYPWLNTTASGKEASGDPHYGQTAEQFLPP 756
KAJ6788989.1    NSIAAPTFGNFSKDIKDYGFPAGFHYVKQFIYPWLNTTSSGKEASGDSSYGKTADEFLPP 760

KAJ6787877.1    ------------------------------------------------------------ 370
KAJ6782998.1    ------------------------------------------------------------ 369
KFG78085.1      ---------------------II-------------------------GALLVDPSKRNV 672
KAJ6788372.1    ---------------------IL-------------------------GALFVDPTKRNV 692
AGC24356.1      ---------------------QL-------------------------MLLMVSPSYQGK 625
KAJ6789110.1    ------------------------------------------------------------ 539
KAJ6785893.1    ------------SQAGNDPNNALFAN---LAISVKNTGGEANIASDYVGLLFLSSQNAG- 789
KAJ6786934.1    ----------------------------IAIVTVTNTGS--R-AGSAVAQMYIEPKQAA- 748
KAJ6780833.1    -----------VRKCVSTTLNELWEVVATLEVSITNSG---AVAGHEVAQLYLGIPGA-- 692
KAJ6787866.1    --------EYPTGAVIPGGHADLWDNLVSVTADVTNTGR--NHTGAEVVQLYLGIPSGG- 729
KAJ6786079.1    --------EYPSGEIVSGGQKDLWEVLATVKCRLVNGG---KVAGAEAVQLYVRFPGM-- 711
KAJ6785739.1    DGYSTEQKPGPRAGGGEGGNPALWDVAYTLSVKVTNVGN--KYGGKASVQAYLQFPDDA- 808
KAJ6782204.1    DG-YDVKQPPSAAGGGQGGHPELWETHVSVQADVGNEGA--V-GGKVVAQLYISFPNTK- 880
KFG84234.1      GATDGSPQPRLASSGEPGGNRQLWDVVYTVSATITNTGK--R-VSDEIPQLYISLG---- 809
KAJ6788989.1    NAIDANPQPKHPAGGEPGGNRQLFDVIYEVTATITNTGK--T-MTDEVPQLYLSHG---- 813

KAJ6787877.1    ------------------------------------------------------------ 370
KAJ6782998.1    ------------------------------------------------------------ 369
KFG78085.1      SIGRSLHRRALKSLSQQRGLKKVQVGMSFPGVFLGIPLDPE-ANTVKEWFSNSGWDTQFP 731
KAJ6788372.1    SIGRSLHKRALKTLSQQRGIKRVQLGACFPSVFPGIALGVE-AATVRTWFSNSGWDTQFP 751
AGC24356.1      GVGTLLHDAALEHFR-EQGADCIKLGSTYPRFFPGVPDDDAQSRKAQAFFSKKGWRMD-- 682
KAJ6789110.1    ------------------------------------------------------------ 539
KAJ6785893.1    --PS---PRPNKKLVSYGRAQSIQVGSTQQLNL-RI-----------NIAELARSDEN-- 830
KAJ6786934.1    --RI---NRPVKELRGFAKVE-LEAGASKTVTISEL-----------EKYAAAYFDEE-- 789
KAJ6780833.1    ---------PPKQLRGFEKLG-LQPRETGKATF-EL-----------TKRDLSQWDVV-- 728
KAJ6787866.1    --GAAAAAPPVRQLRGYEKVT-LQPGETKTASF-AL-----------SRRDLSVWNVE-- 772
KAJ6786079.1    ---------QAKQLRGFEKPT-LKPGESRSVSF-EL-----------TRRELSVWNTE-- 747
KAJ6785739.1    --GY---ETPIIQLRDFEKTKTIAPGGSETVKL-QL-----------TRKDLSVWDTV-- 849
KAJ6782204.1    --GV---DFPVRALRGFEKVY-LEPGQTKSVQF-NL-----------TRRDLSYWDTT-- 920
KFG84234.1      --GK---NEPVRVLRGFNRIEGIAPGQNVTFRH-EI-----------TRREISNWDTA-- 850
KAJ6788989.1    --GE---GEPVRVLRGFDRIERIAPGASATFRV-EL-----------TRRDISNWDSN-- 854

KAJ6787877.1    ------------------------------------------------------------ 370
KAJ6782998.1    ------------------------------------------------------------ 369
KFG78085.1      RRLTNMIIHNLPNWVAPEGLLQSIQRANISFDLIHGLENAESVLHHVRANANPEVLELYT 791
KAJ6788372.1    RRLTNMIIADLGNWTAPEGLLPTIQRANISFDLIHGLDNADTVLHHVRTESGPEVVELYR 811
AGC24356.1      DNLVHDLIGDLQDYKVPDKIQARMLKEKIWFGRIKPSETWELYAF--QQRNFPHWLSTYQ 740
KAJ6789110.1    ------------------------------------------------------------ 539
KAJ6785893.1    ----------GDKYIYP------------------------------------------- 837
KAJ6786934.1    ----------RDQWCVES------------------------------------------ 797
KAJ6780833.1    ----------QQKWVLHA------------------------------------------ 736
KAJ6787866.1    ----------RQKWHLQP------------------------------------------ 780
KAJ6786079.1    ----------KQMWQLQR------------------------------------------ 755
KAJ6785739.1    ----------KQDWVIPTVD---------------------------------------- 859
KAJ6782204.1    ----------LQNWVMPE------------------------------------------ 928
KFG84234.1      ----------SQNWVITK------------------------------------------ 858
KAJ6788989.1    ----------AQNWVVTD------------------------------------------ 862

KAJ6787877.1    ------------------------------------------------------------ 370
KAJ6782998.1    ------------------------------------------------------------ 369
KFG78085.1      YA--LSENKTCGIVRAKDPSGNLIGTVII----CRQGSPLVTYIPPLLSD-----GEDVG 840
KAJ6788372.1    YA--LAETKSCGIVRAKDPAGAIMGTIII----CRQGSPLSTYIPALRSR-----SENVA 860
AGC24356.1      HHVELGDYQDLIVARQDDENGRVIASLIL----NTTHVS-HEYRSDLIWTDDKLFGERSG 795
KAJ6789110.1    ------------------------------------------------------------ 539
KAJ6785893.1    -----GDYTLTLDTTGATAA-----------QFKLTGQPAKI--STLPRK---------- 869
KAJ6786934.1    -----GEYDVVISDSSAVTEKALRATINVSESYWWSGI---------------------- 830
KAJ6780833.1    -----GEYKIYVGASSRDIR--LTGKIVISG----------------------------- 760
KAJ6787866.1    -----GTYTVWVGTSSRNLP--LKQTLKI------------------------------- 802
KAJ6786079.1    -----GDYEIYIGRSSSKLP--LKTYLTI------------------------------- 777
KAJ6785739.1    -----GGYKVWVGEASDNLG--TVCSIDSMK--CQSGAKGPV--Q--------------- 893
```

```
KAJ6782204.1    -----GQFSIAVGASSRDLP--LTGTW--------------------------------- 948
KFG84234.1      -----APKKVFVGSSSRNLP--LSADLN-------------------------------- 879
KAJ6788989.1    -----APKTIWVGSSSRNLP--LSAKLQ-------------------------------- 883


KAJ6787877.1    ------------------------------------------------------------ 370
KAJ6782998.1    ------------------------------------------------------------ 369
KFG78085.1      GIIAPIVPSTPQATLVLQGLALMGVRQSKGHRASKAVLSWIVDDAFEPLVAMGFDILQAF 900
KAJ6788372.1    GIVAPTVPPGPLSTLVLQGLALMGVRQCKSHKASKAVLSWVIDDTSEPLIGMGFEALQSF 920
AGC24356.1      GMACVGVAQEERGRGIGIGIVAHANWLLKQRGVTKSYVDWVELLDFYS--RVGYKTWRSY 853
KAJ6789110.1    ------------------------------------------------------------ 539
KAJ6785893.1    -------------S---------------------------------------------- 870
KAJ6786934.1    ------------------------------------------------------------ 830
KAJ6780833.1    ------------------------------------------------------------ 760
KAJ6787866.1    ------------------------------------------------------------ 802
KAJ6786079.1    ------------------------------------------------------------ 777
KAJ6785739.1    ------------------------------------------------------------ 893
KAJ6782204.1    ------------------------------------------------------------ 948
KFG84234.1      ------------------------------------------------------------ 879
KAJ6788989.1    ------------------------------------------------------------ 883


KAJ6787877.1    ------------ 370
KAJ6782998.1    ------------ 369
KFG78085.1      EEITNSPEV--- 909
KAJ6788372.1    EEITNSPDIFQN 932
AGC24356.1      RLGH-----F-- 858
KAJ6789110.1    ------------ 539
KAJ6785893.1    ------------ 870
KAJ6786934.1    ------------ 830
KAJ6780833.1    ------------ 760
KAJ6787866.1    ------------ 802
KAJ6786079.1    ------------ 777
KAJ6785739.1    ------------ 893
KAJ6782204.1    ------------ 948
KFG84234.1      ------------ 879
KAJ6788989.1     ------------         883
```

Figure S2. Clustal alignment of *A. album* GH3 enzymes. The twelve sequences were aligned together with the GH3 β-N-acetylhexosaminidases identified in *R. miehei* (AGC24356) and *M. anisopliae* (KFG78085) and with one *M. anisopliae* GH3 β-glucosidases (KFG84234). Boxed sequences correspond to the conserved motif KHF(I)PGH(L)GXXXXDS(T)H, typical of GH3 sequences.

Figure S3

```
KAJ6780494.1     ------------------------------------------------------------ 0
KAJ6784096.1     ------------------------MAS---TQT----FGGKRAMTAGSVRPTANYEKPRA 29
KAJ6785798.1     ------------------------------------------------------------ 0
KAJ6785933.1     ------------------------------------------------------------ 0
KFG80340.1       ------------------------------------------------------------ 0
KAJ6783033.1     ------------------------------------------------------------ 0
KAJ6780076.1     ------------------------------------------------------------ 0
KAJ6789215.1     MYKFVQLFCLFWLFLLTVHALAAGLQDTTTTTTTGIVVARSHAVTVG-FLPVQRFHHLQA 59
KAJ6787434.1     ------------------------------------------------------------ 0
KFG85702.1       ------------------------------------------------------------ 0
KAJ6780083.1     ------------------------------------------------------------ 0


KAJ6780494.1     ------------------------------------------------------------ 0
KAJ6784096.1     RGAEPGARADKES------------------------------------ALHLGQQPGAD 53
KAJ6785798.1     ------------------------------------------------------------ 0
KAJ6785933.1     ------------------------------------------------------------ 0
KFG80340.1       ------------------------------------------------------------ 0
KAJ6783033.1     ------------------------------------------------------------ 0
KAJ6780076.1     ------------------------------------------------------------ 0
KAJ6789215.1     RDQAAGSSCSSEGQWNCMTNSWQRCAAGRWSTTMQCAKGTTCAPAGLTNEFRIQHDGSVN 119
KAJ6787434.1     ------------------------------------------------------------ 0
KFG85702.1       ------------------------------------------------------------ 0
KAJ6780083.1     ------------------------------------------------------------ 0


KAJ6780494.1     ------------------------MLHQQCATALWASI--------------VALSLSAT 22
KAJ6784096.1     GARRPDSEEQEAAAADAAAANRDLTGPAAPTAHLWHRR----RLCRDRWEFAAQWNEAFF 109
KAJ6785798.1     -----------------------------------------------------MWSS--- 4
KAJ6785933.1     ------------------------------------------------------------ 0
KFG80340.1       ------------------------------------------------------------ 0
KAJ6783033.1     ------------------------------------------------------------ 0
KAJ6780076.1     ------------------------------------------------------------ 0
KAJ6789215.1     GNGGPPTTSVA----------------ASLGTRTWVSKVGTGFLVLGWWFLRIKFAIHSK 163
KAJ6787434.1     ------------------------------------------------------------ 0
KFG85702.1       ------------------------------------------------------------ 0
KAJ6780083.1     ------------------------------------------------------------ 0


KAJ6780494.1     ASPI-------V-----GS------SADAAQIDLNGAADSK---SNLPAVWPPPQKISGS 61
KAJ6784096.1     ESDKMHAMVDKIKEIIQELTQDLNRNGLSQI--VLSTLACQGGA---ASLIGIPTADYTT 164
KAJ6785798.1     ----------------------------FNL--LSGLLLLDVAL---AHLPGIPTVPFKG 31
KAJ6785933.1     ------------------------------------------------------------ 0
KFG80340.1       ------------------MR-----FYTSALLAALATVELTSAVTVKVNPLPAPQDVTWG 37
KAJ6783033.1     ------------------MRS---QSLSSALLIWL-AAATGLGAAVKVNPLPAPQEITWG 38
KAJ6780076.1     -------------------------MRL---IVF--STLLA--AA--TALWPVPKELQTG 26
KAJ6789215.1     ESSL---GLPSVDVVFYCFRK--ARSGMHLRIVFVVVASAQ--LA--AALWPVPKHLSTG 214
KAJ6787434.1     --------------------------MLSKFLALLALAAIG--QV--NAIWPIPSSLSTG 30
KFG85702.1       -------------------------MLNNAIIAVAALAALR--PV--DAIWPVPQQISTG 31
KAJ6780083.1     -------------------------MRLHSY--LAVALAAS--PV--AALWPIPIDISTG 29


KAJ6780494.1     GDCAV-----R--L---------------------SSEVTIVTGNATDG----PTIKVIQ 89
KAJ6784096.1     TECK--------RF-------------------DLRQVSGIVVDAQHASARDTQGATLIP 197
KAJ6785798.1     DGKK--------TF-------------------KLDAVQTVIIDSKYTKTTDKSGLTLIP 64
KAJ6785933.1     -------------------------------------------------------MSLIP 5
KFG80340.1       DSGP-----KHVGW-------LNL--RTNSN-RKSESNTQLVSEAWNRASKAITSLRWVP 82
KAJ6783033.1     ESGA-----IPVGY-------LTL--RTVNADWSTNDNVRIVNDAWNRAFKAITTIRWVP 84
KAJ6780076.1     NETVWIGQDIQVSYRHGNNCYVFDFGKNADDSHDEITVRAVVRGAIARALSTVFDEGLIP 86
KAJ6789215.1     QTILEIGTALKITYNGGSLAWNGQAVDSQ-----NLNDTNIVQGGIARAYNAIIESGFVP 269
KAJ6787434.1     SDVLFISQDIQVTFNGGALSSASS----------GGGGRSTIPDAVARTLDTILKKGFTP 80
KFG85702.1       QDVLFVDKSIQVTYNGEPVSYDGRGTETK---ESPSSSSQVVHDAVARSLTAIFEHGLVP 88
KAJ6780083.1     NKTLYIDKTINITYNGEPLQYTTGYNPPA---GSSFTSKSIVQGALARSLKAIFDDGYVP 86


KAJ6780494.1     AIVAAAGGKATVSPYASGAGAQIF-----------VGTDSENRA-------AADAAKALT 131
KAJ6784096.1     PTLQEFAT--TFA--------SDLASSLGIKTNVEVAAAPRDQ---CIYI-TLDP-QGNY 242
```

```
KAJ6785798.1      PTLHEFGA--TFA--------QDLQEVFNKTVTAKDGDKRESN---TIFL-TIDS-DHEF 109
KAJ6785933.1      ----QPQS---VTPKEG-I-----VE-----------------------LASLDQIV--- 26
KFG80340.1        QAIEQPIP--KFEPFPT-AGSNDSSSS-----------AKSKRNTGGSWLNEVNVQVSDW 128
KAJ6783033.1      QAVEQPIP--KFDPFPG-KNSTSNSKRADAQADAQAGDASASNNWGSRWLNEITVKVDDW 141
KAJ6780076.1      WKLVPRNELPNHEPSAD-AT-KTYIRKLSIV---QTAVA--------------ND----- 122
KAJ6789215.1      YMLNPPGS--DFEPSRA-QI-QGQIKVLVIS---NVRK---------------------- 300
KAJ6787434.1      WMLREANS--DYEPAIG-SG-KGKVKSLALK---RAGKE--------------AGGSSSS 119
KFG85702.1        WMLNPPGS--DFEPALD-DG-AAKVTSLTIK---QTGED--------------NATV--- 124
KAJ6780083.1      WMLHKPGS--HFEPRCG-PNNKNHVRSLTIT---QTGTD--------------DAST--- 123


KAJ6780494.1      GDSAKSLTAEGYVLGSGKYNGHPTIVLNGVDTRGTFYAAQTLRQLVGGK----------- 180
KAJ6784096.1      TDAAGRFTHEGYSLTTNSS----GVFIAGASPLGAWWATRTVLQQAAIRSQ-----TKNV 293
KAJ6785798.1      LDAAGRSTTEGYTIVAEED----GVTIRGASSLGAWWGTRTLLQQAILGD---------K 156
KAJ6785933.1      SSLDPTLHPEGYTIDISAA--Q-GVQIKGGSDAGLFYAKQTLRQLIPPSALRSGARGPWR 83
KFG80340.1        SADLKHGVDESYTLTVSAS--SSTVEIAAKTVWGALHAFTTFQQLVIFEG-------GSL 179
KAJ6783033.1      SADLKHGVDESYTVDITSS--SSQVQVTAKTAWGALHAFTTLQQMVISDG------RGGL 193
KAJ6780076.1      -SSLIGPSNESYSLHIETD--G-TASITAVAPSGILRALQTFTQLFYLHS----TKQGSY 174
KAJ6789215.1      ------NNSESYSLHVDGS----NAVINAESTIAVLRALETFTQLFNQHS----SGSYWY 346
KAJ6787434.1      SRLDFAEMDESYTLDLSSS--G-DATIAANSTVGLLRGLETFTQLFYKHS----SGAAVY 172
KFG85702.1        FKPLAGQVDESYSLHLEAN--G-EASIEAVTSTGLVRGLESFTQLFFKHS----SGDAFY 177
KAJ6780083.1      FKPLAGQRDESYALNVTFD--G-LASIRANSSIGVMRGLETFSQLFFKHS----SGGAWY 176
                           *.* :            : .    .   .  :. *

KAJ6780494.1      RGVPGVKVRDWPLMPVRGSIEGFYGIPWSHQARMDQYVFYGQHKMNTYVYTPKGDPY--- 237
KAJ6784096.1      TALPYGHGRDAPGWGMRGMMLDAGRHWYPKEFLVEMCGYMSYFKQNTFHVHLSDNLVTQD 353
KAJ6785798.1      SAVPVGQGDDAPGWSTRGMMLDCGRHFYPKQFLIDMCSYISFFKQNTFHIHLSDNTIVPT 216
KAJ6785933.1      LPAQTISDAPSSGLRWRGCMLDVARHFMPVSDVLHFIDVLAFHKLNTFHFHLTDDQGWRI 143
KFG80340.1        IVEQPVTIKDHPNYPYRGVMVDTGRNFISANKIKEQIDGLALSKMNILHWHITDTQSWPI 239
KAJ6783033.1      IVEQPVKIKDHPNYPYRGVMVDSGRNFISVNKLREQIDGLALSKMNILHWHITDDQSWPI 253
KAJ6780076.1      TRLAPIHITDAPQFSHRGINLDVARNFYPVKDVLRTIDALAWNKFNVLHLHMTDSQSWPM 234
KAJ6789215.1      TTLAPVAIEDSPTFPHRGLIFDVSRERYSVDDVKHTIDALAMAKLNKLHLSVTNTQSWQV 406
KAJ6787434.1      TDMAPVSIRDAPVMPYRALLIDVARHWYEVADIKRTIDGLAMNKLNVLHLHITDTQSWPL 232
KFG85702.1        TKQAPVSIQDAPRFPHRGLVLDLSRHWFAVDDIKRTIDGLAMNKMNVIHLHITNTQSWPL 237
KAJ6780083.1      TTQAPVRIADAPKFPHRGMMLDVSRHWFAVADIKRTIDGLSMAKMNVLHLHITDTQSWPL 236
                                  *.   .                  .  * *      ..

KAJ6780494.1      LRATWRELYGGEDLDKLKELIETANANHVDFTYALSPGLDICYTSGADFNATVAKFEQLR 297
KAJ6784096.1      YYSYEQAMALEANFRLF---SESG---ALQ---------GLSSPRDV--AYTRADFDEVQ 396
KAJ6785798.1      YTPD-NYNETYARFRLW---SDSS---EVS---------GLNKHANE--SYTKDDFEDIQ 258
KAJ6785933.1      DVPQWPRLAEVASSRKR---SMQGTRHDCS---------FDARPHGG--FYTADDLREIV 189
KFG80340.1        HLEAYPQVT-KD-----------------------------AYSGRE--SYSAKDVQDII 267
KAJ6783033.1      HIDAFPEFT-KD-----------------------------AYSERE--IYSANDVADVI 281
KAJ6780076.1      EIPSLPDLAGKG-----------------------------AYYAGM--SYTPTDIETIQ 263
KAJ6789215.1      EIPSFPELTEKG-----------------------------TFYSGQ--YYSVDDLHDLY 435
KAJ6787434.1      EIPALPLLAEKH-----------------------------RYAKGL--TYSPADLEDIQ 261
KFG85702.1        EIPALPKLAEKG-----------------------------RYAPGL--TYSPQAIQEIQ 266
KAJ6780083.1      EIPALPLLAERH-----------------------------AYSKGL--TYSPADIADLH 265
                                                                     :   .  :

KAJ6780494.1      KLGVSSFYIALDDIPLEFHCDSDKQKYPNTGNY---HWL--------------ADAHAFY 340
KAJ6784096.1      QKCAARGVTVMPEIEAPGHALRIVQWKPQLG----------------------FHKDPSL 434
KAJ6785798.1      TKCAQRGVTIIPEIEAPGHCLPIVQWRPQIG----------------------YQGDLSL 296
KAJ6785933.1      AFAAERHVTVVPEVNMPGHMQAAIAAYPELGNTDVEGRSPDAGVWDSWGI----SPR--V 243
KFG80340.1        SYARARGVRVIPEIDMPGHSASGWQQIDKDIVTCQNSWWS----NDNWPLHTAVQPNPGQ 323
KAJ6783033.1      SYARARGVRVVPEIDMPGHSASGWQQYDKDIITCENSWWS----NDNWPLHTAVQPNPGQ 337
KAJ6780076.1      KYALHRGVTVHIEFDMPGHTTAVSWGYPELVAAAEAQPR---------G-RYCAEPPCGT 313
KAJ6789215.1      TYAVSRGVEIVMGIDMPGHSN-IQHIYPDLGVAYMAHPW---------N-TYCSEPPCGS 484
KAJ6787434.1      EYGAARGVQVILEIDMPGHVG-IERAYPGLSNAFAAQPW---------G-TYCAEPPCGS 310
KFG85702.1        EYGVARGVQVLLEIDMPGHVG-IDKAYPGLSVAYNEKPY---------D-KYCAQPPCGA 315
KAJ6780083.1      EYGIHRGVQVILEIDMPGHFG-IERAYPGLSVAYNKRPY---------G-QYCAQPPCGS 314
                               .    *

KAJ6780494.1      LNRVQKEYIKAHEGLT-DL-ETVP--TNYAGSAPDPYKSEFGKQLDKNIRVQ-------- 388
KAJ6784096.1      LNISHPETIPTMKSIWQEFLPWFH--TKLVSIGADEYHGPV-ADYNR------------- 478
KAJ6785798.1      LNISHPDTIPTMKTIWKEFLPWFH--TSVVSIGADEYKGPE-EDYKR------------- 340
KAJ6785933.1      LNV-SDKTLDFCRSVLDLVCDIFP--SETVCIGGDECPYDE-WKASPSVQARMKELGIKT 299
KFG80340.1        LDVMNPKTYQVVGNVYSELSKKFS--DDFFHVGGDELQIGC-FNFSKGIRDWFAADPKRT 380
KAJ6783033.1      LDVLNPKTYKTVEKVYAELSHRFS--DDFFHVGGDELQTGC-FNFSKPIRDWFAEDSSRT 394
```

```
KAJ6780076.1    LQLNNPAVDDFLDTLFDDVMPRVKSYAAYFHTGGDEVNAQA-YTLDPTMQT----NDRSK 368
KAJ6789215.1    YRLNNTGVDRFLEKLFEDLLPRIAPYASHFHTGGDEYNANN-CALDPDIRS----GEMSV 539
KAJ6787434.1    LKLNNTDVYKFLDTLFDDLLPRTAPYSSYFHTGGDEYKASN-SLLDPALRT----DDTSV 365
KFG85702.1      LKLNNTDVENFISTLFDDLLPRLSPYSAYFHTGGDEYKATN-SLLDPDLQT----DNMTL 370
KAJ6780083.1    LRLGNKKVEEFLDTLFEDLLPRLSPYTAYFHTGGDEYKANN-SLLDSDLQT----NDPAI 369
                              :   .             . *          .

KAJ6780494.1    -WTGEGVFSDKITVDSVVRADSTYVTDKLFIWDNFPVN-DNKPFRL-------------F 433
KAJ6784096.1    -------FVNTMND-FIR----EQSNKTMSIWGTFPPK-YDGTYDNINQNVAIQHWEFFE 525
KAJ6785798.1    -------FVNEMSA-FMN----EEASKSIRIWGTFPAVKHAGSPTTIDSNITIQHWAYLF 388
KAJ6785933.1    ESALQGWFTDRMA------AHLATRGKRAYGWDEVIEG-YTGP----TEKILVAAWRG-- 346
KFG80340.1      YFDLNQYWIDHAYPLFMSEENSGKKDRRLIMWEDVVLS-PDAHAHNVSKSVIMQSWNNG- 438
KAJ6783033.1    YFDLNQHWIDTSMPIFTSEKNTGKKNRRLIMWEDVVLS-PDAAAHNVSKDVIMQSWNNG- 452
KAJ6780076.1    IRAQIQKMVDRNH------AHVRKAGLTPIVWEEMLLE-WNLT--L-PLDVVVQTWRG-- 416
KAJ6789215.1    VQPLMQKFVQNVH------KIIRKRGLTPVVWEETINV-WNVT--L-PKDTLVQSWRG-- 587
KAJ6787434.1    LKPLLHAFLQHVH------GKIRQHGMTPFVWEEMVGQ-WGAP--LPTNDTIIQTWFG-- 414
KFG85702.1      LQPLLQRFLDHAH------NNIRGHGLVPIVWEEMVEE-WAAD--V-GNDTVVQAWLG-- 418
KAJ6780083.1    LQPLLQRFLDHAH------GKIRELGLVPMVWEEMINE-WNAT--V-GKDVVVQSWFG-- 417
                         :                     *

KAJ6780494.1    LNPLTERATDLYKHMIGFTSNPMVQSYASMIALGNYGD---------------------- 471
KAJ6784096.1    DKPLTDYIQNNYSVINSDDTY-----YVVNKYSESYPPALPLDKTFSADPSVPGGGP--- 577
KAJ6785798.1    DNPYEDYIKNNYSVINSDEMY-----YIVMKDG-PYGRKINTDITFTGN--PDGHGP--- 437
KAJ6785933.1    PGPTAIAARRGFDVVTCPDVN-----AYLDYRQSD-----D-----PGEPTPVGI----- 386
KFG80340.1      VANIDKLTKAGYDVIVSSADF-----MYLDCGNGGYVTNDPRYNAPQSNPDATGATFSFN 493
KAJ6783033.1    ITNIGKLTEQGYDVIVSSADF-----LYLDCGFGGYVTNDPRYNV-QENPDPTGATPSFN 506
KAJ6780076.1    DAAIAETVKQGYKALVGNKAS-----WYLNCGMGSWYNAAN--------------GAEFK 457
KAJ6789215.1    GAT-PSLAKAGYKVIDSSSSY-----YYLNCGRGAFVDYQD--------------GSSLQ 627
KAJ6787434.1    AESVQKYARAGYRVIDTSYDA-----YYLDCGRGSWVDYAD--------------SAV-S 454
KFG85702.1      SASVAKLAAAGHKVIDSTFDI-----YYLDCGRGQWLDFKD--------------GPSLD 459
KAJ6780083.1    QDKVKELAEAGHKVIVSSSDS-----YYLDCGRGEFIDYEN--------------GPDFQ 458
                           .  :

KAJ6780494.1    -----YSWNSPAYNPQDSWAAVI-----------QELAGKDQGIHSALLA----FADINQ 511
KAJ6784096.1    -------WYPHVFNAKKA--------SDNPQRSEPRVLGSIVPLWNDFGQNATVASEAYY 622
KAJ6785798.1    -------WYPNIFNLSNP--------NSNPSKNNKLIQGAISPLWNDHGANTSVYSEAYY 482
KAJ6785933.1    -----------LLTLENVYSFEPVPAGLTPEE-ARHIVGAQVNVWVEHMESARRVQ--YM 432
KFG80340.1      YGGPGGSWCAPYKTWQRIYDYDFTA-NLTSDQ-AKHIIGAAAPLWSEQVDDA-VIS--SK 548
KAJ6783033.1    YGGIGGSWCAPYKTWQRIYDYDFTQ-GLSEAQ-AKHIIGASAPLWSEQVDDT-IIS--GK 561
KAJ6780076.1    QQYPFNDYCSPVKNWRLVYAFDPLA-GVPANL-TDLVLGGEVHLWSEQADGV-NVD--GL 512
KAJ6789215.1    NAYPFTDWCAPAKNWRVVYMHTPTD-GVDARH-AQNVVGGEVAIFSETVDAA-SLD--VI 682
KAJ6787434.1    PGTTFNSWCDPFKNWHIVYNHDPFA-GLEGSAAAKKVVGGEVALWSETIDGA-TFD--QI 510
KFG85702.1      AAYPFADYCSPTKNWRLIYSHDPVE-NMTAEA-AANVIGGEVAVWTEMIDPV-SLD--TL 514
KAJ6780083.1    GAYPFTDWCAPTKNWRLIYAQDPRA-GVSEEA-AENVIGGELPLWTETIDTT-SLD--SI 513
                             .                      : *    :

KAJ6780494.1    NWPYRNNTESAPQLSKDIDAFWAARKSSQKQSDSALASRLTLIA-SIPDLLPG------- 563
KAJ6784096.1    AWREG--------LPAVADKQWGGTLNRAQFDAVFPQVQPYAPGQNLDRAIPSPAGETIL 674
KAJ6785798.1    AWRDG--------IPALADKHWGGNLTQDQYSNWFSKLLAKIPAQTLERRIPSNQ-NTIF 533
KAJ6785933.1    VYPR---------ACAFAEVAWGKTKEHGSF----EDFRKRLEDKHLARLD-------AL 472
KFG80340.1      MWPR---------AAALAELVWSGNKDPKTGLKRTTYLTQRILN---------------- 583
KAJ6783033.1    MWPR---------AAALGELTWSGNKDPKTGKKRTTTFTQRILN---------------- 596
KAJ6780076.1    LWPR---------AAAAAEVLWSGRHDAGGQNRSQVDAAPRLAE---------------- 547
KAJ6789215.1    LWPR---------AAAAGEIWWSGRKDENGTDRSVWHARPRLSE---------------- 717
KAJ6787434.1    AWPR---------ASAGGEIWWSGRKDASGNLRSQYHARPRLAE---------------- 545
KFG85702.1      AWPR---------AAAAGEAWWSGRKDGEGNLRSVFTARPRLEE---------------- 549
KAJ6780083.1    VWPR---------AAAAGESWWSGRYDADGKNRSMYDARPRLSE---------------- 548
                 :                 :  *.

KAJ6780494.1    -MAMKPF----AADVAPWSTVAMQWAKASQHMISMIAAIDAGDKST-ADSEY-------- 609
KAJ6784096.1    QYNFASIPGHVVKDASGNGHDAKTTCMTDRTALAIRPGCDVTLPFGAKGRDYTLSLTVKL 734
KAJ6785798.1    SYDLRKVKGDSVADKSGNKYDAKTTCKGADDGIAMSTDCDITTPWSSKGRNYKLSLTLNM 593
KAJ6785933.1    HVNYRP---QTGPRPWDGRPDAPGYPQSREHR-----LAEIEKM----------TAELRL 514
KFG80340.1      ---FRE---YLVANGIGAAPLVPKYCLQHPH------ACDLYYN----------QTAVQ- 620
KAJ6783033.1    ---FRE---YLVANGIGATPLVPKYCFQHPH------ACDLYYD----------QEAVK- 633
KAJ6780076.1    ---MRE---RMVLGGVQAGPVQMVFCTQKDAR-----ECIF------------------- 577
KAJ6789215.1    ---MRH---RMLARGVKGATVTHTWCEQHALE-----ECTSGYS----------G----- 751
KAJ6787434.1    ---MRE---RMLARGIVGGTIAPGFCLQSELG-----ACV-------------------- 574
KFG85702.1      ---MRE---RMLARGVRGAVISQLFCGQSPLE-----DCAA------------------- 579
```

```
KAJ6780083.1    ---MRE---RMLARGVRGAPITQLWCDQADTI-----DCSSE------------------ 579


KAJ6780494.1    ---------------------------------------NAAQEWVEKTKAKTVSSLRDD 630
KAJ6784096.1    DQDMRGQNATILTGTDSALLLTPTLTLYASNNYYPLNLTIPVNRWVDI-------TIRGK 787
KAJ6785798.1    DKLTDETNTTLVAGSDSTLMLTPNVTLFASGIYYRLNKTIPLQSWVRL-------EINGD 646
KAJ6785933.1    ------------------------------------------------------------ 514
KFG80340.1      ------------------------------------------------------------ 620
KAJ6783033.1    ------------------------------------------------------------ 633
KAJ6780076.1    ------------------------------------------------------------ 577
KAJ6789215.1    ------------------------------------------------------------ 751
KAJ6787434.1    ------------------------------------------------------------ 574
KFG85702.1      ------------------------------------------------------------ 579
KAJ6780083.1    ------------------------------------------------------------ 579


KAJ6780494.1    GKVVPNSITPTTGDGVFDAFIANATALYNDQ----------------------------- 661
KAJ6784096.1    GNQTFGAMKPA--GGFFEEEEFQTVMGIVGKRLHWDTIAVEAPLGKLGGFTGMLRNLTLT 845
KAJ6785798.1    GPKTFASAFNEKGDIIFSKEEFLTEMSYYGQPLRWHEMAIEAPIHQITGWQGKVRDFTLT 706
KAJ6785933.1    ------------------------------------------------------------ 514
KFG80340.1      ------------------------------------------------------------ 620
KAJ6783033.1    ------------------------------------------------------------ 633
KAJ6780076.1    ------------------------------------------------------------ 577
KAJ6789215.1    ------------------------------------------------------------ 751
KAJ6787434.1    ------------------------------------------------------------ 574
KFG85702.1      ------------------------------------------------------------ 579
KAJ6780083.1    ------------------------------------------------------------ 579


KAJ6780494.1    -----------------------------------        661
KAJ6784096.1    NRG--------------------------------        848
KAJ6785798.1    TDGMGDKPGDSFATRGLPSAPILIAALAVALCLGL        741
KAJ6785933.1    -----------------------------------        514
KFG80340.1      -----------------------------------        620
KAJ6783033.1    -----------------------------------        633
KAJ6780076.1    -----------------------------------        577
KAJ6789215.1    -----------------------------------        751
KAJ6787434.1    -----------------------------------        574
KFG85702.1      -----------------------------------        579
KAJ6780083.1    -----------------------------------        579
```

Figure S3. Clustal alignment of *A. album* GH20 enzymes. The nine sequences were aligned together with the GH20 β-N-acetylhexosaminidases identified in *M. anisopliae* (KFG803409 and KFG85702). Boxed sequences correspond to the conserved motif H(N)XGA(C/G/M)DEA(I/L/V/Y), typical of GH20 sequences.

Figure S4

```
KAJ6781485.1     ------------------------------------------------------------ 0
BAD08218.2       ------------------------------------------------------------ 0
ADG96019.1       ------------------------------------------------------------ 0
BAA92250.1       ------------------------------------------------------------ 0
ABZ88800.1       ------------------------------------------------------------ 0
EAL84291.1       ------------------------------------------------------------ 0
AAO41660.1       ------------------------------------------------------------ 0
CAE54966.1       ------------------------------------------------------------ 0
AJG44374.1       ------------------------------------------------------------ 0
KAJ6787168.1     ------------------------------------------------------------ 0
ABY77913.1       ------------------------------------------------------------ 0
KAJ6783315.1     ------------------------------------------------------------ 0
AFG33049.1       ------------------------------------------------------------ 0
KAJ6786595.1     ------------------------------------------------------------ 0
KAJ6788818.1     ------------------------------------------------------------ 0
ABX57824.1       ------------------------------------------------------------ 0
BAA12799.1       ------------------------------------------------------------ 0
KAJ6781651.1     ------------------------------------------------------------ 0
KAJ6785337.1     MLESFEGDDADSDVDSAAAMATILWGRAVEQEDDILLWSLSSILNLHELGLASSHELGIA 60
KAJ6783761.1     ------------------------------------------------------------ 0


KAJ6781485.1     ------------------------------------------------------------ 0
BAD08218.2       ------------------------------------------------------------ 0
ADG96019.1       ------------------------------------------------------------ 0
BAA92250.1       ------------------------------------------------------------ 0
ABZ88800.1       ------------------------------------------------------------ 0
EAL84291.1       ------------------------------------------------------------ 0
AAO41660.1       ------------------------------------------------------------ 0
CAE54966.1       ------------------------------------------------------------ 0
AJG44374.1       ------------------------------------------------------------ 0
KAJ6787168.1     ------------------------------------------------------------ 0
ABY77913.1       ------------------------------------------------------------ 0
KAJ6783315.1     ------------------------------------------------------------ 0
AFG33049.1       ------------------------------------------------------------ 0
KAJ6786595.1     ------------------------------------------------------------ 0
KAJ6788818.1     ------------------------------------------------------------ 0
ABX57824.1       ------------------------------------------------------------ 0
BAA12799.1       ------------------------------------------------------------ 0
KAJ6781651.1     ------------------------------------------------------------ 0
KAJ6785337.1     GPPASMAEIAPIFAPSSFDAIQVKINSGIGHLSYLHSQLLAHDPSYHSNFEQGLVESALS 120
KAJ6783761.1     ------------------------------------------------------------ 0


KAJ6781485.1     ------------------------------------------------------------ 0
BAD08218.2       ------------------------------------------------------------ 0
ADG96019.1       ------------------------------------------------------------ 0
BAA92250.1       ------------------------------------------------------------ 0
ABZ88800.1       ------------------------------------------------------------ 0
EAL84291.1       ------------------------------------------------------------ 0
AAO41660.1       ------------------------------------------------------------ 0
CAE54966.1       ------------------------------------------------------------ 0
AJG44374.1       ------------------------------------------------------------ 0
KAJ6787168.1     ------------------------------------------------------------ 0
ABY77913.1       ------------------------------------------------------------ 0
KAJ6783315.1     ------------------------------------------------------------ 0
AFG33049.1       ------------------------------------------------------------ 0
KAJ6786595.1     ------------------------------------------------------------ 0
KAJ6788818.1     ------------------------------------------------------------ 0
ABX57824.1       ------------------------------------------------------------ 0
BAA12799.1       ------------------------------------------------------------ 0
KAJ6781651.1     ------------------------------------------------------------ 0
KAJ6785337.1     ARTAIMFSVTYFQQSHRYISLVHRPSFGSQDTSTALLLAVAFAGSTRSPPKDDVLCVRSL 180
KAJ6783761.1     ------------------------------------------------------------ 0


KAJ6781485.1     ------------------------------------------------------------ 0
```

```
BAD08218.2      ------------------------------------------------------------ 0
ADG96019.1      ------------------------------------------------------------ 0
BAA92250.1      ------------------------------------------------------------ 0
ABZ88800.1      ------------------------------------------------------------ 0
EAL84291.1      ------------------------------------------------------------ 0
AAO41660.1      ------------------------------------------------------------ 0
CAE54966.1      ------------------------------------------------------------ 0
AJG44374.1      ------------------------------------------------------------ 0
KAJ6787168.1    ------------------------------------------------------------ 0
ABY77913.1      ------------------------------------------------------------ 0
KAJ6783315.1    ------------------------------------------------------------ 0
AFG33049.1      ------------------------------------------------------------ 0
KAJ6786595.1    ------------------------------------------------------------ 0
KAJ6788818.1    ------------------------------------------------------------ 0
ABX57824.1      ------------------------------------------------------------ 0
BAA12799.1      ------------------------------------------------------------ 0
KAJ6781651.1    ------------------------------------------------------------ 0
KAJ6785337.1    ARLLEEFVFQHLERLIVNANSMNRSRELLETLAAAILVYQIQFMSMAAAALQRMRTSRLP 240
KAJ6783761.1    ------------------------------------------------------------ 0

KAJ6781485.1    ------------------------------------------------------------ 0
BAD08218.2      ------------------------------------------------------------ 0
ADG96019.1      ------------------------------------------------------------ 0
BAA92250.1      ------------------------------------------------------------ 0
ABZ88800.1      ------------------------------------------------------------ 0
EAL84291.1      ------------------------------------------------------------ 0
AAO41660.1      ------------------------------------------------------------ 0
CAE54966.1      ------------------------------------------------------------ 0
AJG44374.1      ------------------------------------------------------------ 0
KAJ6787168.1    ------------------------------------------------------------ 0
ABY77913.1      ------------------------------------------------------------ 0
KAJ6783315.1    ------------------------------------------------------------ 0
AFG33049.1      ------------------------------------------------------------ 0
KAJ6786595.1    ------------------------------------------------------------ 0
KAJ6788818.1    ------------------------------------------------------------ 0
ABX57824.1      ------------------------------------------------------------ 0
BAA12799.1      ------------------------------------------------------------ 0
KAJ6781651.1    ------------------------------------------------------------ 0
KAJ6785337.1    ALISAARKMGLFTTKHKAGLGSVVISADLSLTMPHMATALFTAVARWQKLWQENLRALSG 300
KAJ6783761.1    ------------------------------------------------------------ 0

KAJ6781485.1    ------------------------------------------------------------ 0
BAD08218.2      ------------------------------------------------------------ 0
ADG96019.1      ------------------------------------------------------------ 0
BAA92250.1      ------------------------------------------------------------ 0
ABZ88800.1      ------------------------------------------------------------ 0
EAL84291.1      ------------------------------------------------------------ 0
AAO41660.1      ------------------------------------------------------------ 0
CAE54966.1      ------------------------------------------------------------ 0
AJG44374.1      ------------------------------------------------------------ 0
KAJ6787168.1    ------------------------------------------------------------ 0
ABY77913.1      ------------------------------------------------------------ 0
KAJ6783315.1    ------------------------------------------------------------ 0
AFG33049.1      ------------------------------------------------------------ 0
KAJ6786595.1    ------------------------------------------------------------ 0
KAJ6788818.1    ------------------------------------------------------------ 0
ABX57824.1      ------------------------------------------------------------ 0
BAA12799.1      ------------------------------------------------------------ 0
KAJ6781651.1    ------------------------------------------------------------ 0
KAJ6785337.1    KAIKKGGIEKYPTCSFFCIWAENQTTPSCQQNILNTKLKSKAKAALSAIANYPQLHLLEL 360
KAJ6783761.1    ------------------------------------------------------------ 0

KAJ6781485.1    ------------------------------------------------------------ 0
BAD08218.2      ------------------------------------------------------------ 0
ADG96019.1      ------------------------------------------------------------ 0
BAA92250.1      ------------------------------------------------------------ 0
```

```
ABZ88800.1      ------------------------------------------------------------ 0
EAL84291.1      ------------------------------------------------------------ 0
AAO41660.1      ------------------------------------------------------------ 0
CAE54966.1      ------------------------------------------------------------ 0
AJG44374.1      ------------------------------------------------------------ 0
KAJ6787168.1    ------------------------------------------------------------ 0
ABY77913.1      ------------------------------------------------------------ 0
KAJ6783315.1    ------------------------------------------------------------ 0
AFG33049.1      ------------------------------------------------------------ 0
KAJ6786595.1    ------------------------------------------------------------ 0
KAJ6788818.1    ------------------------------------------------------------ 0
ABX57824.1      ------------------------------------------------------------ 0
BAA12799.1      ------------------------------------------------------------ 0
KAJ6781651.1    ------------------------------------------------------------ 0
KAJ6785337.1    DLASSTSIMTFAAEFAKQFNGQINIFIKNPGGVPDNHDHSEDGYERQFSMNYLGQFQLFS 420
KAJ6783761.1    ------------------------------------------------------------ 0

KAJ6781485.1    ------------------------------------------------------------ 0
BAD08218.2      ------------------------------------------------------------ 0
ADG96019.1      ------------------------------------------------------------ 0
BAA92250.1      ------------------------------------------------------------ 0
ABZ88800.1      ------------------------------------------------------------ 0
EAL84291.1      ------------------------------------------------------------ 0
AAO41660.1      ------------------------------------------------------------ 0
CAE54966.1      ------------------------------------------------------------ 0
AJG44374.1      ------------------------------------------------------------ 0
KAJ6787168.1    ------------------------------------------------------------ 0
ABY77913.1      ------------------------------------------------------------ 0
KAJ6783315.1    ------------------------------------------------------------ 0
AFG33049.1      ------------------------------------------------------------ 0
KAJ6786595.1    ------------------------------------------------------------ 0
KAJ6788818.1    ------------------------------------------------------------ 0
ABX57824.1      ------------------------------------------------------------ 0
BAA12799.1      ------------------------------------------------------------ 0
KAJ6781651.1    ------------------------------------------------------------ 0
KAJ6785337.1    MLKDALLSSATAASPSRLINVSSTRHKRGPITDFGIINVKKEDYDAFDSYCQSKTAAVYL 480
KAJ6783761.1    ------------------------------------------------------------ 0

KAJ6781485.1    ------------------------------------------------------------ 0
BAD08218.2      ------------------------------------------------------------ 0
ADG96019.1      ------------------------------------------------------------ 0
BAA92250.1      ------------------------------------------------------------ 0
ABZ88800.1      ------------------------------------------------------------ 0
EAL84291.1      ------------------------------------------------------------ 0
AAO41660.1      ------------------------------------------------------------ 0
CAE54966.1      ------------------------------------------------------------ 0
AJG44374.1      ------------------------------------------------------------ 0
KAJ6787168.1    ------------------------------------------------------------ 0
ABY77913.1      ------------------------------------------------------------ 0
KAJ6783315.1    ------------------------------------------------------------ 0
AFG33049.1      ------------------------------------------------------------ 0
KAJ6786595.1    ------------------------------------------------------------ 0
KAJ6788818.1    ------------------------------------------------------------ 0
ABX57824.1      ------------------------------------------------------------ 0
BAA12799.1      ------------------------------------------------------------ 0
KAJ6781651.1    ------------------------------------------------------------ 0
KAJ6785337.1    ASEMERRYGAPRIFTDVAEAAISSDVLHKDMRGRYLEDVFVSIPAAETKYPDILGYAEHT 540
KAJ6783761.1    ------------------------------------------------------------ 0

KAJ6781485.1    -------------------------------------------MLVKSI-------ASLA 10
BAD08218.2      -------------------------------------------MPIKSF-------ASRL 10
ADG96019.1      ---------------------------------------------MMTY-------SRLI 8
BAA92250.1      --------------------------------------------M---H-------FAGI 6
ABZ88800.1      --------------------------------------------M---R-------LSEI 6
EAL84291.1      --------------------------------------------M---R-------LSEI 6
AAO41660.1      --------------------------------------------M---R-------LSEI 6
```

```
CAE54966.1       --------------------------------------------M---R-------LSEI 6
AJG44374.1       -------------------------------------------MR---Y-------LATA 7
KAJ6787168.1     --------------------------------------------M---L-------FSLS 6
ABY77913.1       ------------------------------------------------------------ 0
KAJ6783315.1     -------------------------------------------MR---S-------ATVI 7
AFG33049.1       --------------------------------------------MKTAA-------ITSL 9
KAJ6786595.1     ------------------------------------------------------------ 0
KAJ6788818.1     ---------------------------------------------MASL-------RSTL 8
ABX57824.1       ---------------------------------------------MPSL-------RNTL 8
BAA12799.1       ---------------------------------------------MPSL-------RNTL 8
KAJ6781651.1     ---------------------------------------------MKTT-------ASIL 8
KAJ6785337.1     YDEQRAKDLWKFTKELLGHDTGLFPYAFTSFNTVAAQYRDIPDMMRVTT-------KIAL 593
KAJ6783761.1     -----------------------------------------------MK-------ASLA 6


KAJ6781485.1     ALSGIAAAAGPIRASVPTNVDGSKYN---KPNAGPPGDWFSGDSSLPISKIASA---V-S 63
BAD08218.2       A-LSLA----ICGTAMGQKVNGADYN---KPDGGPPAKFFQASSSIPVAAIQAA---A-A 58
ADG96019.1       PFALFA----LFGTGLAQTVDGSKFN---KPDGGPPGSYFAAGSSIPVAALQSA---A-A 57
BAA92250.1       VAIALA--TGATAYDLPENLKQ-IYE---KH-KSGKCSK----------ELQGG---YDN 46
ABZ88800.1       LTVALV--TGATAYNLPNNLEQ-IYD---KH-K-GKCSK----------VLAKG---FTN 45
EAL84291.1       LTVALV--TGATAYNLPNNLKQ-IYD---KH-K-GKCSK----------VLAKG---FTN 45
AAO41660.1       LTVALV--TGATAYNLPNNLKQ-IYD---KH-K-GKCSK----------VLAKG---FTN 45
CAE54966.1       LTVALV--TGATAYNLPNNLKQ-IYD---KH-K-GKCSK----------ILAKG---FTN 45
AJG44374.1       AVLAGA--GLASAYSVPANLQQ-IYN---KH-KTGTCQN----------KLQDG---FSD 47
KAJ6787168.1     TVAALG--AVASAYDLPDNLRS-LYQ---AH-INGECRS----------ALSDP---FNT 46
ABY77913.1       -------------YQLPANLKK-IYD---SH-KSGTCSK----------KLSSA---FSG 29
KAJ6783315.1     TLAALG--SLASGYQLPAKLKS-LYD---QH-KSGSCSK----------KLAGP---FNG 47
AFG33049.1       AVAALA--APALAYDIPDNVQA-FYD---KVKKGGDCTG--------SDLLKGG---FYA 52
KAJ6786595.1     -------------------MRA-LVN---I-------TW----------HIVGAQLARNA 20
KAJ6788818.1     LLSL-A--AVAAARDVPSNVRD-FYN---SVRGKGQCSN----------KLKSG---FHS 48
ABX57824.1       LASLLA--ASVSARDVPANVKS-FKD---SIIKQGSCKS----------TLATG---FYS 49
BAA12799.1       LASLLA--ASVSGRDVPANVKT-FKD---SIIKQGSCKS----------TLATG---FFS 49
KAJ6781651.1     FASLLA--SPVLGRQVPQNVRD-LYN---SIRAQKSCHN----------ELKGG---FYS 49
KAJ6785337.1     TASLLA--LNSYARDVPSNIRD-LYN---SIRARGQCSN----------VLKGG---FYS 634
KAJ6783761.1     ILSLLA--TGALSREVPANVKS-LYK---SIRSKGQCSK----------VLKTG---FFS 47
                                    :     .                        :

KAJ6781485.1     KMSKTPKDASYILSND-NHKKATIHSDWANLSKGA-AYAFVADMDVDCDGIDSH------115
BAD08218.2       KASKVPSHATYPIG--QGSTKSTIHSDWAGFSEGA-AFSFIADMDVDCDGLNHG------109
ADG96019.1       KARTPVPDATYPINGDNGAKKVTIHSDWAKFDKGA-AYVWIADMDVDCDGIDYK------110
BAA92250.1       GHSHDGKSFSY--CGDIP---------NAIYLHSSKNGGQYADMDIDCDGANRH---AGK 92
ABZ88800.1       GDASQGKSFSY--CGDIP---------GAIFISSSK---GYTNMDINCDGANNS---AGK 88
EAL84291.1       GDASQGKSFSY--CGDIP---------GAIFISSSK---GYTNMDIDCDGANNS---AGK 88
AAO41660.1       GDASQGKSFSY--CGDIP---------GAIFISSSK---GYTNMDIDCDGANNS---AGK 88
CAE54966.1       GDASQGKSFSY--CGDIP---------GAIFISSSK---GYTNMDIDCDGANNS---AGK 88
AJG44374.1       GISGPGT-SAY--CGDIQ---------GAIFLHSSANGGQYDNMDIDCDGANNS---GGD 92
KAJ6787168.1     G-------AVY--CGDIP---------NAIFLKG--PNGEYDNMDIDCDGANNS---AGD 83
ABY77913.1       G-------AVY--CGDLP---------NAIFLKG--KAGNYDNLDIDCDGANNS---AGE 66
KAJ6783315.1     G-------ASY--CGDIN---------GAIFLKG--N-GNYDNMDIDCDGANRT---GGA 83
AFG33049.1       -EEGGSKNFGY--CQKYF------TNKG-FYLKG--SGSKLADMDIDCDGDQDG--IDSR 98
KAJ6786595.1     -DQITRADMSY--CGDHL------DDLGVVYLQG--P-GKFSDMDVDCDGAQGGPQDDGR 68
KAJ6788818.1     -IEGDDGSFSY--CGDHM------NDYRVLYLQG--KGGQLVNMDIDCDGIQHGPADDGR 97
ABX57824.1       -SDGDSGTYSY--CGDHV------KDYNVIYLQG--KNGKLVNMDIDCDGIQGSPADDGR 98
BAA12799.1       -SDGDSGTYSY--CGDHV------KDYNVIYLQG--KNGKLVNMDIDCDGVQGSPADDGR 98
KAJ6781651.1     -QEGDSKNFGY--CGDHL------SDYGIMYLQG--KNGQLVNMDIDCDGALGD--GDGS 96
KAJ6785337.1     -QEKDSKDFCY--CGDHL------ASDGIVYLQG--THGNLVNMDIDCDGALGE--GDGS 681
KAJ6783761.1     -SEDDSKDFSY--CGDHL------ESDGILYIQG--KGGKLTNMDIDCDGILSK--KHGE 94
                           *                      .        ::*::***

KAJ6781485.1     CKGNDD--GETHTDFG-----------ALAAYEVPFVVIPNKFASEHEK-------ELAG 155
BAD08218.2       CKGNPD--GQKETNWG-----------ALSAYEVPFIVIPQEFLDANKG-------TLKG 149
ADG96019.1       CKGNPD--GQHQTNFG-----------ALAAYEVPFFVIPDRFGTKYAK-------QLPG 150
BAA92250.1       CSNDHS--GQGETRWKDEVQKL--GIDDLDANIHPYVVFGNENDDGDDPEFDPRKHGMEP 148
ABZ88800.1       CANDPS--GQGETAFKSDVKKF--GISDLDANIHPYVVFGNED---HSPKFKPQSHGMQP 141
EAL84291.1       CANDPS--GQGETAFKSDVKKF--GISDLDANIHPYVVFGNED---HSPKFKPQSHGMQP 141
AAO41660.1       CANDPS--GQGETAFKSDVKKF--GISDLDANIHPYVVFGNED---HSPKFKPQSHGMQP 141
CAE54966.1       CANDPS--GQGETAFKSDVKKF--GISDLDANIHPYVVFGNED---HSPKFKPQSHGMQP 141
```

```
AJG44374.1      CANDPS--GQSMTAFMDTVKQY--GISDLDANIHPYVVFGNSG---SSPTFDPQQYGMQP 145
KAJ6787168.1    CANDPS--GQGQTSFVDTVSTF--GIQDLDANLHSYVVFGNEG---DSPSFSPQNHGVQP 136
ABY77913.1      CANDPS--GQGQTAFKDDVKKY--GIPDLDANLHPYVVFGNEE---HSPSFDPEAHGMER 119
KAJ6783315.1    CANDPS--GQGETSFKDTVKTY--GIPDLDANVHPYIVFGNEG---ASPSFNPQAHGIKP 136
AFG33049.1      CDSSGD--TQPDTAFKDQVKS-DYGIPDLRADIHPYVVLGNYGDY--SPTFDPEHVGIKP 153
KAJ6786595.1    CDASTD--TQDTTAIRWVIEGYDVGISDLNPHEHSFVVFGNSGTRPGWKTFSPRDVGVEP 126
KAJ6788818.1    CGSSGD--TQSITSFQGTVQGYGTGQKDLDAFIHPYVVFGNEGSKSGYPNFNPQDYGVEP 155
ABX57824.1      CGSSGD--TQSITSFQWVLESYGTSQKDLDANIHPYIVFGNEGTKSGWKTFDPEKHGIKP 156
BAA12799.1      CGSSGD--TQSITSFQWVLESYGTSQKDLDANIHPYIVFGNEGTKKGWKTFDPEKHGIKP 156
KAJ6781651.1    CDSSTD--TQGQTTFQGDVEGYGKGIKDLNAYIHSFVVLGNDGSKNGYVTFKPEQYGVEP 154
KAJ6785337.1    CDSSTD--TQGQTTFGDIVKGYKKGIKDLNAYIHSFVVLGNEGKKKGYVEFDPQSVGIEP 739
KAJ6783761.1    CDSSGD--IQYETRFRKEVQGYKKGLKDLDAYVHSYVVLGNEGKKNNYIEFNPQSVGIEP 152
                *  . .   :  *               *      :.*: :                :

KAJ6781485.1    NNLVAVICNGKL------YYGVFGDTDGDSPQ--EIGEASWLMANTCFPGEGLNGG---- 203
BAD08218.2      NAVAAVICNGKM------FYGIFGDSNGDSPQ--VTGEASWLMARTCFPKEDLNGN---- 197
ADG96019.1      NNVGAVICNGKM------FYGIYGDSDGDTPQ--VIGEASWLMARTCFPNDDLNGN---- 198
BAA92250.1      LSVMAVVCNKKL------FYGIWGDTNGHT----ATGEASLSMAELCFPEEDPSGD---- 194
ABZ88800.1      LSVMAVVCNGQL------HYGIWGDTNGGV----STGEASISLADLCFPNEHLDGN---- 187
EAL84291.1      LSVMAVVCNGQL------HYGIWGDTNGGV----STGEASISLADLCFPNEHLDGN---- 187
AAO41660.1      LSVMAVVCNGQL------HYGIWGDTNGGV----STGEASISLADLCFPNEHLDGN---- 187
CAE54966.1      LSVMAVVCNGQL------HYGIWGDTNGGV----STGEASISLADLCFPNEHLDGN---- 187
AJG44374.1      LSVMAVVCNNQL------FYGVWGDTNGDI----ATGEASISLAKLCFPNDGITGD---- 191
KAJ6787168.1    LSVMAVVCNNQV------FYGVWGDTNGFT----STGEASLALAKLCFPNDGLNGD---- 182
ABY77913.1      LSVMAIVCNNQV------FYGIWGDTNGGT----STGEASLALGKLRFPNDGLNGD---- 165
KAJ6783315.1    LSVMAVVCNGQL------HYGIWGDTNGFT----STGEASLSMGKLCFPNEHLTGN---- 182
AFG33049.1      LSLVAVVCNNKL------IYGIWGDTNGDDGP-PLIGESSLAVGTACFGD-SINGD---- 201
KAJ6786595.1    ASLMAVICGDQMKSARIKFYGIWGDSNTDDGPRPSVGEVSISLGTACYGK-HMQAGTGSD 185
KAJ6788818.1    LSVMAVVCGNKL------IYGIWGDTNGDDGPQAMVGEASISLATACYGN-GINGN---- 204
ABX57824.1      LSVMAVVCGNKM------FYGIWGDENGDDGDYPMVGEASISLATACFGK-SMNGN---- 205
BAA12799.1      LSVMAVVCGNKM------FYGIWGDENGDDGDQPMVGEASISLATACFGK-SMNGN---- 205
KAJ6781651.1    LSLVAVVCGDKM------FYGVWGDTNGDDGP-PLVGEVSDSLGRACYGN-AVNGN---- 202
KAJ6785337.1    LSIVAVVCGDRL------LYGVWGDTNGDDGP-PLIGEVSDSLGRACYGD-EVNGN---- 787
KAJ6783761.1    LSVVAVVCGDKM------FYGVWGDTNGDDGP-PLVGEASLTIGQACYGK-SINGN---- 200
                  : *::*. ::       **::** :         ** *  :.   :      ..

KAJ6781485.1    QGHSDADVTYIFFTGDDSVLPDNAI---GKNYLTNFQALKNLGDKLMRDLVSSLHL---- 256
BAD08218.2      KGHTAADVTYIVFTGDKAVLPSSAL---NKNYITNFDTLRSMGDSLVGALAKNLNLGGGG 254
ADG96019.1      SGHGDVDVTYILFTGDDSVLPSSAL---NKNYVTNFTTLRSMGDKLMTALAKNLKLVDGG 255
BAA92250.1      SGHEPNDVLYIGFTGKEAVPGKS-ADWKADSTESFEESIKELGDKLVAGLK-------A- 245
ABZ88800.1      HGHDPNDVLFIGFTSKDAVPGAT-AKWKAKNAKEFEDSIKSIGDKLVAGLK-------A- 238
EAL84291.1      HGHDPNDVLFIGFTSKDAVPGAT-AKWKAKNAKEFEDSIKSIGDKLVAGLK-------A- 238
AAO41660.1      HGHDPNDVLFIGFTSKDAVPGAT-AKWKAKNAKEFEDSIKSIGDKLVAGLK-------A- 238
CAE54966.1      HGHDPNDVLFIGFTSKDAVPGAT-AKRKAKNAKEFEDSIKSIGDKLVAGLK-------A- 238
AJG44374.1      NGHDQDDVLYIGFTGQDTVPGAS-AAWTASDTSTFEESIKGLGDRLVAKLS-------A- 242
KAJ6787168.1    AGHDQKDVMYIAFTGDEAVPGRDGAKWSAGSPEEFEDSIKALGDRLVASLP-------S- 234
ABY77913.1      NGHDPKDVLYIGFTGSGAVPGKSGANWKAKKTADFEASIKALGDKLVASLP-------A- 217
KAJ6783315.1    NGHDPKDVLYIAFTGADTVPGKNGANWKAKNTDDFAKSIKSLGDKLVGRL---------- 232
AFG33049.1      NGHEGTDVLYIAFKGDDAVPGT-SADWSASSYEDFAASIKDFGDNLVAS----------- 249
KAJ6786595.1    TSHTDPDVLYIAFKGEDAVPGKGGANWTAGNFDDFHASLVGLGDKLISRLE-------GD 238
KAJ6788818.1    SGHDDNDVLFIAFPGHDAVPGKNGAKWHASSYNEFANSITSLGDKLIQRIG-------GG 257
ABX57824.1      FGHGEDDVLYIAFPGSDAVPGAKGAKWTAKNFDEFQSSITTLGDKLIKRIG-------SS 258
BAA12799.1      FGHSDDDVLYIAFPGADAVPGAKGAKWNAKNFDEFQTSITSLGDKLIKRIG-------GT 258
KAJ6781651.1    QAHDDNDILYIAFTGSDAVPGANGAKWNANSFSSFESSLGSLGDRLVQRIG-------GG 255
KAJ6785337.1    EAHDDNDVLYIAFTGKGAVPGANGAKWNAESFDEFEASLQAQGDKLVAKIG-------GG 840
KAJ6783761.1    NGHDENDVLYIAFRGNQAVPGANGAAWNAKTFTEFENSLAKIGDKLVKGIK-------G- 252
                 .*   *: :* * .  :*                  ::   ** *:

KAJ6781485.1    ------------------------------------------------------------ 256
BAD08218.2      GNPPTTLTTTSIPEPTGGSGSCSWPGHCAGATCSSNDDCSDDLTCQNGKCASDGSAETCS 314
ADG96019.1      DGGSPTTTA----GSNPTSGSCEWEGHCAGASCKDENDCSDQLVCKSGKCPSD------- 304
BAA92250.1      ------------------------------------------------------------ 245
ABZ88800.1      ------------------------------------------------------------ 238
EAL84291.1      ------------------------------------------------------------ 238
AAO41660.1      ------------------------------------------------------------ 238
CAE54966.1      ------------------------------------------------------------ 238
AJG44374.1      ------------------------------------------------------------ 242
```

```
KAJ6787168.1      ------------------------------------------------------------ 234
ABY77913.1        ------------------------------------------------------------ 217
KAJ6783315.1      ------------------------------------------------------------ 232
AFG33049.1        ------------------------------------------------------------ 249
KAJ6786595.1      NGG----------------DGCSWDGHCAGASCTRAQDCADQLTCVKGKCA--------- 273
KAJ6788818.1      GGGNP----------PPSGGNCSWAGHCEGASCKNENDCSDSLVCKSGKCSRG------- 300
ABX57824.1        SGGGT-----------TPPPTCSWPGHCQGATCKTGDDCSDDLICTNGKCSPP------- 300
BAA12799.1        NNGGGD-------TGGGSGNTCSWPGHCQGAACKTGDDCSDDLICTKGKCSPP------- 304
KAJ6781651.1      GGS-NP-------GNPGNPPSCSWQGHCLGAKCSSDDDCDGNLQC-----------KS-- 294
KAJ6785337.1      DTGSNPTSTTASPGRPTNSPPPN--NENLNPKCAPGGNFDLGYFNLQLPVGGPDIIKSKQ 898
KAJ6783761.1      ------------------------------------------------------------ 252


KAJ6781485.1      ------------------------------------------------------------ 256
BAD08218.2        WEGHCKGATCSS--NDDCSDELACIS-G-----ICSVDNGVETCE--------------- 351
ADG96019.1        ------------------------------------------------------------ 304
BAA92250.1        ------------------------------------------------------------ 245
ABZ88800.1        ------------------------------------------------------------ 238
EAL84291.1        ------------------------------------------------------------ 238
AAO41660.1        ------------------------------------------------------------ 238
CAE54966.1        ------------------------------------------------------------ 238
AJG44374.1        ------------------------------------------------------------ 242
KAJ6787168.1      ------------------------------------------------------------ 234
ABY77913.1        ------------------------------------------------------------ 217
KAJ6783315.1      ------------------------------------------------------------ 232
AFG33049.1        ------------------------------------------------------------ 249
KAJ6786595.1      ------------------------------------------------------------ 273
KAJ6788818.1      ------------------------------------------------------------ 300
ABX57824.1        ------------------------------------------------------------ 300
BAA12799.1        ------------------------------------------------------------ 304
KAJ6781651.1      -HK-CSQ----------------------------------------------------- 299
KAJ6785337.1      LQG-CNGYTGDTFFTDKSSGEMVLLAPGNPDLTGCATTSGSAHCRTELREVIHSSGANAA 957
KAJ6783761.1      ------------------------------------------------------------ 252


KAJ6781485.1      ------------------------------------------------------------ 256
BAD08218.2        WEGHCEGASCSS---HDDCDGNLACKNGKCSA---------------------------- 380
ADG96019.1        ------------------------------------------------------------ 304
BAA92250.1        ------------------------------------------------------------ 245
ABZ88800.1        ------------------------------------------------------------ 238
EAL84291.1        ------------------------------------------------------------ 238
AAO41660.1        ------------------------------------------------------------ 238
CAE54966.1        ------------------------------------------------------------ 238
AJG44374.1        ------------------------------------------------------------ 242
KAJ6787168.1      ------------------------------------------------------------ 234
ABY77913.1        ------------------------------------------------------------ 217
KAJ6783315.1      ------------------------------------------------------------ 232
AFG33049.1        ------------------------------------------------------------ 249
KAJ6786595.1      ------------------------------------------------------------ 273
KAJ6788818.1      ------------------------------------------------------------ 300
ABX57824.1        ------------------------------------------------------------ 300
BAA12799.1        ------------------------------------------------------------ 304
KAJ6781651.1      ------------------------------------------------------------ 299
KAJ6785337.1      WSPKGTNTLTVTMKVVKSDDGTHGTAIGQVFASEASKPLAEMYYSPSGDIVVGVKPDEDS 1017
KAJ6783761.1      ------------------------------------------------------------ 252


KAJ6781485.1      ------------------------------------------------------------ 256
BAD08218.2        ------------------------------------------------------------ 380
ADG96019.1        ------------------------------------------------------------ 304
BAA92250.1        ------------------------------------------------------------ 245
ABZ88800.1        ------------------------------------------------------------ 238
EAL84291.1        ------------------------------------------------------------ 238
AAO41660.1        ------------------------------------------------------------ 238
CAE54966.1        ------------------------------------------------------------ 238
AJG44374.1        ------------------------------------------------------------ 242
KAJ6787168.1      ------------------------------------------------------------ 234
ABY77913.1        ------------------------------------------------------------ 217
KAJ6783315.1      ------------------------------------------------------------ 232
```

```
AFG33049.1      ------------------------------------------------------------ 249
KAJ6786595.1    ------------------------------------------------------------ 273
KAJ6788818.1    ------------------------------------------------------------ 300
ABX57824.1      ------------------------------------------------------------ 300
BAA12799.1      ------------------------------------------------------------ 304
KAJ6781651.1    ------------------------------------------------------------ 299
KAJ6785337.1    NQVVTKLGNVSVGTKFTYVLNYSDDKLSISINGKTTALDTFNWDSPACYFKSGNYNQGKS 1077
KAJ6783761.1    ------------------------------------------------------------ 252


KAJ6781485.1    ----------------  256
BAD08218.2      ----------------  380
ADG96019.1      ----------------  304
BAA92250.1      ----------------  245
ABZ88800.1      ----------------  238
EAL84291.1      ----------------  238
AAO41660.1      ----------------  238
CAE54966.1      ----------------  238
AJG44374.1      ----------------  242
KAJ6787168.1    ----------------  234
ABY77913.1      ----------------  217
KAJ6783315.1    ----------------  232
AFG33049.1      ----------------  249
KAJ6786595.1    ----------------  273
KAJ6788818.1    ----------------  300
ABX57824.1      ----------------  300
BAA12799.1      ----------------  304
KAJ6781651.1    ----------------  299
KAJ6785337.1    ATSSEVRIYSIDVVHK  1093
KAJ6783761.1    ----------------  252
```

Figure S4. Clustal alignment of *A. album* GH75 enzymes. The eight sequences were aligned together with the GH75 chitosanases characterized in fungi (http://www.cazy.org/GH75_characterized.html) with the exception of the sequence EAQ87617 which is not complete. Boxed sequences correspond to the conserved motifs N(D)M(L)DIDCD and YGIWGD, typical of GH75 sequences.

Figure S5

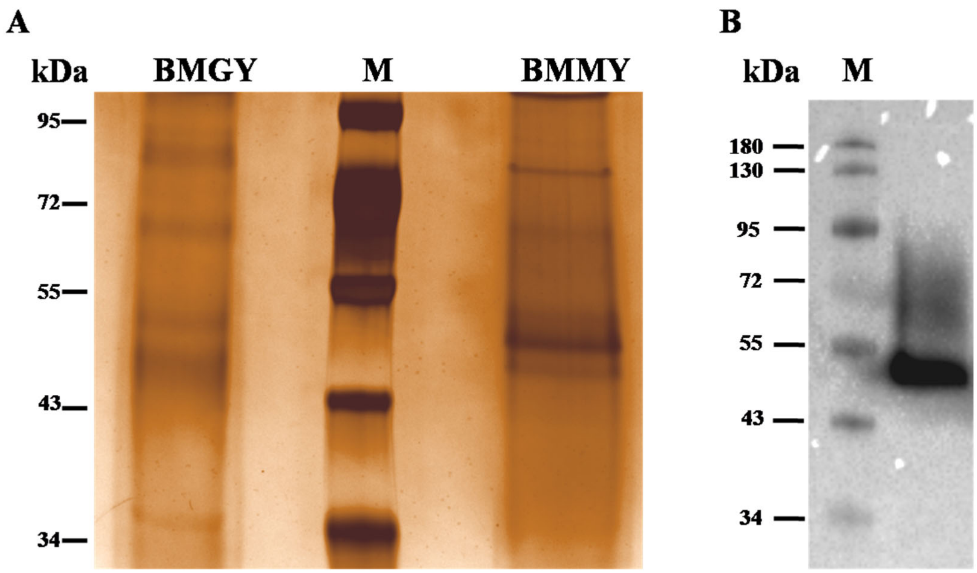

Figure S5. Expression of recombinant Chi1 mature protein. (A) SDS-PAGE on 15% biphasic polyacrylamide gel, M: Marker Protein (Thermo Scientific); BMGY (Buffered Glycerol-complex Medium): clone growth not induced with methanol; BMMY (Buffered Methanol-complex Medium): clone growth induced with methanol. Gel stained with silver staining. (B) Western blot of culture medium after expression in *Pichia pastoris* cells.

Figure S6

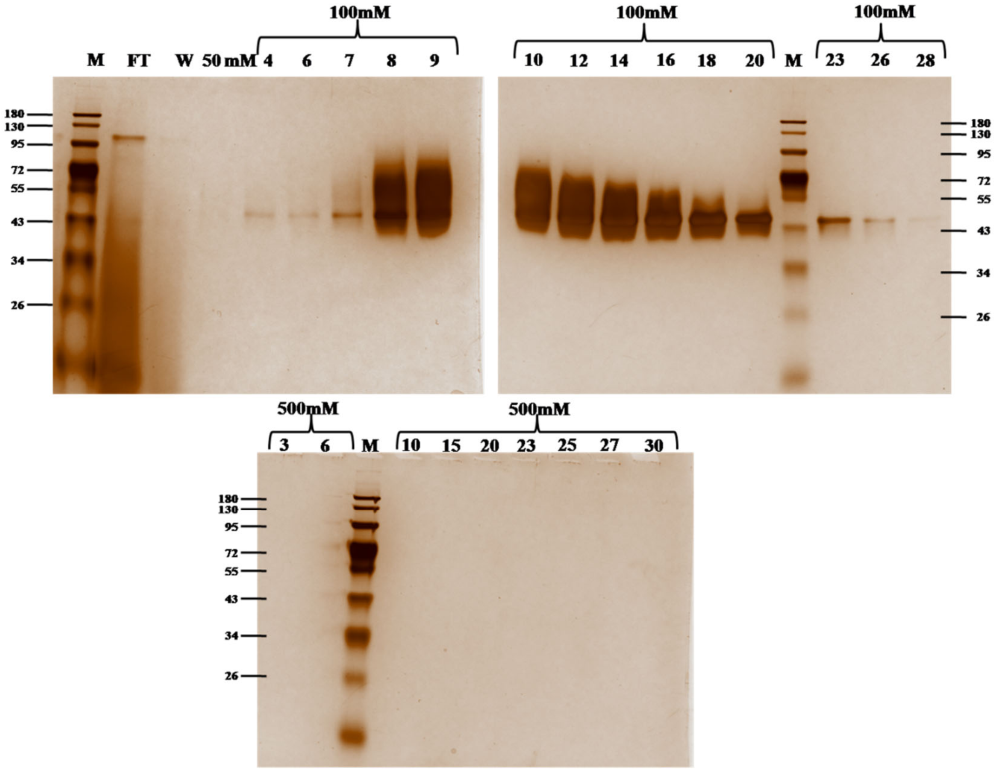

Figure S6. SDS-PAGE (15% polyacrylamide gel) analysis of chromatographic fractions. M: Marker PageRuler Prestained Protein Ladder (Thermo Scientific); FT: flow-through; W: washing fraction; 50 mM: fraction eluted with 50 mM imidazole EB; 100mM: fractions eluted with 100 mM imidazole EB; 500 mM: fractions eluted with 500 mM imidazole EB. Gels stained with silver staining.

Figure S7

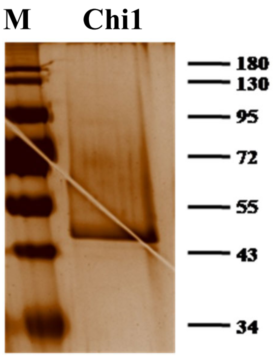

Figure S7. SDS-PAGE (15% polyacrylamide gel) of the purified Chi1 recombinant protein. M: Marker PageRuler Prestained Protein Ladder (Thermo Scientific). Chi1: purified recombinant Chi1 protein after dialysis. Gel stained with silver staining.
